# Supplementary material for: Digitally enabled aged care and neurological rehabilitation to enhance outcomes with Activity and MObility UsiNg Technology (AMOUNT) in Australia: A randomised controlled trial
Source: PLoS Med. 2020 Feb 18;17(2):e1003029. doi: 10.1371/journal.pmed.1003029 (PMC7028259; doi:10.1371/journal.pmed.1003029)
Supplement: S1 Table — (DOCX) [file pmed.1003029.s002.docx]

| S1 Table. Participant primary diagnosis at rehabilitation admission | | |
| --- | --- | --- |
|  | **Intervention (n=149)**  **n (%)** | **Control (n=151)**  **n (%)** |
| *Neurological*  Stroke/TIA  Traumatic/hypoxic brain injury  Other neurological conditions | 51 (34)  10 (7)  11 (7) | 47 (31)  11 (7)  19 (13) |
| *Cardiopulmonary*  Pulmonary health conditions  Cardiac health conditions | 9 (6)  7 (5) | 3 (2)  6 (4) |
| *Musculoskeletal*  Fall with fracture  Fall with no fracture  UL/Cx/Tx fracture, no fall specified  LL/pelvis/Lx fracture, no fall specified  LL amputation  Joint replacement  Orthopaedic pain | 2 (1)  3 (2)  2 (1)  18 (12)  5 (3)  3 (2)  8 (5) | 4 (3)  4 (3)  4 (3)  23 (15)  2 (1)  4 (3)  7 (5) |
| *Restorative care/other*  Deconditioning/functional decline  Infection/sepsis  Other | 9 (6)  6 (4)  5 (3) | 11 (7)  1 (1)  5 (3) |

TIA: transient ischaemic attack; UL: upper limb; LL: lower limb; Cx: cervical spine; Tx: thoracic spine; Lx: lumbar spine
