# Supplementary material for: Digitally enabled aged care and neurological rehabilitation to enhance outcomes with Activity and MObility UsiNg Technology (AMOUNT) in Australia: A randomised controlled trial
Source: PLoS Med. 2020 Feb 18;17(2):e1003029. doi: 10.1371/journal.pmed.1003029 (PMC7028259; doi:10.1371/journal.pmed.1003029)
Supplement: S2 Table — (DOCX) [file pmed.1003029.s003.docx]

| **S2 Table. Costs for digitally-enabled rehabilitation intervention** | | | |
| --- | --- | --- | --- |
| **Items** | **Description** | **Total cost** | **Cost / participant^1^** |
| **Training** | Costs with registration for staff to attend training course, staff time to attend training course, workshops and meetings | $32,894 | $230 |
| **Equipment** | Costs with equipment**^2^** used in the trial (eg activity monitors, Nintendo Wii, iPads, sim cards, consumables, maintenance and repairs) | $50,616 | $354 |
| **Intervention delivery - Inpatient** | Staff time**^3^** to deliver the intervention at the hospital (time taken on session delivery and preparation for session) | N/A | $621 (SD = 688, range 0 to 3,348) |
| **Intervention delivery - Outpatient** | Staff time**^3^** to deliver the intervention after hospital discharge (session delivery, phone call, email, in travel, home visit, video conference as well as preparation for treatment session) and costs with SMS**^4^**, phone call**^5^**, and travel.**^6^** | N/A | $ 687 (SD 302, range 0 to 2,240) |
| **Total cost** |  | N/A | $1,892 (SD 774, range 908 to 4399) |

^1^Considering 143 participants in the intervention group (excluded participants who died or dropped-out before the 3-week follow-up). All costs were valued in 2017 Australian dollars (AUD).

^2^The annual equivalent cost was calculated for each equipment and the value was divided by 2 (to reflect the 6-month trial duration). We assumed that all equipment used had no re-sale value.

^3^Staff costs were calculated according to staff level using actual data. The public hospital award wages for physiotherapists relevant for each hospital were used. Based on trial records and physiotherapists feedback, we considered the preparation time to be: 20 minutes for inpatient sessions, 30 minutes for home visits, 10 minutes for SMS, email, phone and video conference.

^4^Cost of SMS extracted from SMS Global (example of provider, [www.smsglobal.com](https://www.smsglobal.com/)): $0.10 / SMS.

^5^Costs estimated from OPTUS (example of provider, [www.optus.com.au](http://www.optus.com.au)) assuming that all calls were made to mobile numbers: $0.28 per minute plus $ 0.52 connection fee per call).

^6^Cost of travel extracted from Australian Tax Office and estimated as $0.66 per Km.
