# Supplementary material for: Digitally enabled aged care and neurological rehabilitation to enhance outcomes with Activity and MObility UsiNg Technology (AMOUNT) in Australia: A randomised controlled trial
Source: PLoS Med. 2020 Feb 18;17(2):e1003029. doi: 10.1371/journal.pmed.1003029 (PMC7028259; doi:10.1371/journal.pmed.1003029)
Supplement: S3 Table — (DOCX) [file pmed.1003029.s004.docx]

| S3 Table. Sensitivity analyses for primary outcomes | | | |
| --- | --- | --- | --- |
| **Outcome** | **Time Point or time between Assessments** | **Coefficient (95% CI); n** | **P value** |
| **Mobility** *(+ve MD favours intervention group)* |  |  |  |
| Short Physical Performance Battery (continuous version, 0-3)  not adjusting for baseline values  adjusting for stratification variables^#^ | 6 months minus baseline  6 months minus baseline | 0.2 (0.1 to 0.3); 254  0.2 (0.1 to 0.3); 254 | 0.006  0.007 |
| **Physical Activity** *(+ve MD favours intervention group)* |  |  |  |
| Proportion of the day spent upright, %  not adjusting for baseline values  adjusting for stratification variables^#^ | At 6 months  At 6 months | 0.03 (-2.5 to 2.6); 242  -1.2 (-5.3 to 2.8); 239 | 0.98  0.55 |

This analysis was conducted using linear regression models. Due to skewed distributions, the change score between timepoints was used for the Short Physical Performance Battery. Confidence intervals have not been adjusted for multiplicity so inferences drawn from the intervals may not be reproducible. ^#^Stratification variables: site and neurological versus non-neurological health conditions limiting mobility.
