# Supplementary material for: Digitally enabled aged care and neurological rehabilitation to enhance outcomes with Activity and MObility UsiNg Technology (AMOUNT) in Australia: A randomised controlled trial
Source: PLoS Med. 2020 Feb 18;17(2):e1003029. doi: 10.1371/journal.pmed.1003029 (PMC7028259; doi:10.1371/journal.pmed.1003029)
Supplement: S4 Table — (DOCX) [file pmed.1003029.s005.docx]

| S4 Table. Primary and secondary outcomes (additional analysis) | | | |
| --- | --- | --- | --- |
| **Outcome** | **Time between Assessments** | **Coefficient (95% CI); n** | **P value** |
| **Performance-based outcomes** |  |  |  |
| **Mobility** |  |  |  |
| Short Physical Performance Battery |  |  |  |
| Continuous (0-3) | 6 months minus 3 week | 0.0 (-0.1 to 0.1); 249^§^ | 1.00 |
| Total score (0-12) | 6 months minus 3 week | 0.1 (-0.4 to 0.7); 255^#,§^ | 0.68 |
| Balance subscale (0-4)~ | 6 months minus 3 week | 1.2 (0.7 to 2.1); 255 | 0.47 |
| Gait speed subscale (0-4)~ | 6 months minus 3 week | 1.0 (0.6 to 1.6); 255 | 0.97 |
| Chair stand subscale (0-4)~ | 6 months minus 3 week | 1.1 (0.7 to 1.7); 255 | 0.71 |
| de Morton Mobility Index (0-100) | 6 months minus 3 week | -0.1 (-3.7 to 3.5); 254 | 0.96 |
| Single leg stance, (0-10 s) | 6 months minus 3 week | 0.6 (-0.2 to 1.5); 254^§^ | 0.16 |
| Maximal balance range test, mm | 6 months minus 3 week | 3.8 (-9.7 to 17.4); 255 | 0.58 |
| Step test, steps, (average of both legs) | 6 months minus 3 week | 0.5 (-0.5 to 1.4); 255^§^ | 0.35 |
| **Physical Activity** |  |  |  |
| Time spent standing, min/day | 6 months minus 3 week | -4.0 (-28.4 to 20.4); 234 | 0.75 |
| Time spent stepping, min/day | 6 months minus 3 week | 5.0 (-2.7 to 12.6); 234^#*§^ | 0.20 |
| Number of steps per day | 6 months minus 3 week | 476 (-121 to 1073); 234^#,*^ | 0.12 |
| Number of sit to stands per day | 6 months minus 3 week | 2 (-1 to 5); 234^§^ | 0.24 |
| **Cognition**† |  |  |  |
| Trail Making Test A, s | 6 months minus 3 week | 2.8 (-1.4 to 7.0); 252 | 0.20 |
| Trail Making Test B, s | 6 months minus 3 week | 2.0 (-9.1 to 13.1); 251 | 0.76 |
| Trail Making Test B – A, s | 6 months minus 3 week | 0.1 (-9.2 to 9.3); 251^¶^ | 0.99 |
| **Participant Reported Outcome Measures** | | | |
| Incidental and Planned Exercise Questionnaire |  |  |  |
| Total score, h/wk | 6 months minus 12 week | 1.5 (-2.1 to 5.1); 241 | 0.42 |
| Home exercise subscale, h/wk | 6 months minus 12 week | 0.5 (-0.2 to 1.2); 241^#^ | 0.16 |
| Walking activity subscale, h/wk | 6 months minus 12 week | 1.5 (0.4 to 2.7); 241 | 0.01 |
| Modified Computer Self-Efficacy Scale (10-100), mean (SD) | 6 months minus 12 week | 2.5 (-2.4 to 7.4); 247^‡^ | 0.31 |
| Activities-specific Balance Confidence Scale (0-100) | 6 months minus 12 week | 2.5 (-1.6 to 6.7); 247 | 0.23 |
| WHO Disability Assessment Schedule 2.0 (raw score 12-60)† | 6 months minus 12 week | -0.7 (-2.2 to 0.8); 249^‡^ | 0.35 |
| EuroQOL-5L, mean (SD) |  |  |  |
| Health utility score (-0.68 to 1) | 6 months minus 12 week | 0.02(-0.03 to 0.07); 248^‡^ | 0.49 |

Unless otherwise indicated analyses were conducted with linear regression models with baseline scores entered as covariates. Due to skewed distributions, the change score between timepoints was used for all outcomes. Confidence intervals have not been adjusted for multiplicity, so inferences drawn from the intervals may not be reproducible. Between-group differences are presented as odds ratios; † a negative coefficient favours the intervention group; significant interactions (p≤ 0.05) for pre-specified variables at these timepoints: ^#^ age as a continuous variable; ^*^ age dichotomised at the median (76 years); ^&^baseline mobility as a continuous variable (Short Physical Performance Battery total score); ^^^ prior device use; ^§^ state (New South Wales vs. South Australia); ^¶^ health condition (neurological vs. non-neurological); ^‡^ sex; ~Analyses conducted with ordered logistic regression for final scores with baseline scores as a covariate.
