# Supplementary material for: Digitally enabled aged care and neurological rehabilitation to enhance outcomes with Activity and MObility UsiNg Technology (AMOUNT) in Australia: A randomised controlled trial
Source: PLoS Med. 2020 Feb 18;17(2):e1003029. doi: 10.1371/journal.pmed.1003029 (PMC7028259; doi:10.1371/journal.pmed.1003029)
Supplement: S5 Table — (DOCX) [file pmed.1003029.s006.docx]

| S5 Table. Fall Outcomes at 26 weeks | | |
| --- | --- | --- |
|  | **Intervention** | **Control** |
| Number of participants | 145 | 144 |
| Number of falls | 117 | 97 |
| Participants with at least 1 fall, n (%) | 53 (37) | 53 (37) |
| Participants with 2 or more falls, n (%) | 26 (18) | 21 (15) |
| Participants with 3 or more falls, (n (%) | 15 (10) | 12 (8) |
| Participants with 4 or more falls, n (%) | 9 (6) | 6 (4) |
| Number of injurious falls^#^ | 27 | 30 |
| Participants with injurious falls^#^, n (%) | 21 (14) | 19 (13) |
| Mean (SD) surveillance period (weeks) | 23.3 (6.0) | 23.4 (6.2) |
| Unadjusted IRR (95% CI), p | 1.19 (0.78 to 1.83), p = 0.43 | |

Fall rates between groups were compared using negative binomial regression.
^#^ Injurious falls include cuts/grazes, dislocation, sprains, traumatic brain injury, fractures; IRR: Incidence rate ratio.
