# Supplementary material for: Digitally enabled aged care and neurological rehabilitation to enhance outcomes with Activity and MObility UsiNg Technology (AMOUNT) in Australia: A randomised controlled trial
Source: PLoS Med. 2020 Feb 18;17(2):e1003029. doi: 10.1371/journal.pmed.1003029 (PMC7028259; doi:10.1371/journal.pmed.1003029)
Supplement: S6 Table — (DOCX) [file pmed.1003029.s007.docx]

| S6 Table. Interaction p-values for co-primary outcomes and mean between group difference (MD) (95% CI) for significant (p≤ 0.05) interaction terms | | | |
| --- | --- | --- | --- |
| **Outcome** | **Time points** | | |
| **Mobility** (+ve MD favours intervention group) | **3 week minus baseline** | **6 months minus baseline** | **6 months minus 3 week** |
| Short Physical Performance Battery (continuous version, 0-3) | |  |  |
| Health condition | 0.18 | 0.13 | 0.62 |
| Sex | 0.19 | 0.15 | 0.87 |
| Age | 0.06 | 0.07 | 0.40 |
| Mobility | <0.01^&^; 0.06^+^  Short Physical Performance Battery <4: 0.5 (0.2 to 0.7); 115; p<0.01  Short Physical Performance Battery ≥4: 0.1 (0.0 to 0.3); 164; p=0.10 | 0.02^&^; 0.48^+^  Short Physical Performance Battery <4: 0.3 (0.1 to 0.6); 105; p=0.02  Short Physical Performance Battery ≥4: 0.1 (0.0 to 0.3); 149; p=0.12 | 0.45 |
| Prior device use | 0.80 | 0.76 | 0.61 |
| State | 0.27 | < 0 01  SA: 0.0 (-0.1 to 0.2); 156; p=0.66  NSW: 0.5 (0.2 to 0.7); 98; p<0.01 | <0.01  SA: -0.1 (-0.3 to -0.0); 155; p=0.14  NSW: 0.2 (0.0 to 0.4); 94; p=0.03 |
| **Physical Activity** (+ve MD favours intervention group) | **3 week** | **6 months** |  |
| Proportion of the day spent upright, % | |  |  |
| Health condition | 0.71 | 0.83 |  |
| Sex | 0.35 | 0.68 |  |
| Age | 0.98 | 0.33 |  |
| Mobility | 0.41 | 0.10 |  |
| Prior device use | 0.09 | 0.06 |  |
| State | 0.47 | 0.03  SA: -2.2 (-5.3 to 0.9); 150; p=0.17  NSW: 3.4 (-0.9 to 7.6); 89; p=0.12 | |

^&^baseline mobility as a continuous variable (Short Physical Performance Battery total score); ^+^baseline mobility dichotomised at the median (Short Physical Performance Battery total score 4).
