# Supplementary material for: Digitally enabled aged care and neurological rehabilitation to enhance outcomes with Activity and MObility UsiNg Technology (AMOUNT) in Australia: A randomised controlled trial
Source: PLoS Med. 2020 Feb 18;17(2):e1003029. doi: 10.1371/journal.pmed.1003029 (PMC7028259; doi:10.1371/journal.pmed.1003029)
Supplement: S7 Table — (DOCX) [file pmed.1003029.s008.docx]

| S7 Table. Interaction p-values for secondary outcomes and mean between group difference (MD) (95% CI) for significant interaction terms | | | | | | |
| --- | --- | --- | --- | --- | --- | --- |
| **Outcomes** | **Timepoints** | | | | | |
| **Mobility** (+ve MD favours intervention group) | **3 week minus baseline** | | **6 months minus baseline** | | **6 months minus 3 week** | |
| Short Physical Performance Battery total score (0-12) | | |  | |  | |
| Health condition | 0.03  Neurological: 1.5 (0.7 to 2.2); 159; p<0.01  Not neurological: 0.2 (-0.7 to 1.0); 125; p=0.66 | | 0.05  Neurological: 1.4 (0.6 to 2.2); 149; p<0.01  Not neurological: 0.1 (-1.0 to 1.2); 108; p=0.88 | | 0.80 | |
| Sex | 0.08 | | 0.17 | | 0.87 | |
| Age | 0.14 | | 0.01^#,^*  ≥76: 0 (-0.9 to 0.9); 130; p=0.94  <76: 1.5 (0.5 to 2.5); 127; p<0.01 | | 0.04^#^; 0.12* ≥76: -0.5 (-1.2 to 0.3); 129; p=0.22  <76: 0.6 (-0.2 to 1.4); 126; p=0.15 | |
| Mobility | 0.03^&^; 0.33^+^  Short Physical Performance Battery <4:  1.3 (0.4 to 2.3); 120; p=0.01  Short Physical Performance Battery ≥4:  0.6 (-0.1 to 1.3); 164; p=0.12 | | 0.06 | | 0.45 | |
| Prior device use | 0.53 | | 0.29 | | 0.55 | |
| State | 0.68 | | 0.01  SA: 0.2 (-0.7 to 1.0); 158; p=0.69 NSW: 2.0 (0.9 to 3.1); 99; p<0.01 | | 0.01 SA: -0.5 (-1.2 to 0.2); 158; p=0.18 NSW: 1.1 (0.3 to 1.9); 97; p<0.01 | |
| de Morton Mobility Index (0-100) | | |  | |  | |
| Health condition | 0.90 | | 0.58 | | 0.81 | |
| Sex | 0.06 | | 0.02  Male: -1.9 (-8.0 to 4.1); 134; p=0.53  Female: 7.9 (2.6 to 13.2); 122; p<0.01 | | 0.17 | |
| Age | 0.76 | | 0.10 | | 0.08 | |
| Mobility | <0.01^&^; 0.29^+^  Short Physical Performance Battery <4:  7.6 (3.1 to 12.0); 120; p=<0.01  Short Physical Performance Battery ≥4:  1.7 (-2.6 to 6.0); 164; p=0.44 | | 0.30 | | 0.69 | |
| Prior device use | 0.95 | | 0.86 | | 0.93 | |
| State | 0.89 | | 0.17 | | 0.13 | |
| Single leg stance, (0-10 s) | | |  | |  | |
| Health condition | 0.43 | | 0.35 | | 0.64 | |
| Sex | 0.63 | | 0.52 | | 0.46 | |
| Age | 0.13 | | 0.33 | | 0.63 | |
| Mobility | 0.74 | | 0.40 | | 0.34 | |
| Prior device use | 0.28 | | 0.77 | | 0.62 | |
| State | 0.31 | | 0.02  SA: 0.3 (-0.9 to 1.6); 157; p=0.63 NSW: 2.6 (1.1 to 4.2); 99; p<0.01 | | <0.01  SA: -0.5 (-1.6 to 0.6); 157; p=0.36 NSW: 2.5 (1.1 to 3.9); 97; p<0.01 | |
| Maximal balance range test, mm | | |  | |  | |
| Health condition | 0.32 | | 0.44 | | 0.99 | |
| Sex | 0.27 | | 0.94 | | 0.43 | |
| Age | 0.33 | | 0.11 | | 0.34 | |
| Mobility | 0.01^&^; 0.04^+^  Short Physical Performance Battery <4:  21.9 (0.1 to 43.6); 120; p=0.05  Short Physical Performance Battery ≥4:  14.4 (-2.4 to 31.3); 164; p=0.09 | | 0.12 | | 0.95 | |
| Prior device use | 0.66 | | 0.54 | | 0.60 | |
| State | 0.40 | | 0.24 | | 0.42 | |
| Step test, steps (average of both legs) | | |  | |  | |
| Health condition | 0.01  Neurological: 2.8 (1.3 to 4.3); 159; p<0.01  Not neurological: -0.2 (-1.6 to 1.2); 125; p=0.82 | | 0.16 | | 0.48 | |
| Sex | 0.47 | | 0.24 | | 0.47 | |
| Age | 0.08 | | 0.06 | | 0.32 | |
| Mobility | 0.21 | | 0.10 | | 0.34 | |
| Prior device use | 0.37 | | 0.28 | | 0.80 | |
| State | 0.05  SA: 2.5 (1.1 to 4.0); 172; p<0.01 NSW: 0.4 (-1.1 to 1.9); 112; p=0.60 | | 0.52 | | 0.02  SA: -0.4 (-1.8 to 0.9); 158; p=0.55 NSW: 1.9 (0.7 to 3.1); 97; p<0.01 | |
| **Physical Activity** (+ve MD favours intervention group) | **3 week minus baseline** | | **6 months minus baseline** | | **6 months minus 3 week** | |
| Time spent standing, min/day | | |  | |  | |
| Health condition | 0.66 | | 0.74 | | 0.36 | |
| Sex | 0.49 | | 0.78 | | 0.90 | |
| Age | 1.0 | | 0.57 | | 0.86 | |
| Mobility | 0.62 | | 0.18 | | 0.47 | |
| Prior device use | 0.14 | | 0.04  Prior device use: -34.0 (-68.6 to 0.6); 149; p=0.05  No prior device use: 32.3 (-25.7 to 90.2); 90; p=0.27 | | 0.06 | |
| State | 0.59 | | 0.07 | | 0.17 | |
| Time spent stepping, min/day | | |  | |  | |
| Health condition | 0.76 | | 0.72 | | 0.56 | |
| Sex | 0.44 | | 0.64 | | 0.86 | |
| Age | 0.74 | | 0.05  ≥76yr: -3.2 (-14.4 to 7.9); 121; p= 0.57  <76yr: 14.4 (-1.2 to 29.9); 118; p=0.07 | | 0.04  ≥76yr: -4.7 (-12.7 to 3.2); 120; p= 0.24  <76yr: 13.5 (0.7 to 26.4); 114; p=0.04 | |
| Mobility | 0.35 | | 0.09 | | 0.38 | |
| Prior device use | 0.06 | | 0.48 | | 0.97 | |
| State | 0.14 | | 0.01  SA: -3.4 (-15.1 to 8.3); 150; p=0.56 NSW: 21.9 (4.8 to 38.9); 89; p=0.01 | | 0.05  SA: -0.8 (-9.8 to 8.1); 146; p=0.85 NSW: 15.3 (1.4 to 29.1); 88; p=0.03 | |
| Number of steps per day |  | |  | |  | |
| Health condition | 0.59 | | 0.57 | | 0.78 | |
| Sex | 0.58 | | 0.43 | | 0.79 | |
| Age | 0.52 | | 0.03^#^; 0.06*  ≥76yr: -118 (-964 to 728); 121; p= 0.78  <76yr: 1219 (2 to 2436); 118; p=0.05 | | 0.02^#^; 0.01* ≥76yr: -351 (-951 to 249); 120; p= 0.25  <76yr: 1173 (165 to 2180); 114; p=0.02 | |
| Mobility | 0.44 | | 0.12 | | 0.46 | |
| Prior device use | 0.04  Prior device use: -111 (-741 to 519); 164; p=0.73  No prior device use: 852 (206 to 1499); 107; p=0.01 | | 0.48 | | 0.91 | |
| State | 0.13 | | 0.02 SA: -86 (-1004 to 831); 150; p=0.85 NSW: 1742 (436 to 3049); 89; p=0.01 | | 0.06 | |
| Number of sit to stands per day | | |  | |  | |
| Health condition | 0.63 | | 0.06 | | 0.09 | |
| Sex | 0.70 | | 0.81 | | 0.90 | |
| Age | 0.28 | | 0.18 | | 0.32 | |
| Mobility | 0.91 | | 0.46 | | 0.65 | |
| Prior device use | 0.79 | | 0.86 | | 0.90 | |
| State | 0.44 | | 0.01  SA: -2 (-6 to 3); 150; p=0.44 NSW: 9 (2 to 15); 89; p=0.01 | | 0.02  SA: -1 (-5 to 3); 146; p=0.58 NSW: 7 (2 to 13); 88; p=0.01 | |
| **Cognition** (-ve MD favours intervention group) | **3 week minus baseline** | | **6 months minus baseline** | | **6 months minus 3 week** | |
| Trail Making Test A, s |  | |  | |  | |
| Health condition | 0.26 | | 0.17 | | 0.47 | |
| Sex | 0.01  Male: -0.3 (-6.5 to 6.0); 145; p=0.93  Female: -10.3 (-16.0 to -4.5); 138; p<0.01 | | 0.04  Male: 4.0 (-3.8 to 11.8); 134; p=0.32  Female: -7.2 (-14.3 to -0.1); 121; p<0.05 | | 0.82 | |
| Age | 0.61 | | 0.96 | | 0.64 | |
| Mobility | 0.08 | | 0.21 | | 0.93 | |
| Prior device use | 0.03  Prior device use: -1.5 (-6.7 to 3.7); 174; p=0.58  No prior device use: -10.6 (-18.0 to -3.2); 109; p=0.01 | | 0.12 | | 0.42 | |
| State | 0.43 | | 0.39 | | 0.33 | |
| Trail Making Test B, s |  | |  | |  | |
| Health condition | 0.80 | | 0.36 | | 0.47 | |
| Sex | 0.02  Male: 15.2 (-1.6 to 32.0); 145; p=0.08  Female: -15.0 (-35.4 to 5.4); 138; p=0.15 | | 0.57 | | 0.12 | |
| Age | 0.22 | | 0.24 | | 0.65 | |
| Mobility | 0.28 | | 0.55 | | 0.76 | |
| Prior device use | 0.34 | | 0.81 | | 0.56 | |
| State | 0.52 | | 0.76 | | 0.61 | |
| Trail Making Test B – A, s |  | |  | |  | |
| Health condition | 0.33 | | 0.04  Non-Neurological: 13.0 (-4.0 to 29.9); 106; p=0.13  Neurological: -8.9 (-21.7 to 3.8); 148; p=0.17 | | 0.04  Non-Neurological: 11.4 (-3.4 to 26.3); 104; p=0.13  Neurological: -8.0 (-19.7 to 3.7); 147; p=0.18 | |
| Sex | 0.04  Male: 12.6 (-0.4 to 25.6); 145; p=0.06  Female: -9.5 (-25.7 to 6.7); 138; p=0.25 | | 0.96 | | 0.29 | |
| Age | 0.22 | | 0.43 | | 0.75 | |
| Mobility | 0.99 | | 0.93 | | 0.73 | |
| Prior device use | 0.38 | | 0.90 | | 0.83 | |
| State | 0.32 | | 0.87 | | 0.63 | |
| **Participant Recorded Outcome Measures** | |  | |  | |  |
| Incidental and Planned Exercise Questionnaire (+ve MD favours intervention group) | 12 week minus 3 week | 6 months minus 3 week | | 6 months minus 12 week | |  |
| Total score, h/wk |  |  | |  | |  |
| Health condition | 0.42 | 0.92 | | 0.42 | |  |
| Sex | 0.57 | 0.36 | | 0.90 | |  |
| Age | 0.40 | 0.24 | | 0.14 | |  |
| Mobility | 0.11 | 0.08 | | 0.28 | |  |
| Prior device use | 0.95 | 0.36 | | 0.35 | |  |
| State | 0.91 | 0.40 | | 0.75 | |  |
| Home exercise subscale score, h/wk | |  | |  | |  |
| Health condition | 0.36 | 0.97 | | 0.94 | |  |
| Sex | 0.95 | 0.79 | | 0.94 | |  |
| Age | 0.58 | 0.01^#,^ 0.27^*^  ≥76yr: 0.9 (-0.0 to 1.9); 128; p=0.06  <76yr: 0.3 (-0.7 to 1.2); 126; p=0.61 | | 0.02^#^ 0.42^*^  ≥76yr: 0.8 (-0.1 to 1.7); 124; p=0.09  <76yr: 0.2 (-0.9 to 1.3); 117; p=0.73 | |  |
| Mobility | 0.88 | 0.82 | | 0.98 | |  |
| Prior device use | 0.40 | 0.36 | | 0.31 | |  |
| State | 0.97 | 0.16 | | 0.18 | |  |
| Walk subscale score, h/wk | |  | |  | |  |
| Health condition | 0.11 | 0.77 | | 0.18 | |  |
| Sex | 0.16 | 0.28 | | 0.84 | |  |
| Age | 0.39 | 0.08 | | 0.15 | |  |
| Mobility | 0.04^&^; 0.37+  Short Physical Performance Battery <4: 1.7 (0.8 to 2.6); 106; p=<0.01  Short Physical Performance Battery ≥4: -0.0 (-1.6 to 1.5); 146; p=0.98 | 0.97 | | 0.29 | |  |
| Prior device use | 0.82 | 1.00 | | 0.72 | |  |
| State | 0.17 | 0.81 | | 0.24 | |  |
| Modified Computer Self-Efficacy Scale (10-100) (+ve MD favours intervention group) | 3 week minus baseline | 12 week minus baseline | | 6 months minus baseline | | 6 months minus 12 week |
| Health condition | 0.95 | 0.97 | | 0.50 | | 0.42 |
| Sex | 0.61 | 0.97 | | 0.04  Male:-3.3 (-10.2 to 3.6); 134; p=0.35  Female: 8.3 (-0.4 to 17.0); 122; p=0.06 | | 0.05  Male:-2.3 (-8.6 to 3.9); 128; p=0.46  Female: 7.6 (0.0 to 15.2); 119; p=0.05 |
| Age | 0.46 | 0.77 | | 0.84 | | 0.72 |
| Mobility | 0.69 | 0.48 | | >0.05 | | 0.11 |
| Prior device use | 0.46 | 0.71 | | 0.46 | | 0.45 |
| State | 0.67 | 0.65 | | 0.35 | | 0.18 |
| Activities-specific Balance Confidence Scale (0-100) (+ve MD favours intervention group) | 3 week minus baseline | 12 week minus baseline | | 6 months minus baseline | | 6 months minus 12 week |
| Health condition | 0.97 | 0.13 | | 0.75 | | 0.33 |
| Sex | 0.10 | 0.45 | | 0.07 | | 0.24 |
| Age | 0.66 | 0.08 | | 0.15 | | 0.70 |
| Mobility | 0.19 | 0.06 | | 0.06 | | 0.48 |
| Prior device use | 0.82 | 0.30 | | 0.85 | | 0.63 |
| State | 0.58 | 0.01  SA: -5.1 (-12.7 to 2.4); 160; p=0.18  NSW:11.7 (0.8 to 22.6); 100; p=0.04 | | 0.06 | | 0.97 |
| WHO Disability Assessment Schedule 2.0 (raw score 12-60) (-ve MD favours intervention group) | 12 week minus 3 week | 6 months minus 3 week | | 6 months minus 12 week | |  |
| Health condition | 0.23 | 0.35 | | 0.86 | |  |
| Sex | 0.30 | 0.02  Male:1.3 (-1.2 to 3.8); 132; p=0.30  Female: -2.9 (-5.4 to -0.3); 123; p=0.03 | | <0.01  Male: 1.7 (-0.4 to 3.8); 129; p=0.10  Female: -3.4 (-5.5 to -1.4); 120; p<0.01 | |  |
| Age | 0.02^#^ 0.03*  ≥76yr: 1.8 (-0.7 to 4.3); 133; p=0.15  <76yr: -2.5 (-5.7 to 0.7); 128; p= 0.13 | 0.07 | | 0.42 | |  |
| Mobility | 0.09 | 0.71 | | 0.66 | |  |
| Prior device use | <0.01  No: 3.2 (-0.1 to 6.6); 100; p=0.06  Yes:-2.4 (-4.9 to 0.2); 161; p=0.07 | 0.36 | | 0.46 | |  |
| State | 0.01  SA: 1.9 (-0.4 to 4.3); 161; p=0.10  NSW:-3.2 (-6.9 to 0.5); 100; p=0.09 | 0.02  SA: 0.9 (-1.1 to 2.8); 158; p=0.37  NSW: -3.0 (-6.3 to 0.4); 97; p=0.09 | | 0.54 | |  |
| Short Form 6-Dimensions questionnaire health utility score (0-1) (+ve MD favours intervention group) |  | 3 week | | 12 week | | 6 months |
| Health condition |  | 0.68 | | 0.54 | | 0.35 |
| Sex |  | 0.13 | | 1.00 | | 0.01  Male:-0.08 (-0.18 to 0.01); 135; p=0.09  Female: 0.09 (0.01 to 0.18); 121; p=0.04 |
| Age |  | 0.21 | | 0.06 | | 0.41 |
| Mobility |  | 0.86 | | 0.17 | | 0.82 |
| Prior device use |  | 0.09 | | 0.15 | | 0.06 |
| State |  | 0.13 | | 0.04  SA: -0.04 (-0.11 to 0.03); 160; p=0.29  NSW: 0.10 (-0.02 to 0.22); 100; p=0.09 | | 0.01  SA: -0.06 (-0.12 to 0.01); 157; p=0.11  NSW:0.10 (-0.02 to 0.23); 99; p=0.11 |
| EuroQOL-5L health utility score (-0.68 to 1) (+ve MD favours intervention group) | 3 week minus baseline | 12 week minus baseline | | 6 months minus baseline | | 6 months minus 12 week |
| Health condition | 0.75 | 0.76 | | 0.94 | | 0.78 |
| Sex | 0.49 | 0.66 | | 0.02  Male: -0.03 (-0.12 to 0.07); 135; p=0.58  Female: 0.12 (0.04 to 0.21); 123; p<0.01 | | 0.05  Male: -.03 (-0.11 to 0.04); 129; p=0.41  Female: 0.08 (0.00 to 0.15); 119; p=0.05 |
| Age | 0.87 | 0.03^#^ 0.04^*^  ≥76yr: -0.02 (-0.11 to 0.08); 134; p=0.76  <76yr: 0.14 (0.03 to 0.25); 127; p= 0.02 | | 0.06 | | 0.25 |
| Mobility | 0.17 | 0.61 | | 0.75 | | 0.83 |
| Prior device use | 0.65 | 0.08 | | 0.57 | | 0.64 |
| State | 0.15 | 0.06 | | 0.18 | | 0.99 |
| Self-reported Falls | |  | | over 6 months | |  |
| Health condition |  |  | | 0.73 | |  |
| Sex |  |  | | 0.80 | |  |
| Age |  |  | | 0.33 | |  |
| Mobility |  |  | | 0.06 | |  |
| Prior device use |  |  | | 0.59 | |  |
| State |  |  | | 0.25 | |  |

^#^p-value for continuous age interaction variable; ^*^p-value for dichotomous age interaction variable dichotomised at the median value (76 years); ^&^baseline mobility as a continuous variable (Short Physical Performance Battery total score); ^+^baseline mobility dichotomised at the median (Short Physical Performance Battery total score 4); SA: South Australia; NSW: New South Wales.
