# Supplementary material for: Digitally enabled aged care and neurological rehabilitation to enhance outcomes with Activity and MObility UsiNg Technology (AMOUNT) in Australia: A randomised controlled trial
Source: PLoS Med. 2020 Feb 18;17(2):e1003029. doi: 10.1371/journal.pmed.1003029 (PMC7028259; doi:10.1371/journal.pmed.1003029)
Supplement: S1 Text — (DOCX) [file pmed.1003029.s009.docx]

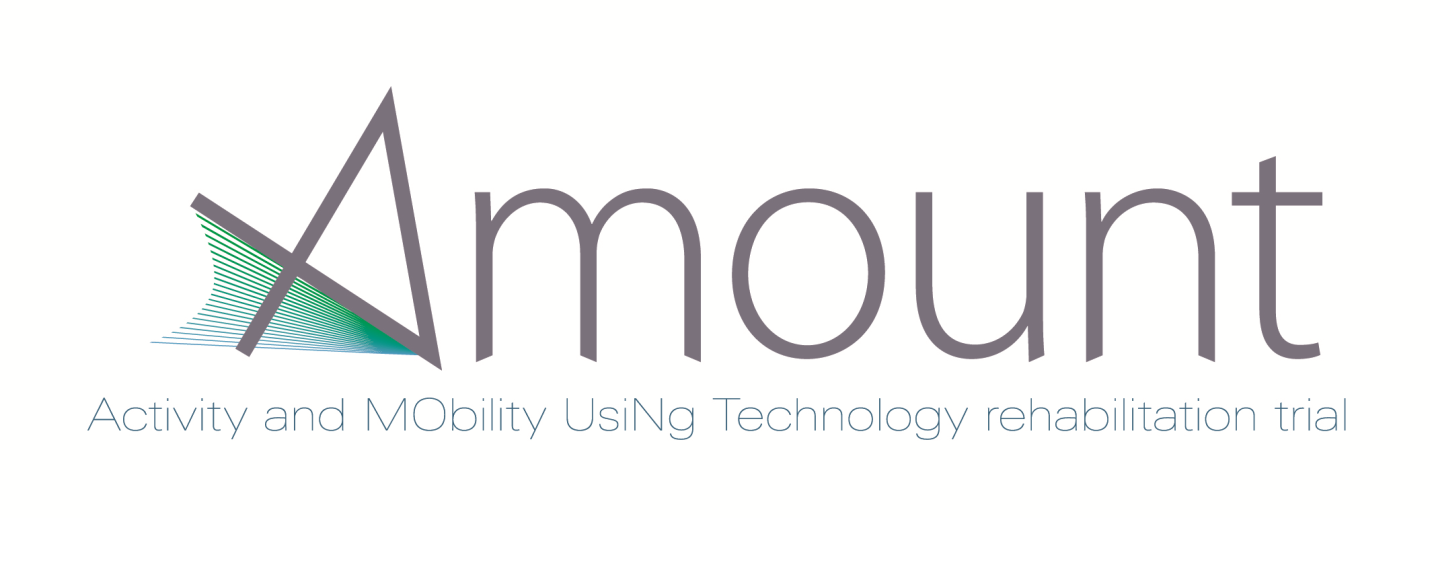


**INTERVENTION**


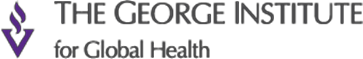
**PROTOCOL**


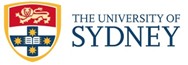


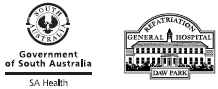

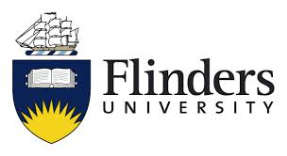

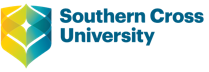

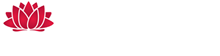

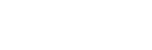


Contents

[INTERVENTION PROTOCOL 6](#_Toc498696222)

[Aim 6](#_Toc498696223)

[Setting 6](#_Toc498696224)

[Length of Program 6](#_Toc498696225)

[Provider of intervention 6](#_Toc498696226)

[Frequency 6](#_Toc498696227)

[Duration 7](#_Toc498696228)

[Intensity 7](#_Toc498696229)

[Type 7](#_Toc498696230)

[Supervision and safety 7](#_Toc498696231)

[Progression 8](#_Toc498696232)

[Feedback 8](#_Toc498696233)

[Goal setting 9](#_Toc498696234)

[Global goals and sub-goals 10](#_Toc498696235)

[Goal rating 10](#_Toc498696236)

[Goal example 12](#_Toc498696237)

[AMOUNT REHABILITATION TRIAL: FLOW DIAGRAM OF INTERVENTION 13](#_Toc498696238)

[MOBILITY LIMITATIONS TABLES 14](#_Toc498696239)

[Standing Up from a chair 14](#_Toc498696240)

[Maintaining a standing position 16](#_Toc498696241)

[Reaching while Standing 17](#_Toc498696242)

[Stepping while standing 18](#_Toc498696243)

[Changing directions while walking 20](#_Toc498696244)

[Stairclimbing 21](#_Toc498696245)

[Physical activity throughout day 22](#_Toc498696246)

[DEVICES 23](#_Toc498696247)

[NINTENDO WII 23](#_Toc498696248)

[Getting started with Wii 23](#_Toc498696249)

[Creating a new Mii 23](#_Toc498696250)

[Getting started with WiiFit 24](#_Toc498696251)

[Game selection 24](#_Toc498696252)

[Getting started with Wii Family Trainer 24](#_Toc498696253)

[Other helpful hints for using the Wii 25](#_Toc498696254)

[WII FIT 26](#_Toc498696255)

[WII FAMILY TRAINER 30](#_Toc498696256)

[NINTENDO WII U 33](#_Toc498696257)

[Setting up the Wii U console and GamePad 33](#_Toc498696258)

[Introduction to Homepage on GamePad and TV screen 33](#_Toc498696259)

[Getting started with WiiFit Plus U 33](#_Toc498696260)

[Creating a Mii Character 34](#_Toc498696261)

[Navigating through main menu 35](#_Toc498696262)

[Navigating through training menu 36](#_Toc498696263)

[How to Pair a Wii Remote with the Console 37](#_Toc498696264)

[What to do if having difficulty setting up internet 37](#_Toc498696265)

[Wii Motion Plus 38](#_Toc498696266)

[Other helpful hints for using the Wii U 39](#_Toc498696267)

[WII U FIT 40](#_Toc498696268)

[XBOX KINECT 43](#_Toc498696269)

[Getting started with Xbox Kinect 43](#_Toc498696270)

[Creating an avatar in Xbox Kinect 43](#_Toc498696271)

[Playing a game with Xbox Kinect 43](#_Toc498696272)

[KINECT ADVENTURES 45](#_Toc498696273)

[KINECT YOUR SHAPE FITNESS EVOLVE 2012 46](#_Toc498696274)

[KINECT SPORTS 48](#_Toc498696275)

[KINECT DOWNLOADED GAMES 48](#_Toc498696276)

[HUMAC 2013 (v.150) 49](#_Toc498696277)

[Getting started with HUMAC 49](#_Toc498696278)

[Adding a new participant into the HUMAC 49](#_Toc498696279)

[Commence exercising or testing 49](#_Toc498696280)

[Other features 50](#_Toc498696281)

[Participant setup on the board 50](#_Toc498696282)

[HUMAC 51](#_Toc498696283)

[HUMAC GAMES 57](#_Toc498696284)

[FYSIOGAMING 2015 (v2.1) 61](#_Toc498696285)

[Set-up 61](#_Toc498696286)

[Adding a new participant 61](#_Toc498696287)

[Patient Tab 61](#_Toc498696288)

[Starting to exercise 62](#_Toc498696289)

[Difficulty level 62](#_Toc498696290)

[Assessment Centre 62](#_Toc498696291)

[Reviewing and reporting participant data 62](#_Toc498696292)

[FYSIOGAMING 64](#_Toc498696293)

[STEPPING TILES 68](#_Toc498696294)

[Equipment 68](#_Toc498696295)

[Getting started 68](#_Toc498696296)

[Overview of the screen 68](#_Toc498696297)

[Using the system with only the maintile 68](#_Toc498696298)

[Using the system with the maintile, bridging and subtiles 69](#_Toc498696299)

[Other tips for using the system 69](#_Toc498696300)

[STEPPING TILES 70](#_Toc498696301)

[AMOUNT APP 76](#_Toc498696302)

[Getting started 76](#_Toc498696303)

[Using the AMOUNT App 76](#_Toc498696304)

[Working in Administrator mode 76](#_Toc498696305)

[Working in therapist mode 77](#_Toc498696306)

[Working in Patient mode 78](#_Toc498696307)

[Other information 79](#_Toc498696308)

[iPad AMOUNT APP 80](#_Toc498696309)

[T-REX APP 84](#_Toc498696310)

[iPad T-REX APP 86](#_Toc498696311)

[FITBIT 91](#_Toc498696312)

[Installing the Fitbit on your computer 91](#_Toc498696313)

[To pair your Fitbit device with the online Fitbit account 91](#_Toc498696314)

[Wearing the Fitbit 91](#_Toc498696315)

[To sync your device 91](#_Toc498696316)

[Accessing your information 91](#_Toc498696317)

[Fitbit One 92](#_Toc498696318)

[Fit Bit Zip 92](#_Toc498696319)

[Fit Bit Charge 92](#_Toc498696320)

[Fit Bit App 93](#_Toc498696321)

[Feedback graph 94](#_Toc498696322)

[Example of pedometer log sheet 95](#_Toc498696323)

[Garmin Watches (vivofit) 96](#_Toc498696324)

[Installing the Garmin vivofit on your computer or mobile device 96](#_Toc498696325)

[Accessing your information 96](#_Toc498696326)

[Device information 96](#_Toc498696327)

[PHONE APPS 97](#_Toc498696328)

[Runkeeper 97](#_Toc498696329)

[Walk Forward 98](#_Toc498696330)

[1. Overview of Walk Forward 98](#_Toc498696331)

[2. App Installation, Versions and Operating Systems 99](#_Toc498696332)

[3. Using the App 100](#_Toc498696333)

[INTERVENTION PLANNING FORM: INPATIENT 104](#_Toc498696334)

[INPATIENT PRACTICE SHEET 105](#_Toc498696335)

[COMMUNITY INTERVENTION COVER SHEET 107](#_Toc498696336)

[INTERVENTION PLANNING FORM: COMMUNITY 110](#_Toc498696337)

[INITIAL HEALTH COACHING SESSION 111](#_Toc498696338)

[INTERVENTION TECHNOLOGY SUPPORT 112](#_Toc498696339)

[COMMUNITY GOAL SETTING 113](#_Toc498696340)

*Images and screenshots removed from document for publication.

# INTERVENTION PROTOCOL

## Aim

To increase mobility and physical activity through the addition of tailored prescription of affordable technologies in addition to usual care for people admitted to aged care and neurological rehabilitation wards with mobility limitations.

## Setting

The inpatient intervention will be conducted within the physiotherapy rehabilitation gyms of the site hospitals. The community-based program will commence once discharged from a rehabilitation ward and can include home, transitional living ward or residential care settings.

## Length of Program

Inpatient program until discharge; up to 6 months after randomisation for community program.

## Provider of intervention

The research physiotherapist will determine the most appropriate intervention based on discussion with the clinical physiotherapist, baseline assessment, participant goals, and the technology suitable in accordance with the intervention protocol. The research physiotherapist will provide the inpatient intervention one-on-one for the length of their inpatient rehabilitation, and then support the community program by using phone, email, or video conference contact once they are home. A home visit may be required to set up the technology prior to discharge, and up to 5 home visits may be provided during the program if there are problems with the technologies or if technologies/program needs upgrading.

## Frequency

Participants should participate in technology-based intervention ≥5 days per week for the length of the program. The research physiotherapist will provide one-on-one intervention 5x per week during the inpatient stage. The research physiotherapist will provide support and health coaching as required by each participant during the community stage. It is recommended support is provided weekly to begin with, but the frequency can reduce if the participant is managing their program well.

## Duration

30-60 minutes with supervision (+ any additional unsupervised activity if the therapist deems them safe) as an inpatient. 30-60 minutes daily as independent practice in community setting.

## Intensity

Nil specified, as per participant ability.

## Type

The prescription of technology to target mobility and physical activity problems will include, but will not be limited to, the use of video, computer and tablet programs and applications to encourage structured exercise and other forms of physical activity as well as pedometers to provide feedback on activity levels. The chosen technologies are all relatively low cost and will provide feedback on mobility task or physical activity performance or dose. The research physiotherapist will choose the most appropriate technology/ies for an individual participant by following this protocol which has been refined through pilot testing. The research protocol includes tables to guide the choice of exercises/games and technology based on mobility limitations, and tables detailing all the different games/exercises within each device and how to utilise them for various mobility limitations. These tables will be reviewed quarterly and any new technologies will be added throughout the trial.

Each technology to be used will 1) provide feedback about task performance; 2) facilitate individualised tailoring and progression of exercise or physical activity; 3) enable progress towards a functionally relevant goal(s) to be recorded and reviewed; and 4) is relatively inexpensive.

## Supervision and safety

The safety of the participants is of the upmost importance. Exercises should be challenging (e.g. no hand support where possible), but the environment or supervision used to maximise safety. For example, the participant can be set up next to a wall with a table or chair on their other side to increase safety while practicing standing activities. The research physiotherapist will provide one-on-one supervision initially to ensure safety and to help participants to use the technologies outside of therapy times. Participants will be encouraged to exercise unsupervised or with family/friends once they are safe to do so, however the research physiotherapist will still oversee the sessions to ensure the prescribed dose is delivered. Once in the community, the research physiotherapist will instruct the participant on how to safely exercise with the supervision of family or friends and/or independently.

## Progression

- Reduce length/frequency of rest period between exercises
- Reduce *sitting* rest periods (i.e. stay standing between exercises)
- Increase difficulty of mobility task
- Increase difficulty of chosen technology (e.g. games/exercises that require timing, decisions, UL use)
- Progress type of mobility task (e.g. from standing to stepping exercises)
- Increase participant independence with exercises and use of technology

## Feedback

As stated above, each device must be able to provide feedback about task performance. Feedback can be provided as Knowledge of Results (KOR) or Knowledge of Performance (KOP).

KOR is a form of augmented feedback where verbal, or visual information is given to a participant at the end of the performance of a skill; the feedback is about the outcome of the performance, rather than about the movements which brought about the performance. KOP is a form of augmented feedback given verbally or visually either during or after the performance of a skill.

The feedback contains information about the nature of the movement pattern produced during the performance and may include identification of the parts of the skill which were performed correctly and the parts performed incorrectly.

KOR is provided visually from the devices such as the score in completing a game or exercise (Wii, Xbox, Humac games, Fysiogaming) or the distance walked (phone app) or steps taken (Fitbit), or the number of repetitions achieved (iPad apps). Some devices also provide KOP visually such as the location of centre of pressure or centre of mass (e.g. ski slalom exercise on the Wii, Humac exercises, Stepping Tiles).

The Research Physiotherapist can also provide feedback verbally in the form of KOP (e.g. kinematics of performing an activity such as standing up) or KOR (e.g. number of repetitions, duration of an exercise).

Many of the devices also provide the option for KOP or KOR to be graphed or displayed in a table which can be provided to the participant (see example below of table of exercise session from Fysiogaming-KOR and graph of centre of pressure movement from the Humac-KOP).

The Research Physiotherapist can also create their own graphs for the patient (see example of step count graph from pedometer created in excel on page 99).

## Goal setting

Goal setting is a critical element of the intervention protocol. One of the features of the technologies used in the trial is that they enable progress towards a functionally relevant goal which is to be recorded, reviewed and can be graphed. Goals play a number of important roles in rehabilitation. For our participants, they can provide an agreement and structure of what they are going to work towards, and motivation to strive for achievement. By the use of linking sub-goals to global goals, they can also help the participant understand how the exercises/technologies are helping them work towards their more meaningful participation goal. For staff, goals can provide a framework and timeline for the intervention provided, and to ensure that they progress the participant when appropriate throughout the intervention. They may also provide valuable process information to help describe the intervention we delivered and the success of the participants within the intervention.

Goal setting is a skill that develops with practice. There are a few key elements to writing a good goal and can be remembered by the term “SMART” goals. That is

**S** specific

**M** measurable

**A** attainable- yet still challenging

**R** relevant to stakeholders – the participant, physiotherapist, family etc.

**T** time-bound

- Specific: The goal must be based around a specific observable behaviour or activity. For example, standing up, walking, stepping, reaching, boarding a bus, grocery shopping. Other elements that can make your goal very specific include defining the conditions that are required for performing the goal i.e. how will the client perform the task – with use of equipment, assistance, independently, verbal cues, supervision; and defining where the goal activity will be performed i.e. location, environment. For example: Jack will walk independently from his home to the local shops using a walking stick.
- Measurable: Need to be able to objectively measure whether the goal is achieved. May include 2 criteria: how achievement will be measured (e.g. test or piece of technology/game) and criteria for acceptable standard of outcome performance (e.g. 90% accuracy, < 5 mins, 8 out of 10 repetitions). (Mogensen 2008). For example, at least 6000 steps 3 days per week using the Fitbit.
- Attainable: Goals should be realistic, yet challenging. Research in people who have had a brain injury tell us that specific difficult goals results in better immediate performance on motor and cognitive tasks than “do your best” type of goals (Levack, 2006). It can be challenging in itself to set challenging goals, as a rule of thumb, Short Term Goals are “probably attainable” and Long-Term Goals are “possibly attainable” (Playford 2009).
- Relevant: Goals should be relevant to all stakeholders, in this instance; the stakeholders are the participants, perhaps family/friends of the participant, and us as staff on the trial. For the participant, this means involving them in the goal setting process and understanding what types of activities/roles they want to return to. For us, this means incorporating goals that work in some way towards improving mobility and physical activity. Non-collaborative goal setting (that is, the therapist independently determining all the goals without any discussion with the patient has been cited as one of the reasons for failure in neurorehabilitation (van der Broek 2005). Collaborative goal setting can be challenging with some people who may have unrealistic goals or who may have never set a goal before in their life. How involved the participant is and how important goal setting is to motivate the participant will probably vary considerably between participants.
- Timebound: The goal should incorporate a timeframe that you expect the goal to be achieved in. Not all goals have to be set for the same period of time; you just need to be able to review each goal within the timeframe for which it was set. If a goal is not achieved in that time, it should be rated as to how much achievement has been attained in that timeframe. No goal should be rated as ongoing, a new goal should be written.

### Global goals and sub-goals

One way to incorporate relevant goals for someone early in the intervention phase that still needs to do lots of practice at the activity or impairment level is to set an overarching global goal at the participation level. This is a goal that the patient is aiming to achieve at some time in the future. It may be getting back to living in their home independently, or being able to walk to the local shops to do their groceries, or go to the park with their grandchildren. This type of goal is a long term goal and is likely to take more than 6 weeks to achieve (perhaps even the whole of the intervention phase). You can then set smaller sub-goals under this larger goal that will help the participant work towards their more meaningful longer term goal. Below is an example of global goals with sub-goals. In some cases intervention may be focused on improving a large task that may take a significant amount of time. In these cases global goals may be written, with appropriate sub goals that break this large task down into more achievable steps. The timeframe for this global goal may be for a number of months with the sub-goals underneath changing as each are achieved and the client moves closer to achieving their global goal.

### Goal rating

A 5-point rating scale for the achievement of goals will be used to rate the achievement of goals by participants in the intervention group. The five rating choices are:

1. Not achieved
2. Partially achieved
3. Mostly achieved
4. Achieved
5. Achieved +

**1. Not achieved:** The participant did not successfully achieve any component of the goal due to for example, the technology was not provided, they did not practice, they no longer agreed to the goal, they tried but no progress was achieved, the goal was set too difficult.

2. **Partially achieved** (1-49%): The goal was partially achieved/completed, but less than 50%. For example, if there is a qualitative and a quantitative component of the goal, the person may have achieved one component, but was nowhere near achieving the other component. Another example may be that they may have started achieving the goal, but dropped off and were not consistent. Another example may be a number of components may be included in a goal and some are achieved or completed, but less than 50% of them.

3. **Mostly achieved** (50-94%): The goal or plan has been mostly achieved but less than 95%. For example if there is a qualitative and a quantitative component of the goal, the person may have achieved one component, and almost achieved the other component. Another example may be where a quota or set number of sessions or exercises are set as the goal. They may have come close to achieving this goal, but did not quite do all that was required. For example they may have gone for a walk 2x a week where the goal was set at 3x a week. Another example may be a number of components may be included in a goal or plan, where more than 50% are achieved but less than 95%

4**. Achieved (95-105%):** The goal set is achieved.

5. **Achieved + (> 105%):** This is awarded when a participant well exceeds the expectation of the goal that was set. For example the participant is consistently completing a walking program 5x per week for 1 hour per day using Runkeeper, where the goal was only set for 3x per week for > 30 minutes. Another example for this rating may be that they achieve a goal much earlier than the timeframe that was set.

Next to each rating there will be room to make a comment about the participant’s progress of each goal. This allows you to provide quantitative or qualitative information about the progress of the participant for each goal.

### Goal example

An example of a global goal and sub-goals for the AMOUNT intervention:

Global goal:

To walk independently to and from Bankstown train station from your home (~1km away) at least once per week to access community group activities within 5 months.

Subgoals:

- To complete all your IPad AMOUNT walking program exercises at least 5 times per week for the next 4 weeks.
- To walk to the end of your street and back without stopping on at least 3 days of the week measuring your walk using Runkeeper for the next 2 weeks.
- To walk at least 6000 steps on at least 3 days of the week measured using your Fitbit for the next 2 weeks.
- To play the WiiFit jogging plus game at least 3 times a week and complete the track in < 8 minutes within 4 weeks.
- To achieve a score of > ........ on the Xbox Kinect “stomp it” game by stepping only on the purple and orange lights within 2 weeks.
- To complete 50 repetitions without a rest using the Humac left right weight shift game in < 5 minutes with a score > 80% within 1 week.
- To achieve a score of > ....... on the Fysiogaming sideways walking game (difficulty level 5) within 2 weeks.
- To complete 50 stepping exercises on the stepping tiles/IPad stepping exercise with your right foot without using your hands within 2 weeks

# AMOUNT REHABILITATION TRIAL: FLOW DIAGRAM OF INTERVENTION

Record intervention plans using **intervention planning sheet**

**INTERVENTION PLANNING**

Research PT to review initial PT assessment in notes. Discuss with clinical PT current impairments, mobility limitations, exercises, goals for therapy, and anticipated LOS. Use mobility limitation tables to guide technology choice

**GROUP ALLOCATION**

Randomised into experimental group

Ongoing usual care in am with clinical PT (record using **usual practice sheet**)

Record intervention using **community sheets**

Record discharge plans using **intervention planning sheet**

Record intervention using **inpatient practice sheet**

**COMMUNITY INTERVENTION**

weekly to fortnightly phone, email, SMS, home visit, video conference contact incorporating health coaching to support technology use to increase physical activity and mobility (goal setting, adherence, feedback, education, problem solving, progression).

**DISCHARGE PLANNING**

Schedule HV (if required) for within 1-2 days of DC to set up technology

- Set up technology for home-setting
- Set up home-based folder (info to participants, community mobility safety, falls calendars, instructions for technology, contact details of research staff)

**INPATIENT INTERVENTION**

Provide daily 30-60mins inpatient technology-based intervention in pm as per protocol.

- utilise most useful technology for current mobility limitations, start and end with fun/ engaging/ successful technology.
- start to introduce and teach community-based technology to participant.
- promote independence and self-efficacy (and/or family involvement) ASAP as safety allows

**INITIAL TECHNOLOGY SESSION**

Initial session with participant: goal setting, discuss technology options, home environment, family support, current technology use and access, physical activity interests. Begin with fun/ engaging/successful technology

# MOBILITY LIMITATIONS TABLES

## Standing Up from a chair

| **Adaptive strategy/problem** | **Set-up** | **Easy games/exercises** | **Medium games/exercises** | **Hard games/exercises** |
| --- | --- | --- | --- | --- |
| **Weight borne principally through intact side** | Intact leg forward, on block, height of chair | **Humac** Weight bearing (“>” mode); Force vs. Time  **Fysiogaming** sit to stand (level 1-10); Assessment Centre  **Stepping tiles:** loading the leg in sitting; reaching in sitting; sit to stand  **iPad App: AMOUNT** preparation for standing up (low difficulty)  **T-Rex** exercises in sitting | **Humac** Weight bearing (“>” mode), Force vs. Time  **Fysiogaming** sit to stand (level 11-20)  **iPad App: AMOUNT** standing up (medium difficulty)  **Stepping tiles:** sit to stand | **Fysiogaming** sit to stand (level 21-30 or dynamic mode)  **iPad App: AMOUNT** standing up (high difficulty)  **Stepping tiles:** sit to stand |
| **Wide base of support** | Restrict BOS markers/blocks on ground, height of chair | **Fysiogaming** sit to stand (level 1-10); Assessment Centre  **Stepping tiles:**  sit to stand;  **iPad App: AMOUNT** standing up (low difficulty)  **T-Rex** exercises in sitting | **Fysiogaming** sit to stand (11-20)  **iPad App: AMOUNT** standing up (medium difficulty)  **Stepping tiles:** sit to stand | **Fysiogaming** sit to stand (21-30 or dynamic mode)  **iPad App: AMOUNT** standing up (high difficulty)  **Stepping tiles:** sit to stand |
| **Falls backwards** | Height of chair | **Humac** Force vs. Time; scale; luge  **Fysiogaming** sit to stand (level 1-10); Assessment Centre  **Stepping tiles:**  reaching in sitting; sit to stand;  **WiiUFit:** Core luge  **iPad App: AMOUNT** standing up (low difficulty); **T-Rex** exercises in sitting | **Humac** as for easy but progress difficulty level, boundaries of movement  **Fysiogaming** sit to stand (11-20)  **iPad App: AMOUNT** standing up (medium difficulty)  **Stepping tiles:** sit to stand | **Fysiogaming** sit to stand (21-30 or dynamic mode)  **iPad App: AMOUNT** standing up (high difficulty)  **Stepping tiles:** sit to stand |
| **Reduced speed of movement** | Height of chair | **Humac** Force vs. Time; scale; luge  **Fysiogaming** sit to stand (level 1-10); Assessment Centre  **Stepping tiles:** reaching in sitting; sit to stand  **WiiUFit:** Core luge  **iPad App**: **AMOUNT** standing up program, **T-Rex** exercises in sitting | **Humac** as for easy but progress difficulty level, boundaries of movement  **Fysiogaming** sit to stand (11-20)  **iPad App: AMOUNT** standing up;  **Stepping tiles:** sit to stand | **Fysiogaming** sit to stand (21-30)  **iPad App: AMOUNT** standing up; high difficulty  **Stepping tiles:** sit to stand |

## Maintaining a standing position

| **Adaptive strategy/problem** | **Set-up** | **Easy games/exercises** | **Medium games/exercises** | **Hard games/exercises** |
| --- | --- | --- | --- | --- |
| **Decrease ability to stand EWB** | BOS, environment set up (wall, table, chair, supervision) | **Humac:** CTSIB,Weightbearing; Weightbearing XY; Centre of Pressure  **Stepping Tiles:** standing EWB  **iPad APP:** **AMOUNT** Maintaining a standing position program; **T-Rex** standing exercises | **Humac:** as for easy but progress difficulty level, boundaries of movement | **Humac:** as for easy & medium but progress difficulty level, boundaries of movement |
| **Holding self stiff (avoids threats to balance)** | BOS, environment set up (wall, table, chair, supervision) | **Humac:** Stability; Mobility; Stability envelope; Limits of Stability Wightbearing XY,Targets, Random Motion;  **iPad APP:** **AMOUNT** Maintaining a standing position program; **T-Rex** standing exercises | **WiiFit:** Table tilt; heading; balance bubble; hula hoop; ski slalom; snowboard slalom; Perfect 10; Skateboard arena; snowball fight (modified)  **Xbox:** wall breaker; Fruit Ninja  **Humac:** as for easy but progress difficulty level & boundaries of movement; Roadway; Balance; Ski; Snowboard; Luge; Flight | **WiiFit:** tightrope; driving range; snowball fight  **Humac:** as for easy & medium but progress difficulty level & boundaries of movement; Pong; Breakout |
| **Decrease loading the affected leg** | BOS, environment set up (wall, table, chair, supervision) | **Humac:** Weightshift; Targets; Weightbearing XY  **WiiFit:** Penguin slide (modified), Big Top Juggling (modified)  **Stepping Tiles:** reaching in sitting, hip extension over the side of the bed, Shifting weight in standing  **iPad APP:** **AMOUNT** Maintaining a standing position program; **T-Rex** standing exercises | **WiiFit:** Table tilt, heading, balance bubble, ski slalom, ski jump, snowboard slalom, Perfect 10, Skateboard arena, hula hoop, tightrope (modified), Penguin slide, Tilt city (modified)  **Humac:** as for easy but progress difficulty level & boundaries of movement; Roadway ML; Balance; Ski; Snowboard; Luge; Flight | **WiiFit:** Ski jump; tightrope; driving range; snowball fight; Tilt city; Big Top juggling  **Humac:** as for easy & medium but progress difficulty level & boundaries of movement; Pong ML; Breakout ML; Roadway |

## Reaching while Standing

| **Adaptive strategy/problem** | **Set-up** | **Easy games/exercises** | **Medium games/exercises** | **Hard games/exercises** |
| --- | --- | --- | --- | --- |
| **Flexes at hips and/or protracts shoulder instead DF at ankles when reaching forward** | Set targets to reach for Fysiogaming, environment set up (wall, table, chair, supervision) | **Humac:** Weight bearing (˄); **Fysiogaming:** leaning trunk forward and backward; all sides (level 1-10); Reaching (level 1-10)  **iPad APP:** **AMOUNT** Reaching in standing program  **Stepping Tiles:** shifting weight in standing | **Humac:** as for easy but progress difficulty level & boundaries of movement  **Xbox:** 20,000 leaks; wall breaker  **Fysiogaming:** leaning trunk forward and backward; all sides (level 11-20); Reaching (level 11-20) | **Humac:** as for easy & medium but progress difficulty level & boundaries of movement  **Fysiogaming:** leaning trunk forward and backward; all sides (level 21-30); Reaching (level 21-30) |
| **Moving trunk instead of moving at hips and ankles when reaching sideways** | Set targets to reach for Fysiogaming, environment set up (wall, table, chair, supervision) | **Humac:** Weight shift  **Fysiogaming:** leaning trunk to the sides, all sides (level 1-10)  **iPad APP:** **AMOUNT** Reaching in standing program  **Stepping Tiles:** shifting weight in standing | **Humac:** as for easy but progress difficulty level & boundaries of movement  **WiiFit:** snowboard slalom, Wii hula hoop  **Fysiogaming:** leaning trunk to the sides, all sides (level 11-20) | **Humac:** as for easy & medium but progress difficulty level & boundaries of movement  **Fysiogaming:** leaning trunk to the sides, all sides (level 21-30) |

## Stepping while standing

| **Adaptive strategy/problem** | **Set-up** | **Easy games/exercises** | **Medium games/exercises** | **Hard games/exercises** |
| --- | --- | --- | --- | --- |
| **Decrease speed of stepping** | environment set up (wall, table, chair, supervision) | **Fysiogaming:** walking sideways (level 1-10); lunges; dynamic balance (level 1-10); walk in place (level 1-10)  **iPad APP:** **AMOUNT** Stepping in standing program; **T-Rex** standing exercises | **WiiFit:** jogging plus; step basic (modified)  **WiiFamily Trainer:** stone stepper, sprint challenge  **Xbox:** 20,000 leaks; space pop; stomp it (modified); run the world  **Fysiogaming:** walking sideways (level 11-20); dynamic balance (level 11-20); walk in place (level 11-20) | **WiiFit:** Cycling; step basic;  **WiiUFit:** hosedown, dessert, obstacle course, mole stomper  **Xbox:** river rush; rally ball; reflex ridge; stomp it; **Fysiogaming:** walking sideways (level 21-30); dynamic balance (level 21-30); walk in place (level 21-30) |
| **Increase time spent in double support** | environment set up (wall, table, chair, supervision), | **Fysiogaming:** side stepping; side strides; lunges; hip abduction  **Stepping Tiles:** stepping grid exercise; stepping exercise; stepping up to touch a block;  **iPad APP:** AMOUNT Stepping in standing program, **T-Rex** standing exercises | **WiiFit:** tightrope (modified)  **WiiFamily Trainer:** stone stepper, sprint challenge, mine cart adventure (modified)  **Xbox:** juggle it; kick it | **Fysiogaming:** hip abduction  **WiiFit:** tightrope  **WiiFamilyTrainer:** BMX speed, mine cart adventure  **WiiUFit:** hosedown, dessert, obstacle course, mole stomper  **Xbox:** stack em up |
| **Short step length** | environment set up (wall, table, chair, supervision) | **Fysiogaming:** dynamic balance (level 1-10); side stepping; side strides; lunges  **Stepping Tiles:** stepping grid exercise, stepping exercise; **iPad APP:** **AMOUNT** Stepping in standing program; **T-Rex** standing exercises | **Xbox:** 20,000 leaks; stomp it (modified)  **Fysiogaming:** dynamic balance (level 11-20)  **Stepping Tiles:** as for easy but add bridging tiles  **iPad APP:** **AMOUNT** Stepping in standing program; **T-Rex** standing exercises | **Xbox:** stomp it **Fysiogaming:** dynamic balance (level 21-30); hip abduction  **iPad APP:** **AMOUNT** Stepping in standing program; **T-Rex** standing exercises |
| **Trunk inclined forward when stepping with the unaffected leg** | environment set up (wall, table, chair, supervision) | **Stepping Tiles:** hip extension over the side of the bed; stepping grid exercise; stepping exercise; stepping up to touch a block;  **iPad APP:** **AMOUNT** Stepping in standing program; **T-Rex** standing exercises | **Stepping Tiles:** as for easy but add bridging tiles, increase height block  **iPad APP:** **AMOUNT** Stepping in standing program ; **T-Rex** standing exercises | **iPad APP:** **AMOUNT** Stepping in standing program; **T-Rex** standing exercises |
| **Increased hip circumduction in swing** | environment set up (wall/block to restrict unwanted mvt, block height,) | **Fysiogaming:** knee and hip flexion, knee flexion (level 1-10, 50% ROM)  **iPad APP: AMOUNT** Stepping in standing program; **T-Rex** standing exercises  **Stepping Tiles:** stepping exercise | **Fysiogaming:** knee and hip flexion, knee flexion (level 1-10, 100% ROM)  **iPad APP: AMOUNT** Stepping in standing program; **T-Rex** standing exercises  **Stepping Tiles:** as for easy but add bridging tiles  **WiiFamilyTrainer:** stone stepper modified | **Fysiogaming:** knee and hip flexion, knee flexion (level 11-30, 100% ROM)  **iPad APP AMOUNT** Stepping in standing program; **T-Rex** standing exercises |

## Changing directions while walking

| **Adaptive strategy/problem** | **Set-up** | **Easy games/exercises** | **Medium games/exercises** | **Hard games/exercises** |
| --- | --- | --- | --- | --- |
| **Decrease speed changing direction** | environment set up (wall, table, chair, supervision) | **Fysiogaming:** walking sideways (level 1-10), walk in place (level 1-10)  **iPad APP: AMOUNT** changing directions while walking program | **WiiFit:** step basic (modified)  **WiiFamilyTrainer:** mole stomper  **Xbox:** 20,000 leaks; **Fysiogaming:** walking sideways (level 11-20), walk in place (level 11-20)  **iPad APP: AMOUNT** changing directions while walking program | **WiiFit:** step basic ;  **WiiUFit:**  dessert, ultimate obstacle course, scuba search  **Fysiogaming:** walking sideways (level 21-30), walk in place (level 21-30)  **iPad APP: AMOUNT** changing directions while walking program |
| **Shuffling feet to change direction** | environment set up (wall, table, chair, supervision) | **Stepping Tiles:** stepping grid exercise, stepping exercise  **iPad APP: AMOUNT** changing directions while walking program | **Stepping Tiles:** as for easy but add bridging tiles  **iPad APP: AMOUNT**changing directions while walking program | **iPad APP: AMOUNT** changing directions while walking program |
|  |  |  |  |  |

## Stairclimbing

| **Adaptive strategy/problem** | **Set-up** | **Easy games/exercises** | **Medium games/exercises** | **Hard games/exercises** |
| --- | --- | --- | --- | --- |
| **Decreased hip and knee extension when ascending stairs** |  | **Stepping Tiles:** Stepping up and down on a block  **iPad APP:** **AMOUNT** Climbing stairs program | **WiiFit:** step basic (modified)  **Fitbit**  **Stepping Tiles:** as for easy but increase height of block  **iPad APP:** AMOUNT Climbing stairs program | **Fitbit**  **Stepping Tiles:** Stepping up and down on a block  **iPad APP:** **AMOUNT** Climbing stairs program |
| **Decreased eccentric control knee extensors on descent** |  | **Stepping Tiles:** Stepping up and down on a block  **iPad APP: AMOUNT** medium difficulty | **Stepping Tiles:** as for easy but increase height of block  **iPad APP: AMOUNT** medium difficulty | **iPad APP: AMOUNT** high difficulty |
|  |  |  |  |  |

## Physical activity throughout day

| **Adaptive strategy/problem** | **Set-up** | **Easy games/exercises** | **Medium games/exercises** | **Hard games/exercises** |
| --- | --- | --- | --- | --- |
| **Prolonged periods of sitting** |  | Any technology above that the person is standing to participate in. | **Fitbit** or **Garmin watch**  **iPad APP:** **AMOUNT** Physical activity throughout the day program | **Phone APP:** Runkeeper; SMS global  **iPad APP:** **AMOUNT** Physical activity throughout the day program |
| **Prolonged overall sedentary time** |  | Any technology above that the person is standing to participate in. | **WiiFit:** jogging, step basic (modified), free stepping (slow pace); **Fitbit** or **Garmin watch**  **iPad APP:** **AMOUNT** Physical activity throughout the day program | **WiiFit:** cycling, step basic, free stepping (fast pace)  **Phone APP:** Runkeeper, SMS global |
| **Decrease steps throughout the day** |  | Any technology above that the person is stepping in standing. | **WiiFit:** jogging, step basic (modified), free stepping (slow pace); **Fitbit** or **Garmin watch**  **iPad APP:** **AMOUNT** Physical activity throughout the day program | **WiiFit:** cycling, step basic, free stepping (fast pace)  **Phone APP:** Runkeeper, SMS global  **iPad APP:** **AMOUNT** Physical activity throughout the day program |

# DEVICES

## NINTENDO WII

### Getting started with Wii

- 1. Turn on power and load the software WiiFit Plus. You will need a Wiimote, balance board and nunchuk to play all the games in WiiFit Plus (Note: check batteries and ensure you have spares ready to go)
  2. You will start at the main menu of Wii. There are a number of different boxes to choose, there are 3 that are relevant: 1) top left hand corner is the software that you have loaded, select that to play the games. 2) Mii Channel is 2^nd^ top left box with lots of faces, this lets you create a Mii (see below). 3) Envelope down bottom right hand corner, this links you to the calendar which records play time, you can use this to verify play time if participant independent, or to record it even if you are supervising. Select envelope and select calendar, then choose which date you wish to view, it will display different play times (if there has been a break in game play, and total time for that day.

### Creating a new Mii

You could create a Mii for each participant if they want, but otherwise you could perhaps make a male and female and use for all inpatients. Creating their own takes time, but that Mii could top the leader board of games and motivate in that way.

- 1. Go to Mii channel
  2. Select “Start”
  3. Choose smiley file icon with + sign.
  4. Select male or female
  5. You can start from scratch but quicker to select “choose a look-alike”
  6. You can modify different features e.g. hair, eye colour etc.
  7. Once you have finished making modifications, select “quit” and then “save and quit”.
  8. Enter a name
  9. Return to the Wii menu (arrow circle top left hand side)

### Getting started with WiiFit

- 1. Select WiiFit (at top left hand corner of Wii menu) and Start
     1. If you haven’t put your Mii into WiiFit you need to do this first (you might want to do this before the participant comes) unless you want to show them how to do it. (Note: if you don’t want to create a Mii and just want to get started, you can select the trial button (outline of person with ? in middle and select from 6 Mii’s they give you).
     2. The Smiley face with the + sign on the left side lets you add a Mii you have previously created in the Mii Channel.
     3. Select “create”
     4. Press the A button to quickly scroll through the balance board talk and select the Mii for the participant you are working with and hit yes.
     5. Add height and DOB for participant
     6. Press the A button to quickly scroll through the balance board talk and turn on balance board as per picture on screen
     7. Select “training” to go to the games in WiiFit Plus. The categories are : training plus, yoga, muscle workouts, aerobic exercises, balance games

### Game selection

- - Don’t start with games that don’t allow error (WiiFit Balance: balance bubble and tightrope, zazen), select game where certain level of success can be achieved.
  - WiiFit plus training plus games: Rhythm Kung Fu , Segway Circuit, Bird’s eye-bull’s eye, rhythm parade, table tilt plus, balance bubble plus deemed not appropriate for rehabilitation populations in this trial.
  - WiiFitplus balance exercises: balance tests (basic balance test, agility test, stillness test, dual balance test, prediction test, peripheral vision test, judgement test) not appropriate for this trial as only last short time, you cannot select which test to do and there are a lot of screens to be navigated through to complete the short test.

### Getting started with Wii Family Trainer

Family Trainer/Family Trainer Extreme Challenge

1. You will need the Game Mat, and Wiimote to play the games.
2. After inserting the CD into the Wii Console, with your Wii remote select “family trainer”/”family trainer extreme challenge” (the first box in the top left corner)
   1. To select, point towards the item you wish to select on the screen and press “A”
3. Select “start”
4. Select “A to start”
5. Mode select screen: select “single player”
6. Select character
   1. OR “new”
      1. Select face
      2. Type name
      3. Select body type
7. Select “free play”
8. Choose the game to play (there are down/up arrows on the right side of the screen to scroll)

### Other helpful hints for using the Wii

- 1. Home button takes you to option to Wii Menu.
  2. When in WiiFit game, it is often easier to use arrows than move the Wiimote

| WII FIT | | | | | | | | | | | | | | | | | | | |
| --- | --- | --- | --- | --- | --- | --- | --- | --- | --- | --- | --- | --- | --- | --- | --- | --- | --- | --- | --- |
| **Mobility activity** | **Game** | **Game length** | | | **Description** | | | **Movement/ Feedback** | | | **Progress/ Motivation** | | | **Issues/ Additional demands** | | | **Rehabilitation**  **modifications** | | |
| **Sitting** | WiiFit balance/ Zazen | Game stops when move | | | The player is required to sit on the balance board and maintain a quiet seated position so that the candle on the screen does not blow out. | | | No movement/ Candle flame starts to move if you start moving, does not allow error. | | | you can maintain position/ time sitting still, time game, leaderboard | | | Only records when movement occurs, can start unbalanced and move to more balanced position and it will interpret that as negative | | | Place balance board on plinth with thigh support and feet flat on ground. Start person in balanced position. | | |
| **Standing** | WiiFit balance/  Heading | ~60secs | | | The player is required to move weight between their legs on the balance board to ‘head’ soccer balls being kicked towards them | | | ML direction / total score at end, allows errors | | | ↑ target frequency/ leaderboard | | | Mocking, fast paced/ must miss other objects thrown e.g. shoe | | | Set small target number of balls to head in timeframe | | |
|  | WiiFit  Balance/ Ski slalom | ~60secs | | | The player is required to ski down a mountain between flags by moving weight between their legs on the balance board | | | All directions / total score at end, allows errors, | | | None/ leaderboard | | | Mocking/ nil | | | Use dot representing COM rather than person skiing to guide performance | | |
|  | WiiFit  Balance/ Ski jump | <60secs 2 jumps | | | The player is required to stand on balance board and start in a squat position, straighten knees to do a ski jump | | | COM forward, knee extension / distance jumped, allows error | | | None/ leaderboard | | | Mocking/ requires getting timing correct to extend knees | | | Use dot representing COM rather than person skiing to guide performance | | |
|  | WiiFit  Balance/  Table tilt | ≤5mins ↑ time with stages | | | The player is required to move their weight on the balance board to guide a ball(s) into a hole | | | All directions/ ball goes into hole, allows error | | | ↑ target number / leaderboard, stages achieved | | | Mocking/ requires timing to get ball in hole | | | Nil | | |
| **Standing** | WiiFit  Balance/*  Tightrope tension | ≤ 2mins game stops if fall off | | | The player is required to step on the spot and move weight (SLS) between their legs on the balance board to walk along the tightrope, semi-squat then extend to avoid objects | | | ML direction/ does not allow error, fb distance walked before fall, time taken to complete, | | | In game / leaderboard | | | Performance was better with SLS | | | Can perform as step touch exercise to block infront | | |
|  | WiiFit  Balance/*  Balance bubble | ≤1:30 min game stops if bubble bursts | | | The player is required to move their weight on the balance board to guide their character along a river without colliding with any objects | | | Keep COM forward and move ML direction/ does not allow error, fb distance achieved or time to complete | | | ↓ river width, ↑ choice/ leaderboard | | | Game can end straight away if hit riverbank/ | | | Nil | | |
|  | WiiFit  Balance/ Penguin slide | ≤ 90 secs | | | The player is required to move their weight on the balance board to tilt an iceberg upon which a penguin is feeding, to guide the penguin to the fish | | | ML direction/ score at end, allows error | | | ↑ target number/ leaderboard, previous score | | | Mocking/ Requires timing to get fish | | | Lots of options to modify: slow weight shift each side & fall off each side; balance in middle & therapist direct which way to go, fast shift SLS, step touch to block and count how many times fall off, | | |
|  | WiiFit balance/  Snowboard  slalom | | | ~ 60 secs | | | The player is required to snowboard down a mountain between flags by moving their weight on the balance board (board side on) | | | COM (L) leg, AP direction/ score at end, allows error | | | None/ leaderboard, previous score | | | Fast paced, difficult to use snowboard feedback | | | Aim achieve certain number of flag checkpoints |
|  | WiiFit training plus/ Perfect 10 | | | ~ 60secs | | | The player is required to move towards the numbers to add up to 10 by moving their weight on the balance board | | | ML and AP directions/ score at end, allows error | | | ↑ number choices/ leaderboard previous score | | | Dual task solve maths problem with movement | | | Nil, can work at own pace. |
| **Standing** | WiiFit training plus/ driving range | | | ≥ 5mins 20 balls to hit | | | The player is required to swing a golf club by shifting weight between legs on balance board and swinging UL with remote (board side on) | | | ML direction/ ranks your shots, total score | | | Difficulty level/ leaderboard | | | Dual task (Physical + cognitive) swing arm, hold button down and shift weight | | | Nil, would only use if very keen golfer and interested. |
|  | WiiFit training plus/ snow ball fight | | | ≤90 secs | | | The player is required to throw snowballs at opponents by moving weight on the balance board and aiming with remote | | | ML direction/ score at end, allows error | | | In game, ↑ target frequency/ leaderboard, previous score | | | Fast paced, requires timing and use of UL with movement | | | Start with aim to not get hit with snowballs, rather than throw snowballs as well |
|  | WiiFit training plus/ Tilt City | | | ~ 2mins | | | The player is required to move their weight on the balance board and rotate the Wiimote to guide falling balls into matching colour pipes | | | ML direction/ score at end, allows error | | | In game, ↑ target number, frequency | | | Fast paced, requires timing, use of UL and cognitive decisions for correct colour ball | | | Choose 1 colour ball to get in correct colour pipe. |
|  | WiiFit training plus/ Big Top juggling | | | 2mins | | | The player is required to shift their weight in a small range side to side to stay close to the middle on the balance board while juggling balls using the WiiMote and nunchuk | | | ML direction/ score at end, allows errors | | | In game, ↑ target number, frequency | | | Dual task (physical + cognitive) Requires correct timing and use of UL for juggling | | | Lots of options to modify: can start without juggling and focus on weight shift-slow weight shift each side fast weight shift keep in middle, balance in middle & therapist direct which way to go, SLS, step touch to block, |
|  | WiiFit training plus/ skateboard arena | | |  | | | The player is required to skate through a specific track by moving weight between legs on balance board (board forward) | | |  | | |  | | | ? May be suitable for younger population | | |  |
| **Stepping in standing** | WiiFit training plus/ obstacle course | | ?? | | |  | | |  | | |  | | | Fun but difficult | | |  | |
|  | WiiFit training plus/ jogging plus | | 10 mins | | | The player is required to follow a Mii to complete a jogging track (balance board not required, remote in pocket or hand) | | | Stepping on the spot/ | | | ?? | | | ??Asks 3 questions at end of the surroundings | | | Can walk on spot not jog, allows you to stop if needed. | |
|  | WiiFit aerobic exercises/ Hula hoop | | ?3mins | | | The player is required to rotate their hips to keep the hoops spinning, need to lean to one side with arms up to catch new hoops | | | All directions, ML direction to get hoops/ total score, allows error. | | | Change to super hula game, ↑ duration and change direction | | | Timing of leaning to side and lifting arms up to get hoops, not always sensitive to movement. | | | Just focus on hip rotation, don’t try to get new hoops | |
| **Stepping in standing, changing directions** | WiiFit aerobic exercises/ Step basic | | 3mins | | | The player is required to step up and down forward and to the sides on the balance board on the coloured footprints on screen which indicate timing and direction | | | Stepping forward, backward, right and left sides/ ?? , allows error | | | Change to step plus game, ↑ duration, speed and change of direction | | | Fast pace, timing + cognitive to step with correct foot, risk of falls, recommend close supervision | | | Could start with forward steps only, give standing rest when goes sideways | |
|  | WiiFit aerobic exercises/ Free stepping | | Can set time or number of steps | | | The player is required to step up and down forward and to the sides on the balance board in time with metronome (can watch other programs on TV while doing it) | | | Stepping forward, backward | | | In game increase speed of metronome, number of steps, duration | | | Timing to step with foot, risk of falls, recommend close supervision | | | Nil | |

| WII FAMILY TRAINER | | | | | | | |
| --- | --- | --- | --- | --- | --- | --- | --- |
| **Mobility activity** | **Game** | **Game length** | **Description** | **Movement/ Feedback** | **Progress/ Motivation** | **Issues/ Additional demands** | **Rehabilitation**  **modifications** |
| **Stepping while standing** | Wii Family Trainer/Stone Stepper | Game finishes when course completed (running timer) | The player is required to stand on the game mat and step on either one of the two middle tiles in order to move jump from pillar to pillar on a straight course while avoiding wobbly red pillars. | Stepping with left or right foot in standing/ character moves forward on screen, time, written feedback (i.e. “miss” if fallen off pillar), allows for error | Nil/ leaderboard, completing course in a faster time | Negative feedback at end if record not beat (sad face, “try again”) | Instead of standing on the middle two tiles and stepping on the spot, the player can stand on the two arrows pointing down instead and step *forward* onto the middle tiles as required |
|  | Wii Family Trainer/Sprint Challenge | Game finishes when the track is finished (“goal” line at end) | The player is required to stand on the game mat and step on the two middle tiles with alternate feet in order to run through a straight course. | Stepping on the spot with alternating feet/character moves forward, speed (mph), time, allows for error | Nil/ leaderboard, completing course in a faster time, increasing speed (mph) | Negative feedback at end if record not beat (sad face, “try again”), character on screen moves quite slow if not stepping fast | Instead of just stepping, the player can march with high knees, using their own hands with elbows held at 90 degrees in front of them as targets for their knees to reach; or, a block can be placed in front of the player to do alternating step taps; or, the player can stand in step stance with one foot on the front arrow and one foot on the back arrow and practice weight shifting while lifting the foot opposite to the weight shift |
|  | Wii Family Trainer/Mole Stomper | ~30 seconds | The player is required to stand on the middle two tiles of the game mat and steps on any one of the 6 arrows in order to stomp on the mole(s) as it pops out of the holes. | Stepping in all directions/hammer hits mole, time, score of moles hit, allows for error | Increase number of moles stomped on/leaderboard | Player may cheat by just using one leg to stomp on any of the arrows/If two moles pop up at the same time this may require jumping – however, not necessary to hit both | Ensure that player is only stepping on the orange arrows with his/her R) foot and the blue arrows with his/her L) foot. |
|  | Wii Family Trainer Extreme Challenge/BMX Speed | ~20 seconds | The player is required to stand on the two middle tiles of the game mat and cycle through a track by alternate stepping on the mat and shaking the remote in order to accelerate forwards. | Alternate stepping on the spot/cycle moves forward, speed (mph), time | Nil/leaderboard, speed | Negative feedback at end if record not beaten/Dual UL task, need fast stepping to move more than 5mph, short time of 20seconds | Nil |
| **Stair Climbing** | Wii Family Trainer/Mine Cart Adventure | Game finishes when course is completed | The player is required to stand on the middle tiles of the game mat and steer a cart through the track by lifting one leg up in order to go around bends without falling. Meanwhile, holding the Wii remote horizontally and moving it up and down with two hands to accelerate. | Single leg stance and hole with UL elbow flexion/extension/cart moves forward, time, speed (mph), instructions RE: which leg to lift, allows error (if leg not lifted, cart tips off and game returns to the point in which the cart fell off) | Nil/time, leaderboard | Additional cognitive demands in knowing which leg to lift (with assistance of instructions), dual task of UL to accelerate | Player can play without the UL flexion/extension for simplification – the cart will move slower; a target may be placed (e.g. Cup on a table) on the left and right of the player as feedback for leaning from side to side with each bend; a block may be placed in front of the player to do step taps instead of single leg stance (player may stand on the front two arrows instead of the two middle tiles to allow more room for the block placement) |

## NINTENDO WII U

### Setting up the Wii U console and GamePad

- Turn on the power via the console and turn the TV to the appropriate HDMI or AV input
- Turn the Wii GamePad on and then sync the gamepad by pressing the red button on the front of the Wii U console and the red button on the back of the gamepad(you will need a pen or something thin to press the sync button on the gamepad)
- Follow the instructions on the gamepad for set-up
- Wii U will automatically configure TV display to TV (thus need to set up on the tv the client will be using)
- Skip set up for TV remote (‘Not Now’)
- Set up Sensor bar
  - Above or below tv depending on where it is most stable
- Continue through instructions on Gamepad
- Connect to internet
  - If having difficulty connecting to internet, need to manually change settings (see below)
  - <http://en-americas-support.nintendo.com/app/answers/detail/a_id/1643/~/how-to-manually-set-up-an-internet-connection>
- Follow instructions on GamePad
- GamePad will automatically take you to setting up a new Mii
- Link the Nintendo Network ID or skip
- “Do you wish to automatically receive software from Nintendo via SpotPass”- select Don’t Receive

### Introduction to Homepage on GamePad and TV screen

- TV screen and GamePad will show two separate displays of the Homepage, to switch between which one is displayed on the TV or on the Gamepad, press the button in the top right corner or press the X button
  - The display you want is the one with the boxes appearing on the gamepad so you can select the menus. The screen with the many Mii characters is not relevant and thus should not be displayed on the gamepad
- There are a number of different boxes to choose from
  - Top left is the software that you have loaded, select that to play the games
  - Mii Maker is 2^nd^ top left box with lots of faces, this lets you create a Mii (see below)
  - The bottom left box with the green bar graph is the ‘Daily Log’. This links you to the calendar which records play time, you can use this to verify play time if participant independent, or to record it even if you are supervising.

### Getting started with WiiFit Plus U

- 1. Turn on Wii U console by pressing power on button either on GamePad or Console
  2. Insert WiiFit Plus U disc into Wii Console
  3. If Mii character has not been made, first create a Mii under the Mii Maker menu (see below) and select this player as the user
  4. Select WiiFit Plus U logo (at top left hand corner of Wii menu – image of women doing yoga stance)
  5. Wait for system memory to be updated ~2-5 minutes
  6. Any previous Wii Fit Plus data will be transferred to Wii Fit U (<1 min)
     1. Can later choose whether to transfer data or start as a new user
  7. The Balance Board cartoon will provide instructions on what to do (can either watch on gamepad or on TV). Player must hold gamepad to navigate through menus
  8. Follow through with Body Balance Tests
  9. Once WiiFit Age has been given, will take player to main screen. Press ‘Start’
  10. Press ‘Training’ (see below for navigation through Wii Plaza
  11. Press ‘Select Exercise’
  12. Choose exercise from menu
  13. Sync the WiiMote for easy navigation through menus whilst on balance board – so do not have to hold GamePad (See below)

### Creating a Mii Character

You can create a Mii from the homepage. You could create a Mii for each participant if they want, but otherwise you could perhaps make a male and female and use for all inpatients. Creating their own takes time, but that Mii could top the leader board of games and motivate in that way. You will need both the GamePad and the TV for this.

1. Tap on the Mii Maker
2. Select ‘Create Mii’
3. Choose either ‘Create Mii from Features’ or ‘Create Mii From Photo’
4. If Creating from Features
   - 1. Choose gender
     2. You can modify different features e.g. hair, eye, colour etc.
     3. Enter in nickname, birthdate and favourite colour
     4. Press ‘Save’ when finished
5. If creating from Photo
   - 1. Choose gender, skin tone, eye colour, hair colour, hairstyle
     2. Take photo with gamepad (camera is on the front of gamepad)
     3. Select a face that best suits the client/they one they prefer
     4. Change any features such as hair style, eyebrows, eye style, nose etc.
     5. Enter in nickname, birthdate and favourite colour.
     6. Press ‘Save’ when finished
6. Return to homepage by pressing Exit (in bottom left corner of gamepad)
7. Select new player as new user by pressing icon with current user’s Mii face. Press switch
8. Press ‘add new user’
9. Press ‘ choose a Mii From Mii Maker’
10. Select user’s Mii character and press ‘Register’
11. Can/cannot link a Nintendo ID
12. Do not apply parental control
13. Will take you back to main page

### Navigating through main menu

- Body test:
  - In bottom left corner of screen
  - Will take you to body tests (balance)
- Training:
  - In bottom right corner of screen
  - Takes you to games
- In the background there are a number of menus that you can navigate through by swiping left or right over them
  - Album
    - Shows pictures of exercises completed on wii u
  - Notice Board
    - Where your achievements in Wii Fit U are displayed
  - Calendar
    - Can check photos taken by Body Test (only really for weight loss – not relevant for this trial)
    - Can select a day and see the photo, BMI, and CoG results from Body test
  - Graphs
    - Calories burned and distance walked
    - Will see data from Wii Fit U and Wii Fit Meter (steps taken, calories burned)
    - Can change the graph on the left and right side to look at
      - Weight, BMI, Wii Fit Age, Waist size, **Calories burned**, FitCash, **Steps, Distance walked**
    - Can manually enter in data under ‘record’ icon
  - Fit Meter Guide
    - Can use the Fit Meter in the Wii U to measure the calories you burn in daily activities
    - Has an accelerometer and a pressure sensor built in
    - These track METs (intensity of activity) as you move around
    - Records steps, calories burned (includes calories burned going up and down stairs/hills), altitude changes
    - Can send Fit Meter records to Wii Fit U 🡪 greater detail on gamepad
      - Can use this data in Wii Fit U to tackle courses from around the world
    - If you don’t meet goals for calories during the day, can plan a workout in the Wii Fit U for the remaining calories
    - Can register Fit Meter
      - When syncing the Fit Meter, hold down middle button and aim it at the infrared sensor on the GamePad (black square next to earphone jack)
    - Once you register the Fit Meter the Fit Meter Guide menu will be replaced with “Fit Meter Data” and “Fit Meter Challenge” and a Fit Meter icon will appear next to the Mii character
  - Fit Meter icon next to Mii Character
    - Press this when you want to sync data from Fit Meter to gamepad
    - Hold down on middle button on Fit Meter and point at infrared sensor
    - Screen will show how many calories burnt today
      - Can press calorie checker to see how much that equates to for common foods
  - Fit Meter Data
    - Displays any information from Fit Meter
    - Eventually data will be over-rided – can save certain sessions by pressing ‘lock’. Then can add a comment to it
    - Can change graph from 2 hr to 24 hr period
    - Can swipe right or left across graph to see activity over different hours
    - Different colours correspond to different types of activities
  - Fit Meter Challenge
    - Use fit meter data and challenge yourself to complete courses from all round the globe
    - Chose walking or climbing challenge

### Navigating through training menu

- Touch and slide panels on the Wii U GamePad to scroll from left to right
- Tap a panel to select it
- Rankings
  - Can view various rankings to do with training
  - Can see this against all users or just within one user
  - Shows you which games are most played, recently played, calories burned, and time played
- Gym Community
  - Can share training details with other users
  - Need a Nintendo ID for this
- Select Exercise
  - This is where all the exercise games are
  - Choose between Yoga, Muscle, Aerobic, Dance and Balance (total of 74 games)
- Personal Trainer
  - Can create a workout for you based on the calorie goal or exercise time set
  - Can set training type and intensity
  - Can choose based on calorie (gives examples of foods)
    - Can choose type – out of all the exercise menus) or random
    - Can choose intensity (low, medium, high or random)
    - Press ‘begin’
  - Based on exercise time
    - Up to 60 minutes
    - Can choose type
    - Can choose intensity
    - Press ‘begin’
- Wii Fit U Routines
  - Can choose between lifestyle, health, youth, form and vitality
  - Choose which category you want to address and then choose a subcategory
  - Will choose 3 example exercises and tell you how long it will take and how many calories you will burn
- My Routines
  - Can create a routine by adding items
  - Can choose up to 30 exercises
  - Can select exercise and click on it to change from – explanation to 6 reps

### How to Pair a Wii Remote with the Console

- Press ‘Pair Wii Remote’
- Press the sync button on the controller you want to pair
  - Note: you will need a paper clip or a thin object to be able to push the sync button
  - TV and GamePad display will say whether the pairing was complete
  - Press (B) or Exit

### What to do if having difficulty setting up internet

- First ensure that router is on and that the SSID and password is correct
- If internet is working but the Wii is having trouble connecting to the router you will need to connect to the internet manually
  - You will need a computer to locate the network’s IP address information
    - Windows:
      - Select start button
      - Type CMD and press enter
      - Type ipconfig/all and press enter
      - IP information should be displayed in a list – need to scroll through list and find IP address (or IPv4 address), subnet mask, default gateway and DNS servers
    - Mac
      - Select ‘system preferences’ from apple icon in top left corner
      - Select ‘network’ under ‘internet and network’
      - On the left side, select the type of connection you are using (airport, Ethernet etc. ) the active connections should say ‘connected’
      - Select ‘advanced’
      - Click the TCP/IP button
      - The router’s IP address is listed as Router
      - The IPv4 address, subnet mask, default gateway and DNS servers should not be displayed
  - From the Wii U Menu, select ‘System settings’
  - Select the ‘Internet’ icon
  - Tap ‘connection types’ or press the Y button
  - Select ‘manual connection’
  - Enter the following information
    - SSID = the Wi-Fi’s network’s name (case sensitive)
    - Security: select the encryption method (if you are unsure of what it is, it should say it on the router or go to the computer, look at the wireless network connection, right click and go to properties and it will be under security type
  - Tap IP address and select ‘don’t auto-obtain’ and enter the following information
    - IP address: but add 10 to it. E.g. if the computer’s IP address displays 192.168.2.5 you will enter 192.168.2.15
    - Subnet Mask: as displayed when searching for IP address. Often it is 255.255.255.000
    - Gateway: as displayed when searching for IP address
  - Tap confirm
  - An on-screen message will ask you to configure the DNS settings. Tap ‘Configure’
  - Tap ‘Don’t auto-obtain’ and enter the follow information
    - Primary DNS: enter 8.8.8.8 then tap ‘OK’
    - Secondary DNS: enter 8.8.4.4 then tap ‘OK’
  - Tap ‘Confirm’
  - Tap ‘Save’ or press the B button. Tap “save’ again
  - Tap ‘Connection test’ to test the connection
  - If internet is still not connecting, check the password and SSID was entered correctly and ensure the internet is working on another device

### Wii Motion Plus

- Is an expansion device for the Wii Remote video game controlled that allows it to more accurately capture complex motion
- The sensor in the device supplements the accelerometer and sensor bar capabilities of the wii remote to enable actions to be rendered identically on the screen in real time
- The original Wii Remote cannot be used with Wii Play: Motion and other games designed solely for use with Wii MotionPlus technology as such games take advantage of the technology to offer an even greater level of control and precision during gameplay
- Need a WiiMotionPlus™ accessory or a Wii Remote™ Plus which has this new feature built in
- Can use an original Wiimote to replace a nunchuck
- Which games require the Wii Motion Plus Remote
  - Balance Games: Free climbing
  - Aerobic Exercises: Puzzle Squash; Rowing Regatta
  - Dance: All dance games

1. Use the Wii remote to select ‘ TV Only’
2. Select WiiFit (at top left hand corner of Wii Menu) and press ‘Start’
3. Sync the balance board by pressing the red button on the bottom of the balance board and the red button on the console
4. Read instructions on screen and press A to continue

### Other helpful hints for using the Wii U

1. When in WiiFit game, it is often easier to use arrows than move the Wiimote
2. Pressing the Power button on the gamepad will turn the console on – can enable or disable this
3. Buttons generally mean:
   - 1. A = enter/continue
     2. B = back/exit
     3. X = continue/swap screen
     4. + = pause game and go to menu 🡪 continue playing, retry game or quit and return to exercise menu
     5. Home button = gives option to go to Wii Menu
     6. Arrows = let you navigate through buttons instead of using wii remote as a cursor

| WII U FIT | | | | | | | |
| --- | --- | --- | --- | --- | --- | --- | --- |
| **Mobility activity** | **Game** | **Game length** | **Description** | **Movement/ Feedback** | **Progress/ Motivation** | **Issues/ Additional demands** | **Rehabilitation**  **modifications** |
| **Preparation for standing up** | WiiFit balance/ Core luge | Game finishes when course complete (running timer) | The player is required to sit on the balance board and shift weight in all directions to manoeuvre a sled through a course | All directions in sitting/ speed and direction of sled movement, time through course | Nil/ leaderboard, completing course in a faster time | Requires fast AP movement to initially move the sled, requires sitting balance | Place balance board on plinth with thigh support and feet flat on the ground. Reach forward towards a target at the beginning to initiate sled movement. Use dot representing COM rather than person to guide performance |
| **Stepping in Standing**  **Stepping in Standing** | WiiFit balance/ Hose down | 120secs | The player is required to stand with one leg on the balance board and shoot water at oncoming targets by leaning forward to increase weight through the front leg. In order to ‘refill’ hose with water, weight must be taken off the front leg (e.g. Leaning back or stepping off) Meanwhile, using the game pad to aim the hose at the targets. | AP direction in step stance/ strength of water stream increases with increased weight through leg on balance board, allows errors, score of number of hit targets | None/ leaderboard, increasing score of targets hit | Fast paced, must coordinate UL/LL simultaneously | Set small number of targets to hit during the allocated time, while encouraging full strength of hose (maximum weight bearing through front leg) |
|  | WiiFit balance/ Dessert course | 160secs | The player is required to take steps on the balance board to move waiter to pick up a dessert from a chef and deliver the dessert to customers, while holding the game pad level to balance the dessert on the tray | Stepping on spot/ waiter moves proportional to speed of stepping, dessert falls off tray if game pad not held horizontal, allows for error | Nil/ leaderboard, points (increase number of desserts delivered) | Mocking, potential risk of falls/ requires fast step frequency in order to move at adequate speed, must coordinate UL/LL simultaneously | To decrease challenge, allow player to take slower steps, and aim to balance board (record number of dropped desserts in allocated time) |
|  | WiiFit balance/ Obstacle course | 80secs | The player is required to step on the balance board to move forward through a course and avoid obstacles in passing | Stepping on the spot/ character moving forward relative to player stepping speed, allows for error, if hit by an obstacle character gets pushed off | Progress to ‘Ultimate obstacle course” (see below)/ leaderboard, increasing distance walked in time | Requires fast stepping to avoid obstacles when passing them | Focus on stepping and speed of stepping rather than trying to avoid obstacles |
|  | WiiFit balance/ Ultimate obstacle course | 80secs | The player is required to step on the balance board to move forward, turn body and feet to change directions to avoid obstacles and follow course | Stepping on the spot/ character moving forward relative to player stepping speed, allows for error, if hit by an obstacle character gets pushed off | Nil/ leaderboard, increasing distance walked in time | Falls risk with turning/ requires fast stepping to avoid obstacles when passing them | Focus on stepping and speed of stepping rather than trying to avoid obstacles |
|  | WiiFit balance/ Scuba search | Game ends when oxygen tank runs out | The player is required to stand on balance board and step *or* squat to swim and simultaneously turn body (anywhere up to 360°) in order to collect fish | Stepping, squatting on the spot/ movement of scuba diver, total score of fish collected at end | Nil/ number of fish collected in time | Falls risk with turning 360°/ need good balance, must change from looking at screen to game board when facing backwards |  |
| **Standing** | WiiFit balance/ Bird’s-eye Bulls’ eye | ~60secs | The player is required to stand on the balance board and flap arms while shifting weight in all directions in order to make the bird fly to and land on targets | Flapping arms while standing on spot and weight shift in all directions/ height of bird, direction of movement | Nil/ leaderboard, increasing number of targets hit, decreasing time to target | Requires vigorous flapping with arms (additional aerobic demand), requires good shoulder ROM and nil shoulder pain | Aim at decreasing time to first target rather than trying to reach as many targets as possible |
|  | WiiFit balance/ Snowball fight | 90secs | The player is required to stand on balance board and laterally shift weight to avoid getting hit by snowballs and to position character to throw snowballs at targets by aiming with the Wii remote | ML direction, aim with upper limb/ allows for errors, character moves left or right from behind the screen, snowball hits target, strength (distance of throw) decreases if you get hit | Nil/ leaderboard, increase number of targets hit in allotted time | Aiming with UL requires finer movements and coordination between UL and LL, requires quick weight shift so as to avoid getting hit by snowballs | Set a small target number |
|  | WiiFit balance/Tilt city | ~90secs | The player is required to tilt Wii remote and laterally shift weight while standing on the balance board in order to place balls into buckets according to colour | ML direction (LL), tilting of remote (UL)/ allows for error, tilt of upper bar and lower bars, balls into buckets | Nil/ leaderboard, score of number of balls into buckets | Requires UL/LL coordination, increased cognitive demand with choosing the correct bucket to place balls into | Focus on the number of balls that the player is getting into the buckets |

## XBOX KINECT

### Getting started with Xbox Kinect

1. Place sensor at hip height, and ensure you have sufficient play space to play Xbox Kinect.
2. You need the Xbox remote to get started to initially navigate along the Xbox menu. Hold down the silver “X” button to turn the remote on. Use the “A”, “B”, “Y” and “X” buttons and arrows as instructed on the screen.
3. Turn Xbox and TV on without disc in the console.

### Creating an avatar in Xbox Kinect

1. As for the Wii you can create a personal profile for your participant and this will enable them to work on beating their best score and they may enjoy seeing their own personal avatar on the screen.
2. Use the remote to navigate to the Sign In menu. In here you can select a profile you have already created or you can create a new profile by naming a new profile, using an avatar as a base for your new profile and editing the features of the avatar to match your participant (if you want). Alternatively create a young and older male and female avatar and use these for all your participants.

### Playing a game with Xbox Kinect

1. Place the chosen software disc in console and it will go directly to that game. Note that you can create a new profile once you are in the game, you will need to sign out the current profile and then it will let you create a new profile.
2. Once the software is open, you no longer need the remote and you can use your hand to navigate through the software to the game you want to play.
3. If you need to pause a game at anytime, hold your left arm out 45° and hold it there until the game pauses.
4. The software we have investigated so far include: Kinect Adventures, Kinect Sports, Fruit Ninja (located in games library), Dance central, and your shape evolved 2012. Please note a lot of the Kinect games are very fast or require 2 arms to be used or the person to jump, so they are not appropriate for our participants. A lot are also not particularly specific to mobility limitations and don’t provide feedback regarding good movement patterns. However, the games are very engaging and fun to play, and may be more useful for our higher level participants to push their mobility limitations and to increase their overall physical activity.

| KINECT ADVENTURES | | | | | | | | | |
| --- | --- | --- | --- | --- | --- | --- | --- | --- | --- |
| **Mobility activity** | **Game** | | **Game length** | | **Description** | **Movement/ Feedback** | **Progress/ Motivation** | **Issues/ Additional demands** | **Modifications** |
| **Stepping and reaching in standing** | **Freeplay/ 20000 leaks (3 games leaks ahoy, go with the flow, ship shape)** | | ≥ 2mins | | The player is required to repair leaks infront, side and on floor by reaching and stepping and holding that position on leaks to plug them. | Step and reach in different directions/ score at end, bonus points finishing early | In game 3 waves, ↑duration and number of leaks at once, 3 games increase difficulty /, allows error | Timing, accuracy of hand and foot placement | Could just plug wall ones with hand and not step. |
| **Stepping in standing and jumping** | **Freeplay/ River rush (3 games curvy creek bouncing brook cozy cavern)** | | ≥ 2mins | | The player is required to make their way in a raft down a river by jumping to start, and stepping side to side to miss obstacles and to gain pins (which give points), and jumping to get high pins. | Jumping, side to side stepping/ score at end | No, different river courses for 3 games/ allows errors | Need to jump to start game, fast paced, timing to get pins | Pushing up on toes is perceived as jump by sensor, particularly fast up on toes. |
|  | **Freeplay/ Reflex ridge (3 games mover, collector, cruiser)** | | ≥ 2mins | | The player is on a railway track. They must collect pins (A) while avoiding obstacles by side stepping, ducking or jumping. | Jumping, side to side stepping, ducking/ score at end | No, different railway tracks for 3 games/ allows errors | Need to jump and duck to get over some obstacles, need to pull through with 2 hands to start the game. | Pushing up on toes is perceived as jump by sensor, particularly fast up on toes. |
| **Stepping in standing** | Freeplay/ Rally ball (3 games sure shot, treasure chest, peekaboo) | | ≥ 2mins | | The player is required to make their way in a raft down a river by jumping to start, and stepping side to side to miss obstacles and to gain pins (which give points), and jumping to get high pins. | Jumping, side to side stepping/ score at end | No, different river courses for 3 games/ allows errors | Need to jump to start game, fast paced, timing to get pins | Pushing up on toes is perceived as jump by sensor, particularly fast up on toes. |
|  | Freeplay/ Space pop (3 games Blast off, Halo hopper, Solar streaks) | | ≥ 2mins | | The player is in space in ship. They must pop all the bubbles by flapping both arms to make them go up in the air, and stepping side to side and forward and backward to get all the bubbles. | Stepping forward, backward and side to side while flapping arms. | No, 3 different games/ allows errors | Need both arms to flap to float up in the air | Nil |
| KINECT YOUR SHAPE FITNESS EVOLVE 2012 | | | | | | | | | |
| **Shifting weight in standing** | Warm-up/ juggle it |  | | Keep the soccer ball in the air with no hands by juggling it between your head, knees and foot | | Heading, kneeing or kicking virtual soccer ball/ | Unlock harder levels/ beating previous score |  | Nil |
|  | Warm-up/ kick it | 2 mins | | Kick the ball through goals | |  |  |  |  |
|  | Warm-up/ hurricane | 2 mins | | Stand and spin your arms together to create a hurricane of balls, 30secs then swap direction of arm spin | |  |  |  |  |
|  | Warm-up/ Pump it |  | | Shoulder abduction single arm or together, small range or large, Get points for the number of balls blown up and popped. | |  |  |  |  |
|  | Warm-up/ Hula la |  | |  | |  |  |  |  |
| **Maintaining a standing position** | Stack em up | 2mins | | Hold the virtual board in your hands, catch the falling blocks, stack them on the board, drop into the container on either side. To grow your board stand on 1 leg, hold more blocks on the board to get more points | | Standing with arms infront, move arms to empty board, single leg stance/ allows errors | Unlock harder levels/ beating previous score | Focus is on calories/ Dual task cognitive + UL task |  |
| **Stepping in standing** | Stomp it | 2mins | | Step forward or to either side | | Stepping forward and to sides/KOR colour lights up when get correct timing, score at end | Unlock harder levels/ beating previous score | Focus is on calories very fast paced and cognitively challenging, timing element/ Dual task cognitive | Modify by selecting just 1 colour only to step on e.g. step on purple if you want to practice stepping with left foot. |
| **Reaching in standing/ changing direction** | Wall breaker | 2mins | | Punch arms across body at 45 degrees to break cubes on the screen | | Rotating on the spot and punching side to side/ KOR block disappears when hit it, total score at end, allows error, tell you can do better | Unlock harder levels/ beating previous score | If you don’t punch hard enough it does not register, focus is on calories/ Dual task motor | Nil |
| **Physical activity/ Walking** | Run the world | ?? | | Follow course through New York city. | | Walking on the spot/ KOR gives time for distance walked | Unlock harder levels/ beating previous score | Speed not an issue, can stop and rest | Nil |
| KINECT SPORTS | | | | | | | | | |
| **Reaching while standing, stepping and Standing** | Mini-game soccer (super saver) |  | | The participant is required to reach and or step and reach side to side to stop the soccer ball | | Reaching side to side and stepping side to side/ KOR gives score, allows error | Progress to full soccer game/ beating previous score | Reasonably fast paced/ timing and dual task UL |  |
| KINECT DOWNLOADED GAMES | | | | | | | | | |
| **Maintaining a standing position** | Fruit Ninja |  | | The participant must maintain a standing position while cutting the fruit on the screen | | UL movements in standing position | Different games e.g. bomb included which you can’t hit/ previous score | Have to slice fruit fast enough to register | Use zen mode to begin as no bomb and allows you to miss fruit. |

## HUMAC 2013 (v.150)

Weight capacity = 150kg

### Getting started with HUMAC

1. Ensure balance board is plugged into computer.
2. Open the HUMAC software program on the desktop icon.
3. From the menu bar file option, select Preferences.
4. Under the General tab, ensure the interface section has “balance board” checked.
5. Under the Reporting tab, ensure metric units are selected.
6. Click the **OK** button to read the zero weight (ensure nothing is on the balance board). The HUMAC will return to the Main Menu.

### Adding a new participant into the HUMAC

1. From the main screen, click the patient button.
2. Select New patient button and add participant details. You have to enter DOB, height and weight.
3. After adding the participant information click OK to save new patient information and return to the main HUMAC screen.
4. The new patient name will now display in the top Title bar and all the buttons on the button bar to commence testing and exercise will be active.

### Commence exercising or testing

1. If you have already created your participant profile, select that patient using the patient button. Check that the participant you are working with is listed in the top title bar.
2. From the button bar select either “test” if you want to conduct an initial test or progress test, “exercise” to commence exercises with your participant or “dashboard” if you want to play any of the games e.g. pong (note: in dashboard mode the games are not played under a particular participant)

### Other features

1. The manual explains how to create test protocols, conduct reports to compare the same test protocol for the same participant over time, how to get usage reports (this details all the tests and exercises completed for each participant so will be good for process evaluation), how to back up data (this will be important).

### Participant setup on the board

1. Get the participant to stand on the board and when participant setup screen appears before each exercise, record the foot angle, position of medial malleolus and middle of heel using the letters and numbers displayed on the board (see page 56 manual)
2. Next set anatomical zero for participant. Ask the participant to stand in as neutral position and select OK. Whatever position they are in when you select OK this will be taken as neutral position.

|  | HUMAC | | | | | |
| --- | --- | --- | --- | --- | --- | --- |
| **Mobility activity** | **Game** | **Game Length** | **Description** | **Movement / feedback** | **Progress/ Motivation** | **Issues/ Modifications** |
| **Maintaining a standing position** | **Clinical Test of Sensory Organization (CTSIB) eyes open, firm surface** | Select: 15, 30, 45, or 60secs, OR 1, 1:30, 2, 5, or 10 minutes | Maintain EWB position to enable the ball to stay central on the target | Maintain EWB/ KOP keep ball on target or changes colour, graph KOR | Increase time, add foam or balance board or eyes closed; progress onto different game/ graph 2 games on different days to show improvement (test mode only) otherwise print graphs for 2 different days |  |
|  | **Weight Bearing** | Sets: 1,2,3,4,5,10, 15,20, 25  Rest b/n sets: 15, 30,45,60secs  Duration: 10, 15, 30, 45secs, OR 1, 1:30, 2, 5, or  10mins | The participant needs to maintain COP in neutral position in either AP or ML direction | Maintain COP in neutral position in either AP or ML direction/ KOP graphical display with written percentages of WB, KOR: report graph error | Duration and sets / KOP within game, KOR: report graph error |  |
|  | **Weight Bearing XY** | Sets: 1,2,3,4,5,10, 15,20, 25  Rest b/n sets: 15, 30,45,60secs  Duration: 10, 15, 30, 45secs, OR 1, 1:30, 2, 5, or  10mins | The participant needs to maintain COP in neutral position in both AP or ML direction | Maintain COP in neutral position in both AP or ML direction/ KOP graphical display with written percentages of WB, KOR: report graph error | Duration and sets / KOP within game, KOR: report graph error |  |
| **Mobility activity** | **Game** | **Game Length** | **Description** | **Movement / feedback** | **Progress/ Motivation** | **Issues/ Modifications** |
|  | **Centre of Pressure** | Sets: 1,2,3,4,5,10, 15,20, 25  Rest b/n sets: 15, 30,45,60secs  Duration: 10, 15, 30, 45secs, OR 1, 1:30, 2, 5, or  10mins | The goal is for the participant to keep the magenta cursor in the middle of the bulls eye by maintaining their COP in the neutral position | Maintain COP in neutral position/ KOP can see on grid where magenta cursor, therefore COP is located, KOR: graph with stability score and percentage of time spent in each quadrant and ring | Increase duration and sets, reduce rest time between sets/ KOP within game, KOR graph, compare between sessions. | Most useful for person with increased postural sway, may use as measurement to track progress. |
| **Shifting weight in standing**  **Shifting weight in standing** | **Stability** | 1-25 sets, rest 0-2minutes between sets; hold at each number on the clock for 1-10 secs | Shift your weight clockwise around face of a clock starting at 12. Keep the target green at each point by maintaining your weight at each point (target yellow when not correct) | Full circle around your neutral position, how much movement depends on level set/ KOP: target is green when COP is in correct position, yellow when not; KOR: % of time on target for each position, can graph | Levels 2-8 represent how far you have to move your weight in the different directions/ graph 2 games on different days to show improvement (test mode only) otherwise print graphs for 2 different days | Shorter time e.g. 5secs have to move quite quickly between targets, 10sec probably good to begin. |
|  | **Mobility** | 1,2,3,4,5,10,15,20, 25 sets, rest 15,30, 45, 60 secs between sets; time for each set 10, 15, 30, 45secs, 1,1:30, 2, 5, 10mins | Shift your weight clockwise by holding your COP on the moving target that circles around your neutral position. | Full circle around your neutral position, how much movement depends on level set, how fast depends on time set/ KOP: target is green when COP is in correct position, yellow when not; KOR: % of time on target, can graph. | Levels 2-8 represent how far you have to move your weight in the different directions/ graph 2 games on different days to show improvement (test mode only) otherwise print graphs for 2 different days | Shorter time e.g. 5secs have to move quite quickly between targets, 30sec probably good to begin. |
|  | **Game** | **Game Length** | **Description** | **Movement / feedback** | **Progress/ Motivation** | **Issues/ Modifications** |
|  | **Stability envelope** | 1,2,3,4,5,10,15,20, 25 sets, rest 15,30, 45, 60 secs between sets | The arrow shows the direction the participant should lean ( 0°, 45°, 90°, 135°, 180° 225°, 270°, 315°). The magenta cursor shows the participant COP. Therapist clicks the mouse on cursor when the participant reaches their furthest distance at each angle. | As far as participant can move in the direction of 0°, 45°, 90°, 135°, 180° 225°, 270°, 315° from neutral position. Self-paced. | Nil progress, measurement/ graphs total area of movement, table of area for each direction moved. | May be more useful for measurement of improvement in maximal excursion of COP in all directions. |
|  | **Weight Shift** | Sets: 1,2,3,4,5,10, 15,20, 25  Rest b/n sets: 15, 30,45,60secs  Reps: 5, 10, 15, 25, 30, 40, 50  Self-paced | The goal is for the participant to move through the green marker in the correct direction while staying inside the magenta boundaries | ML direction (0° rotation)/ KOP cursor stays magenta if within set boundaries, red if out of boundaries, magenta cursor moves towards green line as you move COP, KOR graph trace of COP movements, Score number of reps within boundaries and time taken to complete set reps. | Increase sets and/or repetitions, decrease rest between sets, increase distance have to move, decrease boundaries have to work between/ KOP within game, KOR graph, compare between sessions. | Can set different distance targets for each direction, larger set boundaries easier for participant |
|  | **Game** | **Game Length** | **Description** | **Movement / feedback** | **Progress/ Motivation** | **Issues/ Modifications** |
| **Shifting weight in standing** | **Limits of Stability** | Sets: 1,2,3,4,5,10, 15,20, 25  Rest b/n sets: 15, 30,45,60secs  Hold time on each target: 1-60secs  Total duration: self paced | The goal is for the participant to move their magenta cursor to the flashing yellow and black target by shifting their COP. The targets are selected in random order and the participant must return to the centre target between each outer target. All 8 outer targets must be completed to finish the set. | Random movement of COP in 8 directions (0°, 45°, 90°, 135°, 180° 225°, 270°, 315° around neutral position) distance to move dependent on level set/ KOP target turns green when cursor is held on the correct target, turns yellow if move off target before the hold time is up, KOR graph of COP trace and table showing % score compared to normal for path taken to get to target and average time taken to get to target | Increase level 2-8 (distance to target), reps and hold time, decrease rest between sets/ KOP within game, KOR graph, compare between sessions. | Therapist can skip a target if participant is having difficulty getting that target. |
|  | **Targets** | As for limits of stability | As for limits of stability but tailored for the participant. You get the participant to move AP and ML as far as they can go and set these as their limits. You can then add targets around their neutral position within their limits. | Movement in different directions that the targets have been placed by the therapist, the order the targets are added to the screen are the order they are selected/ feedback as for Limits of stability | Increase distance therapist sets the targets at, reps and hold time, decrease rest between sets/ KOP within game, KOR graph, compare between sessions. | The program enables you to set limits of movement first so that you can target a particular area. This may be more suitable than limits of stability if person has significantly different distances they can move their COP in the different directions. |
|  | **Game** | **Game Length** | **Description** | **Movement / feedback** | **Progress/ Motivation** | **Issues/ Modifications** |
| **Shifting weight in standing** | **Random Motion** | Sets: 1,2,3,4,5,10, 15,20, 25  Rest b/n sets: 15, 30,45,60secs  Duration: 10, 15, 30, 45secs, OR 1, 1:30, 2, 5, or 10mins | The goal is for the participant to keep their cursor on the randomly moving target (speed of target is set between 1[slow] to 5 [fast]) | Movement of COP in different random directions, change of direction/ KOP target is green when your magenta cursor is on the target, yellow when your cursor is not on the target. KOR: graph of trajectory of COP, % time on target. | Increase speed, sets, duration of game, decrease rest between sets, set greater limits of movement/ KOP within game, KOR graph, compare between sessions. | Set limits of movement first, can target a particular area when setting limits. |
|  | **Roadway** | Sets: 1,2,3,4,5,10, 15,20, 25  Rest b/n sets: 15, 30,45,60secs  Reps: 5, 10, 15, 25, 30, 40, 50 | The goal is for the participant to keep the round cursor between the roadway boundaries by moving their COP | Can be set ML or AP/ KOP green cursor indicates within boundaries, red cursor out of boundaries, changing score dial % of time within boundaries, KOR graph displays COP vs. time overlaid on roadway and % of time on target for raising (to right or anteriorly-concentric) or lowering (to left or posteriorly- eccentrically) | Can change the profile of the roadway (steeper rise or fall meaning you have to change direction quickly), level 1 (easy) to 5 (hard), [accuracy set to 0], can increase sets, reps and decrease rest period/ KOP within game, KOR table and graph, compare between sessions. | This is quite challenging even level 1 and super slow profile as you need to work out and remember which direction you need to move. Easier/more intuitive when working in the AP direction. Increased cognitive demands/less intuitive when working in the ML direction of movement. May also use force mode to practice generating force through affected lower limb (through placing unaffected limb on block next to board). |
| **Mobility activity** | **Game** | **Game Length** | **Description** | **Movement / feedback** | **Progress/ Motivation** | **Issues/ Modifications** |
| **Standing up from a chair** | **Force vs. Time** | Sets: 1,2,3,4,5,10, 15,20, 25  Rest b/n sets: 15, 30,45,60secs  Reps: 5, 10, 15, 25, 30, 40, 50  Self-paced | Ask the participant to stand up as quickly as they can at the beginning of each repetition, stay standing for repetition, and then sit in rest period between repetitions. | Standing up and sitting down/ KOP force vs. time graph is displayed, slope of curve shows speed of extensor force generation; KOR: table average peak force % BW and average time to peak force. | Can modify duration of repetition (i.e. time to stand up, hold time 1-60secs), duration of rest between repetitions (i.e. time to sit down and prepare for next STS, relax time 1-300 secs), ?force threshold, increase number of sets & repetitions, decrease rest time between sets/ KOP within game, KOR table and graph, compare between sessions. | ?? unclear proper use of this exercise. STS seems to work well if wanting to work on someone’s speed of force generation. Can target weaker side by putting other foot on block next to board. |
| **Sitting balance** | **Scale** | Sets: 1,2,3,4,5,10, 15,20, 25  Rest b/n sets: 15, 30,45,60secs  Reps: 5, 10, 15, 25, 30, 40, 50  Self-paced | Can use this set up for anything you would use scales for feedback for 1 foot e.g. sitting balance pushing through heel, step stance shifting weight forward | Apply force through leg on board to increase marker on the scales/KOP analogue scale shows weight through board, keeps line for maximal weight for each repetition; KOR: table average peak force % BW and average time to peak force. | Can modify duration of repetition (i.e. time to load the leg 1-60secs), duration of rest between repetitions (i.e. time to load other leg, relax time 1-300 secs), ?force threshold, increase number of sets & repetitions, decrease rest time between sets/ KOP within game, KOR table and graph, compare between sessions. | ?? unclear proper use of this exercise. |
|  | HUMAC GAMES | | | | | |
| **Mobility activity** | **Game** | **Game Length** | **Description** | **Movement / feedback** | **Progress/ Motivation** | **Issues/ Modifications** |
| **Shifting weight in standing**  **Shifting weight in standing**  **Shifting weight in standing** | **PONG** | Choose number of balls per set (5,10,15,25,30 40, 50, unlimited), number of sets (1,2,3,4,5,10,15,20, 25) and rest between sets (10, 15, 30, 45,60secs).  Each ball lasts until you or computer miss it. | Hit the ball back with your paddle to your opponent (computer) by shifting your COP | Choose COP shifts ML or AP (set participants ROM)/ KOP paddle moves up and down as you shift your weight, KOR whether you miss the ball. | Compete against computer, however computer didn’t lose when I played so may lose motivation/KOP and KOR within game, keeps score between computer and participant and states at end of sets who won. | Timing element of task. Set range of motion not to participant’s limits otherwise hard to move paddle full length. Set ball speed & acceleration low (1-3), paddle (8)and ball size (3) high N.B. the ball speed gets faster as it is hit between the paddle, but therapist can slow down again. Can also set computer skill (0 beginner, 100 expert, at 50% computer still won each point). More useful when training AP movements. |
|  | **BREAKOUT** | As for Pong | Move the paddle to knock the blocks out of the wall by shifting COP. | Choose COP shifts ML or AP (set participants ROM)/ KOP paddle moves up and down as you shift your weight, KOR whether you miss the ball. | Can limit the number of balls. | Same issues as Pong. Better option when training ML movements. |
|  | **BALANCE** | Dependent on how long it takes to get the ball on target | Shift COP R/L and A/P to tilt the board to move the ball through a maze into the target hole. | Move COP R/L and A/P (set participant ROM) / KOP board on screen tilts in the direction you move, KOR get ball on target and game finishes, score is the time it takes to complete | Level 1-15/ Compete to beat each level best time | If slow movement then ball can get stuck on edges. Table tilt in WiiFit has better sensitivity. |
|  | **SKI** | 1-2min per level (depends how long to ski down the hill) | Ski race where you lean forward on the board to ski down the hill and shift COP to R and L to turn | Keep COP anterior and move R and L/ KOP see the skier turn as you shift weight R or L, score is the time it takes to complete | Level 1-12/ Compete to beat each level best time | Game doesn’t indicate if going in wrong direction. |
|  | **SNOWBOARD** | 1-2min per level (depends how long to board down the hill) | Same race as ski, but on a snow board. Lean L or R (board turned side on) on the board to ski down the hill and shift COP to AP to turn | Keep COP on front leg and move AP/ KOP see the boarder turn as you shift weight A or P, score is the time it takes to complete | Level 1-12/ Compete to beat each level best time | Game doesn’t indicate if going in wrong direction.  Good for loading affected leg as front leg (if wrong leg is used player won’t progress downhill). |
|  | **Game** | **Game Length** | **Description** | **Movement / feedback** | **Progress/ Motivation** | **Issues/ Modifications** |
|  | **LUGE** | 1-2min per level (depends how long to board down the hill) | Same race as ski, but on a luge. Lean forward on the board to move down the hill and shift COP to R and L to turn. | Keep COP anterior and move R and L/ KOP see the luge turn as you shift weight R or L, score is the time it takes to complete | Level 1-12/ Compete to beat each level best time | This game appears to still be under construction, can only play in demo mode (can choose upright or reclined, just what the person in the luge looks like) |
|  | **FLIGHT** | > 1min each level | Shift COP Anterior to make plane descend, Posterior to make ascend, L and R to turn. Steer the plan to hit targets. | AP and R/L / KOP see plane turn as shift weight, score is time taken to hit 3 targets. | Level 1-12/ Compete to beat each level best time | Can be difficult to get target if shifts weight quickly. |
|  | **ANIMAL ADVENTURE**  **(not available new software)** |  | Shift weight to place the ball on the appropriate target | Pt’s own pace | No feedback, gives score | Shifting weight towards all directions and maintain position for fixed seconds and tests pt’s cognitively visual and audibly |
|  | **PACMAN**  **(not available new software)** |  | Eat the pallets and don’t get captured by the ghosts | Quick reactions | No feedback, gives score | Shifting weight L)↔R), F)↔B) |
|  | **Game** | **Game Length** | **Description** | **Movement / feedback** | **Progress/ Motivation** | **Issues/ Modifications** |
|  | **EGG DROP**  **(not available new software)** |  | Catch the egg with the spoon by lining up the shadows on the floor | Quick reactions, fast pace | No feedback, gives score | Shifting weight towards all directions |
|  | **SPACE INVADERS**  **(not available new software)** |  | Avoid the missiles by shifting your weight | Quick reactions, (timer: 1,5 minutes, infinity and custom) | No feedback | Shifting weight L)↔R) |

## FYSIOGAMING 2015 (v2.1)

### Set-up

- Prior to launching the software ensure the sentinel key (blue USB stick) is plugged into the computer and the Kinect sensor is connected.
- Play space: ideally Fysiogaming requires 1.55m wide each side of the Kinect sensor, and 3.2m distance from the Kinect sensor, with the play area starting 1.3m from the Kinect sensor.
- Launch Fysiogaming software from the desktop icon. The home page has tabs across the top “Patient”, “Module”, “Exercise”, “Play” and “Results”. The software launches in the patient tab. The most recent player will be shown on the screen.
- Kinect sensor location: The Kinect sensor height should be correctly defined. Depending on the height of the patient you may wish to increase or decrease the sensor height. In the patient tab go to **“Settings”**. Measure the distance from the ground to the base (foot) of the Kinect. Type the number (height in cm) and click save. Click on **“Save”** to accept the changes.

### Adding a new participant

- Select “New patient” button in Patient tab.
- Enter participant data e.g. first name and initial for the surname (remember de-identified participant data for final storage of electronic and paper copies- you can go in and modify the participants data to be their participant ID once they have finished using the system.
- Note: *****means a required field.
- Enable capture patient photo unless the participant specifically says not and a picture will be taken of the participant from the Kinect sensor.
- Calibrate: select “every time” for the first session and then go in and change to “use previous calibration”.
- Breakdown: this allows you to customize rest periods between sets of exercises (exercises) and between different exercises (series).
- Save changes once you have completed this screen and that participant is now added to the system.

### Patient Tab

From the patient tab you can select your participant to commence exercising. If it is the participant you just entered then you are ready to go.

- Other buttons on the patient tab include:
  - Patient data: displays basic patient’s data and enables you to edit details about the selected participant.
  - **Statistics** – click here to view training session history and statistics of the selected patient.
  - **Details** – click to see the details of the last training session (see Figure 1)

### Starting to exercise

- On the right hand side of the patient tab there are 4 Options to starting a training session:

a) **New program** – click here to create a new training program.

b) **Reapply last** – click here to repeat the last training program.

c) **Increase level** – click here to repeat the last training program with the difficulty level increased by one.

d) **Adjust program** – click here to adjust the last training program.

- To create a new program you will be taken to the Exercise (or Module) Tab where you can navigate through different types of exercises (see table below) and select reps and sets of each exercise to create a total program. As you select each exercise the total time of exercise for the whole program is displayed at the top right hand corner.

### Difficulty level

Fysiogaming offers 30 difficulty levels which are divided into three groups:

- 1-10: Easy

*Performance*: Exercise can be performed with low precision.

*Speed*: The speed is very low.

*Games*: A required reaction time is high. Playing and gaining points is easy.

- 11-20: Medium

*Performance*: The application requires moderate precision in performing exercises.

*Speed*: The speed is on a medium level.

*Games*: Games are more demanding in terms of reflexes and reaction time than on levels 1-10.

- 21-30: Difficult

*Performance*: Exercises require high accuracy.

*Speed*: The speed is on a high level.

*Games*: Required reaction time is small. The games become more difficult and more challenging.

### Assessment Centre

- You can use this section to get a repeat measure on participants’ performance. You can assess:
  - Sit to stand: time for 1 repetition, time for 5 repetitions, number of repetitions in 30secs
  - Trunk balance Ax seated movement:
  - Trunk balance Ax standing movement:

### Reviewing and reporting participant data

- Within the statistics button on the patient tab you can review the session history.
  - Reports: You can save/print graphs of the participants movements during the games
  - Details: You can save/print exercises/games completed including program, reps, duration, score and difficulty level.
  - Re-apply: to start program again.

Adjust program: click here to adjust the last training program.

|  | FYSIOGAMING | | | | |
| --- | --- | --- | --- | --- | --- |
| **Mobility activity** | **Game** | **Description** | **Movement / feedback** | **Progress/ Motivation** | **Issues/ Modifications** |
| **Stepping in standing** | **Walking sideways** | The player steps to L and R to move the boat to catch the falling gems and to miss the barrels. | Side stepping to right and left/ KOR score at end, allows error | Game can be set from level 1 (easy) to level 30 (hard) which reflects accuracy/ score and level of game, medals and photos at end of session | Need sufficient room to step side to side/ timing and cognitive demands to get gems and miss barrels. |
|  | **Hip abduction** | The player moves their leg to the L and/or R to move the boat to catch the falling gems and miss the barrels. | Hip abduction to right and left/ KOR score at end, allows error. | Game can be customized to be set from level 1 to level 30 (increasing difficulty reflects accuracy/speed), ROM of legs (50% or 100%), limbs involved (left/right or both) and movement type (controlled VS. dynamic). Medals and photos at end of session | Difficult for therapist to provide standby/physical assistance as may get in way of movement.  May use for eccentric training of hip abductors in stance limb. |
|  | **Side strides** | The player must lunge to the L and R to lay down pillars to build a pergola. | Side lunges to the right and/or left/ KOR score at end, allows error | Game can be customized to be set from level 1 to level 30 (increasing difficulty reflects accuracy/speed), ROM of legs (50% or 100%), limbs involved (left/right or both) and movement type (controlled VS. dynamic). Medals and photos at end of session | Need sufficient room to step side to side. Difficult for therapist to provide standby/physical assistance as may get in way of movement. |
|  | **Lunges (forward)** | The player must lunge forward alternating between the L and R legs to row the boat down the stream | Forward lunges to the right and/or left. KOR score at end, allows error | Game can be customized to be set from level 1 to level 30 (increasing difficulty reflects accuracy/speed), ROM of legs (50% or 100%), limbs involved (left/right or both) and movement type (controlled VS. dynamic). Medals and photos at end of session | Need sufficient room to step forward. |
|  | **Knee and hip flexion** | The player must flex at the hip and knee to lay down pillars to build a coliseum. | Hip and knee flexion on the right and/or left. KOR score at end. Allows error. | Game can be customized to be set from level 1 to level 30 (increasing difficulty reflects accuracy/speed), ROM of legs (50% or 100%), limbs involved (left/right or both) and movement type (controlled VS. dynamic). Medals and photos at end of session | May use block to make into a step touch exercise. Increase block height/use of polystyrene cup to increase difficulty. |
|  | **Knee flexion (hip neutral)** | The player must flex at the knee with the hip in neutral position to lay down pillars to build a pergola. | Knee flexion with the hip in neutral position. R and/or L LLs may be used. KOR score at end. Allows error. | Game can be customized to be set from level 1 to level 30 (increasing difficulty reflects accuracy/speed), ROM of legs (50% or 100%), limbs involved (left/right or both) and movement type (controlled VS. dynamic). Medals and photos at end of session | Sensor may not always pick up movements posteriorly. |
|  | **Dynamic balance forward and backward/ Dynamic balance side to side/ Dynamic balance forward and to the sides/ Dynamic balance- X/ Dynamic balance-cross/ Dynamic balance-random** | A 9 cell grid is displayed on the screen. The player must step in the direction of the flashing cell on the grid (this direction will depend on which game you select) | Step in ML/ AP/ diagonal direction/ KOR score at end, allows error | Game can be set from level 1 (easy) to level 30 (hard) which reflects accuracy/ score and level of game, medals and photos at end of session | Need sufficient room to step in different directions |
|  | **Walk in place** | A walking track is displayed on the screen. The player is required to walk on the spot and step side to side to collect gems and miss holes in the ground | Walking on the spot, step side to side/ KOR score at end, allows error | Game can be set from level 1 (easy) to level 30 (hard) which reflects accuracy/ score and level of game, medals and photos at end of session | Need sufficient room to step in different directions |
| **Shifting weight in standing** | **Leaning trunk to the sides/ Leaning trunk forward and backward/ Leaning trunk forward, backward and to the sides/ leaning trunk to diagonal directions/ Leaning trunk to all sides** | A 9 cell grid is displayed on the screen. The player must move their COP in the direction of the flashing cell on the grid (this direction will depend on which game you select) | ML/ AP/ diagonal displacement of COP/ KOR score at end, allows error | Game can be set from level 1 (easy) to level 30 (hard) which reflects accuracy/ score and level of game, medals and photos at end of session | Game movement is more trunk LF, to promote movement at the hips, get the person to reach with their arm in the direction of the flashing cell. |
| **Reaching while standing** | **Reaching** | An ocean ground is displayed on the screen. The player is required to reach forward in different directions using R and L hand as instructed | Moving COP and reaching in direction of target/ KOR score at end, allows error | Game can be set from level 1 (easy) to level 30 (hard) which reflects accuracy/ score and level of game, medals and photos at end of session | Can tailor program for single arm use in hemiplegic patients |
| **Standing up from a chair** | **Sit to Stand** | An ocean ground is displayed on the screen. The player must stand up and sit down to move the submarine Up (standing up) and down (sitting down) to collect the coins | Standing up and sitting down/ KOR score at end, allows error | Game can be set from level 1 (easy) to level 30 (hard) which reflects accuracy/ score and level of game, medals and photos at end of session | The timing of the movement is not in sync with what is displayed on the screen. |

## STEPPING TILES

### Equipment

The stepping tiles system comes with 1 maintile (large tile), 6 bridging tiles (small tiles with orange connections in parallel), 6 subtiles (small tiles with 1 orange connection), and a cord to connect a subtile or bridging tile at a distance (e.g. stepping up to touch a block exercise in Table below)

### Getting started

Plug the main tile into the computer via USB cord and launch the stepping tile program.

Click Start and Full Screen on the top left hand corner (the boxes will be checked once this is done🗷)

### Overview of the screen

*Settings:* On the left hand side of the screen are the setting features where you can make adjustments as to the type of feedback and repetition goal for the participant.

*Live Feedback:* In the middle of the screen is the live feedback which the patient can use to see where they are distributing their pressure (shown as circles and/or percentages) and the count of their repetitions (if subtiles are attached).

*Results:* On the right hand side of the screen is the results section where the participant can see their progress towards their repetition goal and the participant data can be saved.

### Using the system with only the maintile

This set up is for exercises that only require the maintile e.g. shifting weight in standing. Note: the Step Counter and Pressure threshold from the settings section and the Results section do not work in this set-up.

*Settings:* Exercise: Maintile

Balance: There are 3 options:

The live feedback screen will display the participant’s centre of pressure from the 4 sensors displayed as 4 circles. This option could be used for participants either just learning to maintain a standing position or reaching in sitting (see Table below).

option 1:

%

The live feedback screen will display the participant’s centre of pressure from the 4 sensors displayed as 4 circles and 4%. This option again could be used as in option one, but used when more specific feedback is wanted to increase their percentage of body weight in a particular direction.

option 2:

%

%

%

%

%

%

The live feedback will display the participant’s centre of pressure from the 4 sensors displayed as 4 circles and 2% (sum of 2 sensors under the left foot and 2 sensors under the right foot). This option will be useful for shifting weight in standing exercise (see Table below).

option 3:

### Using the system with the maintile, bridging and subtiles

This set up is for stepping type exercises (or weight shifting exercises if you are not interested in %bodyweight and want repetitions counted). The Balance component from the settings section does not work in this set-up.

*Setting up the subtiles and bridging tiles:* You can configure the bridging and subtiles in any way to create your desired exercise (see Table below for ideas). Note: it is the subtile that the sensor works to count repetitions not the bridging tile, so configure them in a way that this is the tile that the person will place their centre of pressure.

*Settings:* Exercise: Overview

Balance: (not applicable)

Step Counter: select total number of repetitions you want the participant to complete in increments (or decrements) of 10 or 1(note if you want 30 steps with right foot and 30 steps with the left foot, add 60 as your step goal.

Pressure threshold: This allows you to adjust how sensitive the sensors are to picking up the centre of pressure through the subtiles to count a repetition. The smaller the % (as low as 1%) the more sensitive the sensors are- that is, the easier it is for the participant.

*Live Feedback:* The maintile displays percentages and the subtiles display a circle with repetition counts within the circles (see screenshot below).

*Results:* Goal: The repetition goal you selected under settings is displayed here and the number of repetitions the participant is up to is displayed as total steps and a % bar graph is displayed of the number of repetitions/total goal. When the goal is reached a text box appears congratulating the person for achieving their goal. You can then select “reset all countings” for the person to do another set of the same repetition goal.

Saving file: You can save the number of repetitions the person did (and add a note describing the exercise or tile set-up) and it will be saved as an excel file within the Liverpool GUI beta folder. Note: it only saved the repetitions just completed, so you need to hit save for each set of repetitions and combine excel files at the end for a total session count.

### Other tips for using the system

- When you connect the bridging and subtiles they should be displayed on the overview screen. If they are not displaying, this means that there is something wrong with the connection. Try giving the tile a bit of a wiggle or disconnect and re-connect.
- This system works as touchscreen on the Acer Aspire computers. The pressure threshold % can be slid in either direction.

| STEPPING TILES | | | | | |
| --- | --- | --- | --- | --- | --- |
| **Activity/ Exercise** | **Photo** | **Description** | **Movement / feedback** | **Progress/ Motivation** | **Issues/ Additional demands** |
| **SITTING BALANCE:**  **Loading the leg in sitting** |  | Position participant sitting on a chair with both feet on the main tile shoulder width apart. Practice pushing down through your heel of your left foot to increase the size of the left back dot.  (sensor setup: left back sensor)  (Repetition count: every time increase pressure through back sensor to threshold level) | Pushing down through the affected leg / KOP and KOR the dot on the screen gets bigger with pressure, fb from therapist | Increase the threshold, no. of reps, progress to reaching in sitting, sit to stand or standing/ increasing size of dot on screen | If very weak, can try to place small object directly on sensor under the heel so dot is visible for the participant |
| **LOADING LOWER LIMB:**  **Hip extension over the side of the bed** |  | Position participant lying on their back with one leg resting over the edge of a bed on the maintile. Ask the participant to practice pushing down through their foot and lifting their bottom off the bed, using the scales as feedback.  (sensor setup: left back sensor)  (Repetition count: every time increase pressure through back sensor to threshold level) | Hip extension/ KOP and KOR the footprint on the screen gets bigger with pressure, fb from therapist | Increase the threshold, no. of reps, range of movement, progress to standing/ increasing size of dot on screen | If very weak, can try to place small object directly on sensor under the heel so dot is visible for the participant or provide MG at knee to load the leg |
| **Activity/ Exercise** | **Photo** | **Description** | **Movement / feedback** | **Progress/ Motivation** | **Issues/ Additional demands** |
| **REACHING IN SITTING** |  | Position the participant sitting on a chair with both feet on the maintile shoulder width apart. Practice reaching to your affected side remembering to push down through your left leg.  (sensor setup: left front+back combined)  (Repetition count: every time increase pressure through left sensors combined to threshold level) | Reaching in sitting, loading leg in direction of reach/ KOP and KOR the footprint on the screen gets bigger with pressure, fb from therapist | Increase the threshold, no. of reps, reach distance, decrease thigh support, change nature of task, progress to sit to stand/ increasing size of dot on screen |  |
| **STANDING UP** |  | Position yourself sitting with your thighs half supported on a raised plinth, your feet on the maintile shoulder width apart and your knees bent so that your toes are under your knees. Practice standing up with __% through your left foot.  (sensor setup: left front+back combined)  (Repetition count: every time increase pressure through left + right sensor to threshold level) | Sit to stand/ KOP and KOR the footprints on the screen gets bigger with pressure, repetition count when sufficient WB through left leg, fb from therapist | Increase the threshold, no. of reps, reach distance, decrease thigh support, change nature of task, progress to sit to stand/ increasing size of dot on screen |  |
| **Activity/ Exercise** | **Photo** | **Description** | **Movement / feedback** | **Progress/ Motivation** | **Issues/ Additional demands** |
| **MAINTAINING A STANDING POSITION:**  **Standing equal weight bearing** |  | Position yourself standing on the maintile with your feet shoulder width apart. Stand with 50% of your bodyweight through each foot for – minutes.  (sensor setup: front + back combined for both)  (Repetition count: set time for task e.g. 30secs) | Maintaining COP in neutral position/ KOP and KOR the footprints on the screen gets bigger with pressure, repetition count when sufficient WB through left leg, fb from therapist |  |  |
| **SHIFTING WEIGHT IN STANDING** |  | Position the participant standing on the maintile with their feet shoulder width apart. Instruct participant to shift > --% of their bodyweight through their left foot – repetitions.  (sensor setup: front + back combined for both).  (Repetition count: every time increase pressure through left + right sensor to threshold level) | ML movement COP/ KOP and KOR the footprints on the screen gets bigger with pressure, repetition count when sufficient WB through left leg, fb from therapist |  |  |
| **Activity/ Exercise** | **Photo** | **Description** | **Movement / feedback** | **Progress/ Motivation** | **Issues/ Additional demands** |
| **STEPPING IN STANDING:**  **Stepping grid exercise** |  | In this set up, you will need 1 main tile and 4-6 sub-tiles (the picture shows 4 subtiles connected) and 4-6 bridging tiles if you want the arc further away.  Connect the subtiles to the sides and front of the main tile. They should appear on the screen as above}  Position yourself standing on the maintile with your feet shoulder width apart. Starting with your right foot, step to touch the subtiles working right to left. Repeat with left foot.  Sensor setup: 1 sensor each subtile  Repetition count: every time foot steps back on maintile |  |  |  |
| **Activity/ Exercise** | **Photo** | **Description** | **Movement / feedback** | **Progress/ Motivation** | **Issues/ Additional demands** |
| **STEPPING IN STANDING:**  **Stepping exercise** |  | Use maintile with 1 or 2 subtiles and 1 or 2 bridging tiles in whichever direction you want the person to practice stepping. You can draw a footprint or use non slip mat footprints to indicate where you want the person to step. Note depending how far the person is able to step you can order the subtile and bridging tile. That is, for a small step, attach subtile to maintile and add bridging tile to end of subtile incase they do step further than anticipated. For someone practicing larger steps, swap the position of the subtile and bridging tile  Position yourself standing on the maintile with your feet shoulder width apart. Step forward and shift your weight onto your left foot to land on the footprint. Step back. Repeat ____ times.  Sensor setup: maintile front + back combined each side, subtile 1 sensor)  Repetition count: every time foot steps back on maintile or every time touches subtile. |  |  |  |
| **Activity/ Exercise** | **Photo** | **Description** | **Movement / feedback** | **Progress/ Motivation** | **Issues/ Additional demands** |
| **REACHING AND STEPPING IN STANDING:**  **Stepping up to touch a block** |  | {maintile on ground, subtile connected by cord, placed on block in front of maintile}  Position yourself standing on the maintile with your feet shoulder width apart. Shift your weight onto your right leg and step up to touch the subtile with your left foot. Step your left foot back down onto the maintile. Repeat------ times.  (Repetition count: every time foot steps back on maintile or every time touches subtile) |  |  |  |
| **STEPPING UP AND DOWN ON A BLOCK** |  | {maintile on ground, subtile connected by cord, placed on block in front of maintile}  Position yourself standing on the maintile with your feet shoulder width apart. Step your left and right feet up onto the block in front of you. Step back down onto the maintile. Repeat------ times.  (Repetition count: every time foot steps back on maintile or every time touches subtile) |  |  |  |

## AMOUNT APP

### Getting started

- Download FileMaker Go via this link:

[https://itunes.apple.com/us/app/filemaker-go-13/id675292600?mt=8](https://webmail.thegeorgeinstitute.org/owa/redir.aspx?C=1ed2b13e9c694094922cb13930d6257e&URL=https%3a%2f%2fitunes.apple.com%2fus%2fapp%2ffilemaker-go-13%2fid675292600%3fmt%3d8)

- Download AMOUNT App latest version (0.99) via this link:
  XXXXXXXXXXXXXXXXXXXXXXXXXXXXXXXXXXXXXXXXXXXXXXXXXXXXXXX
- To download the AMOUNT App icon you need to have an email account set up on the iPad. Next email Leanne at: [XXXXXXXXXXXXXXXXXXXXX](mailto:lhassett@georgeinstitute.org.au) and she will send you an email with an attachment which you will need to click on (when viewing this email on your iPad) and follow the prompt to install this. It will then create an icon on your iPad home screen to go directly into the AMOUNT FileMaker Go App. Note: the filemaker go icon still needs to stay on the iPad, but the patient should be taught to go in through the AMOUNT App icon.

### Using the AMOUNT App

There are 3 modes that you can work in:

- Administrator: this is where you add your therapist details.
- Therapist mode: this is where you add a participant and create a program for them.
- Patient mode: This is what the participant sees.

To move between the different modes you may need a pin (Admin Mode: xxxxxx; Patient Mode: xxxxxx; Therapist Mode: xxxxxx. When you give it to a participant you will set it in patient mode and they will be unable to move into the other 2 modes.

### Working in Administrator mode

In administrator mode there are two tabs, home and setup. Home displays the AMOUNT logo and current version of the App. The setup tab shows the therapists that have been entered into the App for that iPad.

To add a new therapist:

- Hit the plus button at the top of the screen. Add the therapist name and email address (phone number if you want).
- If you only need to add one therapist you can then hit the “activate therapist mode for this therapist” button and you will be taken into therapist mode.
- In admin mode if you select one of the therapists that have been entered into the App, it will show you their details and the names of the participants that have been entered under this therapist (see screenshot below- Note you cannot see the participant program from this tab).

### Working in therapist mode

In Therapist mode there are three tabs; home, patients and programs.

- The home tab shows the therapist details at the top and the list of participants that have been created for that therapist. You can select any of the participant names already entered to go into their current program to edit.
- The patient tab shows the list of current participants already entered under that therapist and again you can go into the participant’s current program to edit from this tab.
- The program tab shows you the 15 programs that have been created in the AMOUNT App and you can select any of these programs to review the exercises included.

To add a new participant go to home tab and press “+Create New Patient” button at bottom right hand side of the screen. You will be taken to a screen where you add the participant details and program time period on the left, and develop the exercise program on the right.

- Enter participant details (no fields are mandatory, first name or first name and surname initial would be fine, you don’t need an email address, the App works from a gmail account already set up) and set the dates that you want the program to run e.g. 1 month (a calendar appears to scroll through to make this easier to set).
- To add exercises press “create new patient program” button on the bottom right hand side and the 15 AMOUNT programs will appear. Add which programs you want to select exercises from (Note: you can press “load standard program” button on the top right hand side but you will need to do more deleting of programs and exercises this way.
- Under “Programs for this Patient” go into each program and edit. Edit can include deleting exercises you don’t want (press “X” button and “delete”), changing the number of repetitions/sets/duration/text describing exercise (note: the progression and variations box will not display in patient mode, this is there to give the therapist ideas how to vary and progress the exercise; you will need to write any of this information in the Client’s Instructions box if you want the participant to see it). You can also take a photo (by clicking on the default photo) or video (by clicking on the triangle next to the photo) of the participant completing the exercise and the default photo will be replaced by this photo and a video will be available for the participant to see.

- Once you have completed creating the program you can email a PDF copy of it to yourself to the therapist email address you put into the App.
- The final step to make the program ready for the participant is to schedule the exercises within the timeframe that you selected the program to run e.g. 1 month. A calendar should appear and you will be able to select which days within the scheduled time period that you want the participant to complete the exercises. For example if you created 10 exercises for the participant, you may select 5 to be completed each week on Mon, Wed and Fri and 5 to be completed on Tues, Thurs and Sat.
- Once you have finished creating the program and it is scheduled, you can then activate it into patient mode ready for the participant. This is done by pressing the “Activate Patient Mode for this Patient” button at the bottom of the participant details on the Patient Tab.

### Working in Patient mode

In the patient mode there are also three tabs; Introduction, My Program, and My Progress

- The introduction tab shows the AMOUNT logo and has two PDFs, one which provides information about the study “Aims of AMOUNT Study” and one that provides information about safety when completing the exercises “Exercise Safety”.
- The My Program tab shows the exercises that have been scheduled by the therapist to be completed for that day.
- The My Progress tab shows a graph of their percentage of completed exercises.

For the participant to complete their exercises:

- Go to the My Program tab. Check that the correct day is displaying at the top. On the left hand side will be the list of exercises that have been scheduled to be completed for that day.
- Select which exercise they would like to complete and click on it from that list. That exercise will now be displayed on the right hand side (Note: if there is a video attached to that exercise an arrow will be displayed next to the photo).
- The participant will then need to complete the exercise. Note if they did more or less sets/repetitions/duration than prescribed by the therapist, they can press on these numbers and edit them. Once they have completed the exercise they press done. That exercise will now disappear from the list on the left hand side and should move onto the next exercise. Repeat as described above for each exercise until no exercises are left on the left hand side (or until the participant can do no more). They should only press done when they have completed an exercise.

For the therapist to receive data from the participant:

- An envelope is located at the top right hand corner of the screen. This is to send details about the completed exercises to the therapist. This appears as an excel spreadsheet emailed to the therapist’s email address and it is a cumulative file for that program, that is, whenever they send their data, it will send the data for all the exercises completed within that scheduled program timeframe. You can either teach the participant to send it every day or just once a week or you can remind them when you have your weekly session.

### Other information

- To edit a program the Research Physiotherapist will need to physically update the program on the iPad. This will require a home visit. You would be able to increase the repetitions for the participant over the phone (by asking them to edit how many repetitions they do as they complete each exercise) or you could initially set up some harder exercises that you don’t schedule for the first period of time e.g. 1 month, but this may still need to be modified if they don’t progress as you envisage.
- The App works without internet access, however you will not be able to receive any data via email if they don’t have internet access. A SIM card can be used to provide internet access.
- On the top right hand corner on the Introduction page in Patient mode is a little cog wheel. This is to change to Administrator or Therapist mode and it will require a password.

| IPad AMOUNT APP | | |
| --- | --- | --- |
| **Program** | **Exercises** | **Modifications/ feedback** |
| **Preparation for standing up** | 1. Flexing the hips in sitting and sliding arms forward on a table to a target 2. Bending the knee in sitting 3. Pushing down through the leg in sitting 4. Sitting and reaching quickly 5. Sitting and reaching to the affected side 6. Standing up and sitting down from a high surface | Edit or replace exercises, take video of participant doing exercise/ exercise completion sent weekly to therapist, fb by therapist over phone, graphs. |
| **Standing up** | 1. Sitting forward 2. Moving the shoulders forwards in sitting to a target 3. Reaching to the side when standing up 4. Standing up and sitting down to a knee cue 5. Standing up and sitting down with hand support nearby 6. Standing up and sitting down from a dining table 7. Standing up and sitting down when holding an object 8. Standing up with one leg on a block 9. Standing up and walking | Edit or replace exercises, take video of participant doing exercise/ exercise completion sent weekly to therapist, fb by therapist over phone, graphs. |
| **Maintaining a standing position** | 1. Hip extension in standing against a wall 2. Bilateral squat to a target in standing 3. Standing and leaning forwards and backwards 4. Transferring weight laterally in standing against two walls 5. Standing and turning with feet close together 6. Standing in tandem stance 7. Standing on one leg with support | Edit or replace exercises, take video of participant doing exercise/ exercise completion sent weekly to therapist, fb by therapist over phone, graphs. |
| **Reaching in standing** | 1. Reaching from side to side in standing 2. Standing and reaching 3. Standing and reaching to the side 4. Standing and reaching from the floor to above the head 5. Stepping forward to reach for an object | Edit or replace exercises, take video of participant doing exercise/ exercise completion sent weekly to therapist, fb by therapist over phone, graphs. |
| **Stepping in standing** | 1. Marching on the spot 2. Maintaining single leg stance while touching the other foot on a block 3. Stepping 4. Stepping forwards over an obstacle with hand support nearby 5. Stepping forward to reach for an object 6. Stepping sideways against a wall 7. Stepping to targets with hand support nearby 8. Maintaining single leg stance while stepping backwards with the other leg | Edit or replace exercises, take video of participant doing exercise/ exercise completion sent weekly to therapist, fb by therapist over phone, graphs. |
| **Changing directions while walking** | 1. Stepping sideways against a wall 2. Stepping to targets with hand support nearby 3. Swivelling on the balls of the feet 4. Turning around on the spot 5. Walking in a figure-of-eight 6. Standing up and walking 7. Rolling the foot on a ball while standing 8. Walking and dribbling a ball around objects | Edit or replace exercises, take video of participant doing exercise/ exercise completion sent weekly to therapist, fb by therapist over phone, graphs. |
| **Climbing stairs** | 1. Raising the leg onto a block 2. Stepping up onto a block 3. Maintaining single-leg-support while stepping up and down on a stairs 4. Stepping down from a block to a knee cue 5. Moving the leg back and forth while stepping 6. Walking up stairs 7. Walking down stairs | Edit or replace exercises, take video of participant doing exercise/ exercise completion sent weekly to therapist, fb by therapist over phone, graphs. |
| **Physical activity throughout the day** | 1. Walking a set distance within a set time 2. Marching on the spot 3. Walking on slopes 4. Walking on uneven ground 5. Standing up and walking | Edit or replace exercises, take video of participant doing exercise/ exercise completion sent weekly to therapist, fb by therapist over phone, graphs. |
| **Running, hopping and jumping** | 1. Jumping forwards over lines 2. Jumping off a low object 3. Leaping from a standing start and landing on one leg 4. Leaping sideways 5. Hopping on the spot 6. Stepping onto a stool off tip toes 7. Swinging the foot quickly up to a target 8. Running between lines | Edit or replace exercises, take video of participant doing exercise/ exercise completion sent weekly to therapist, fb by therapist over phone, graphs. |
| **Low difficulty mobility** | 1. Ankle dorsiflexor strengthening in sitting without weights 2. Ankle plantarflexor strengthening in sitting without weights 3. Bending the knee in sitting 4. Hip flexor strengthening in sitting 5. Single leg hip extensor strengthening in supine 6. Hip abductor strengthening in sidelying without weights 7. Bilateral squat in standing 8. Standing with the feet together 9. Standing in semi-tandem stance 10. Reaching while seated 11. Standing up and sitting down from a high surface | Edit or replace exercises, take video of participant doing exercise/ exercise completion sent weekly to therapist, fb by therapist over phone, graphs. |
| **Medium difficulty mobility** | 1. Knee control in standing 2. Lifting an object from the floor to the table 3. Standing up and sitting down with hand support nearby 4. Hip abductor strengthening in standing against a wall 5. Maintaining single-leg stance while stepping backwards with the other leg 6. Maintaining single-leg stance while touching the other foot on a block 7. Moving from the heels to the toes in standing 8. Standing and picking up an object off the floor 9. Standing and turning with one leg forward 10. Stepping forwards to reach for an object 11. Stepping to targets with hand support nearby | Edit or replace exercises, take video of participant doing exercise/ exercise completion sent weekly to therapist, fb by therapist over phone, graphs. |
| **High difficulty mobility** | 1. Getting up from the floor 2. Standing and picking up an object off the floor with both hands 3. Standing up and sitting down from a low stool 4. Lowering and raising from a block 5. Raising and lowering from a block 6. Single leg heel raise 7. Maintaining single leg stance while rolling a ball around an obstacle 8. Braiding 9. Walking a set distance within a set time 10. Walking backwards 11. Walking on slopes 12. Walking up a kerb 13. Walking while performing multiple tasks | Edit or replace exercises, take video of participant doing exercise/ exercise completion sent weekly to therapist, fb by therapist over phone, graphs. |
| **Skill** | 172 exercises to choose from |  |
| **Strength** | 69 exercises to choose from |  |
| **Stretch** | 11 exercises to choose from |  |

## T-REX APP

Opening website with internet explorer 8 (more functionality with internet explorer 10 or Google chrome)

- T-Rex can be found at <http://XXXXXXXXX>
- Click the Start Button to go to the log in page
- Input your Username and Password (which will be sent to your email by the system manager – contact XXXXXXXXXX if you want access to site) and tick both the “I do agree to Terms and Conditions” and the “Remember Me” boxes
- The next screen is called Exercise Plans
- To view available videos, click exercise gallery
- Find patient and click ‘Edit”
- Next screen is called Edit Exercise plan
- If blank exercise is not visible, click on number to left of exercise box to expand
- To Add New Exercises to plan (see below)

1. click plus sign to the right of the screen

2. type the name into the search box

3. click on the plus sign on the exercise description or double click the picture

4. click on Edit details – this will auto-populate the Exercise Label

5. click on Edit details again to add frequency / sets and edit the instructions (unnecessary step if opened app in IE10 or Google chrome)

6. add another exercise box by clicking on the plus sign to the right of the screen and repeat the process

7. when completed scroll to top of page and click on Save Exercise Plan

**Then click update – this will allow the patient to see the exercise plan on their iPad**

- To view what the patient will see on their i-pad, click view exercise plan

| IPad T-REX APP | | | |
| --- | --- | --- | --- |
| **Program** | **Exercises** | **Description** |  |
| **Exercises in lying** | **Lie-to-sit** | Lying on back. Bend both knees and place feet on bed. Shift bottom across bed, away from side. Roll towards side of bed. Move feet over edge of bed and push up into sitting. |  |
|  | **Sit-to-lie** | Sitting on edge of bed, near head of bed. Using arm for support, lower self on to side and bring feet up on to bed. Roll on to back. |  |
|  | **Hip Flexion** | Lie on your back with your head on a pillow. Bend your hip and knee by sliding your heel towards your bottom. Slide heel back down to straighten leg. |  |
|  | **Straight leg raise** | Lie on your back with your head on a pillow. Tighten your thigh muscle, pull your toes up towards you and lift your heel up off the bed. Hold for a few seconds and lower, keeping your knee straight at all times. |  |
|  | **Inner Range quads** | Lie on your back with your head on a pillow. Place rolled-up towel under one knee. Pull toes back towards you and lift heel off bed. Keep the back of your knee on the towel at all times. |  |
|  | **Bridging** | Lie on your back with your head on a pillow. Bend both knees and place feet flat on bed. Tuck bottom under and lift your bottom up as far as you can. Hold for 3 seconds then lower your bottom. Lie on back. |  |
|  | **Ankle pumps** | Bend and straighten ankles briskly. |  |
|  | **Pelvic Tilting** | Lie on your back with your head on a pillow. Bend both knees and place feet flat on bed. Slowly press lower back on to bed and then arch. |  |
|  | **Lumbar rotations** | Lie on your back with your head on a pillow. Bend both knees and place feet flat on bed. Rock both knees over to one side as far as possible. Return to midline. Repeat to other side. |  |
|  | **Hip Abduction (supine)** | Lie on your back with your head on a pillow. Keep your knee straight and your toes pointing towards the ceiling. Slide your heel out to side as far as you can and then back to the middle. |  |
|  | **Hip Abduction**  **(side lying)** | Lie on your side with your lower knee bent and your top leg straight, in line with your body. Lift your leg up until it is parallel with the bed. Hold for 3 secs and then lower, keeping knee straight at all times. |  |
|  | **Static quads** | Lie on your back with your head on a pillow. Straighten your knee by tightening thigh muscle and pressing back of knee on to the bed. Hold for 3 seconds. Relax. |  |
|  | **Static Gluts** | Lie on your back with legs out straight. Squeeze your buttocks together. Hold 3 seconds. Release. |  |
|  | **Long sitting** | Sit with legs out in front of you. |  |
| **Exercises in sitting** | **Quads** | Sit upright with thighs horizontal; straighten your knee so that leg is parallel with ground. Hold position and return foot to ground. | a) add ankle weight |
|  | **Foot and ankle exercises** | a) lift toes and then lift heels  b) hold foot off floor and draw a circle with your toes | clockwise, then anticlockwise |
|  | **Hip Flexion** | Sit upright. Lift knee upwards as far as comfortable |  |
|  | **Sit to Stand** | Sit on chair with arm support (height of chair should ensure thighs are parallel to ground or hips slightly higher than knees) Place feet flat on floor, slightly behind knees. Lean forward to bring ‘nose over toes’. | a) use both hands  b) use one hand  c) cross hands over chest  d) place one foot in front of other.  e) with walking frame |
|  | **Biceps** | Sit upright. Start with elbow straight and hand hanging down towards floor. Bend elbow and bring hand to shoulder. Lower hand to straighten elbow. | a) add weight |
|  | **Triceps** | Sit upright. Bring the arm to be exercised up with the elbow pointing to the ceiling. Support the elbow with the other hand. Straighten the arm. | a) add weight |
|  | **Shoulder Flexion (active)** | Start with hand at shoulder and elbow at side. Push hand up towards ceiling straightening elbow. | a) add weight |
|  | **Shoulder Flexion (assisted)** | Link finger together. With help of stronger arm, lift both arms up together as far as possible. | a) use walking stick |
|  | **Shoulder external rotation** | Elbow at 90 degrees. Keeping elbow at waist, take hand out away from body and beside body. | a) in side lying  b) add theraband in sitting – bilateral/unilateral |
|  | **Shoulder internal rotation** | As above, elbow at side, hand out away from body, pull hand back in across stomach | a) add theraband |
|  | **Neck rotation** | Sitting upright with back away from chair. Turn head gently and slowly so chin moves towards shoulder. Repeat in other direction. |  |
|  | **Chin in** | Sitting upright with back away from chair. Press chin gently in, guiding head straight back. |  |
| **Standing / Balance exercises - stand up facing the back of a chair, firm surface or rail. Hold onto the back of the chair**  **Standing / Balance exercises - stand up facing the back of a chair, firm surface or rail. Hold onto the back of the chair** | **Hip flexion** | Stand up tall with feet comfortably apart. Hold on to support if necessary. Lift knee towards ceiling as far as possible. Repeat. | a) add weight |
|  | **Hip abduction** | Stand up tall with feet comfortably apart. Hold on to support if necessary. Keeping knee straight and toes pointing forward, lift leg out to the side. | a) add weight |
|  | **Hip Extension** | Stand up tall with feet comfortably apart. Hold on to support if necessary. Keeping knee straight and toes pointing forward, lift leg out behind you. | a) add weight |
|  | **Small knee bends / 1/4 squat** | Stand up tall with feet comfortably apart. Hold on to support if necessary. Bend both knees a little as if you are going to sit down. Straighten up again. | a) lower further towards ground as able |
|  | **Knee flexion** | Bend knee bringing foot up towards bottom. | a) add weight |
|  | **Back Extension** | Place hands in small of back, gently arch back. |  |
|  | **Trunk rotation** | Place hands on hips, with elbows pointing out to side. Turn as far as you can to one side and then the other without moving hips. |  |
|  | **Shoulder/Pendular exercises** | Bend at hips and lean with elbow on to table. Let other arm relax straight out towards floor. Gently let arm swing backwards and forwards or side to side. Draw circles. |  |
|  | **Weight Transference** | Stand up tall with feet shoulder width apart. Hold on to support if necessary. Shift weight over one foot, then the other. | a) both hands  b) one hand  c) no hands |
|  | **Standing balance – feet apart** | Stand up tall with feet shoulder width apart. Hold position. | a) both hands  b) one hand  c) no hands  d) eyes closed  e) slowly turn head from side to side |
|  | **Standing balance – feet together** | Stand up tall with feet together. Hold position. | a) both hands  b) one hand  c) no hands  d) eyes closed  e) slowly turn head from side to side |
|  | **Standing balance - tandem** | Stand up tall with one foot in front of the other as if taking a step. Hold position. | a) both hands  b) one hand  c) no hands  d) eyes closed  e) slowly turn head from side to side |
|  | **Standing balance - SLS** | Stand on one foot with other foot off the ground. Hold position. | a) both hands  b) one hand  c) no hands  d) eyes closed  e) slowly turn head from side to side |
|  | **Standing balance** | stand on balance mat – as 6-10 above. |  |
|  | C**alf Stretch** | Stand up tall with one foot in front of the other as if taking a step. Bend front knee and lunge forward, keeping back knee straight, until stretch is felt in calf. Hold position. Repeat on other leg. |  |
|  | **Hamstrings Stretch** | Place heel of one foot out in front of you / on a small stool. Lean forward at hips until stretch is felt in back of the thigh. Hold position. Repeat on other leg. |  |
|  | **Calf Strength** | Stand up tall with feet shoulder width apart. Hold on to support if necessary. Raise up on to toes and lower down again. | a) both feet  b) one foot |
|  | D**orsiflexor strength** | Stand up tall with feet shoulder width apart. Hold on to support if necessary. Shift weight back over heels raising toes up off ground. |  |
|  | **Tib Ant** | Leaning back on wall, lift toes up and then bring body away from wall |  |
|  | **Walking** | Walking. | backwards/forwards/sideways/figure 8s/heel-toe/heels/toes/sliding feet/altered surfaces/obstacles |
|  | **Stepping** | Stand up tall in front of a step. Hold on to support if necessary. Step up on to step and down.  a) tap alternate feet |  |
|  | **Walking with stick** | Handle of stick should be at level of hip joint. Hold stick in hand opposite to affected/painful/weak leg. Take step with bad leg and at the same, place the stick down level with the heel. Step through with good leg and repeat. |  |
|  | **Steps with walking aid** | Place walking aid up on step. Place stronger leg up on step and then step up with other leg.  Going down step – place walking aid down. Place weaker leg down and then follow with other leg. |  |
|  | **Wobble board** | Stand up tall place one foot forwards and one back on the wobble board. Take care the board does not slide on the floor when stepping on or off – a non-slip mat under the board will reduce slipping. | a) try to balance without touching edge  b) rock board forwards and step off  c) rock board backwards and step off |
|  | **Balance Recovery - support, corner of walls, no support** | a) forward  b) sideways  c) backwards |  |
|  | **Vestibular** | a) point to point eye movement with head still  b) head movements with eyes fixed on point  c) tracking object by moving head and eyes |  |

## FITBIT

Prepare a Fitbit account for the participant using a newly created email address and pair the Fitbit device to this account.

### Installing the Fitbit on your computer

<https://www.fitbit.com/au> | Get started (top right of screen) | Download the software for the “One” or “Zip”. The software will install a folder called “Fitbit connect” on your computer.

### To pair your Fitbit device with the online Fitbit account

**Instructions – use of the Fitbit**

Plug the dongle into the USB port of your computer.

Go to the start menu of your computer | All programs | Fitbit Connect folder | Fitbit Connect program.

Select: Set up a new Fitbit device | Existing user. Then enter your details, and **ensure you select the correct time zone (GMT+10:00 – Sydney)**, then follow all subsequent instructions.

### Wearing the Fitbit

Wear it on your waistband. Females may clip it onto their bra.

Wear it during the day. Take it off at night. Do not wear it in the shower or when you go swimming.

**NOTE: For participants walking slower than 0.8m/s, it is more accurate at counting steps if it is worn at the ankle.**

### To sync your device

Plug the dongle into the USB port of your computer.

On your computer, go to: All programs | Fitbit connect | Fitbit connect | Sync now. Follow all subsequent instructions.

After the device has synced, it will ask whether you want to be taken to the website.

### Accessing your information

<http://www.fitbit.com/au> | Log in on top right of screen

Use a study designated email to log in.

The dashboard has a summary of your daily physical activity.

The log is where you may record information about other activities you’ve done during the day, such as swimming or biking.

If you’re interested in tracking your food consumption or other health outcomes, you may record these in **the log** section.

Log out via the settings button on the top right of screen.

### Fitbit One

Battery lasts 10-14 days, participant will need to charge it

Tracks steps, distance, calories burned and stairs climbed

Wear it in pocket, on a belt or bra

Rain, splash, and sweat-proof

### Fit Bit Zip

Does not need participant to charge it, watch battery lasts up to 6 months

Tracks steps, distance and calories burned

Wear it in pocket, on a belt or bra

Rain, splash, and sweat-proof

### Fit Bit Charge

Battery lasts 7-10 days, participant will need to charge it

Tracks steps, distance, calories burned and stairs climbed

Wear it on wrist; functions as a watch and can be connected to iOS or Android phone

Rain, splash, and sweat-proof (not water-proof)

###

### Fit Bit App

Free download from iTunes or Google Play

### Feedback graph

Example of graph to provide feedback to patient about number of weekly steps and work towards goals

**FROM FITBIT LOG ON WEBSITE**


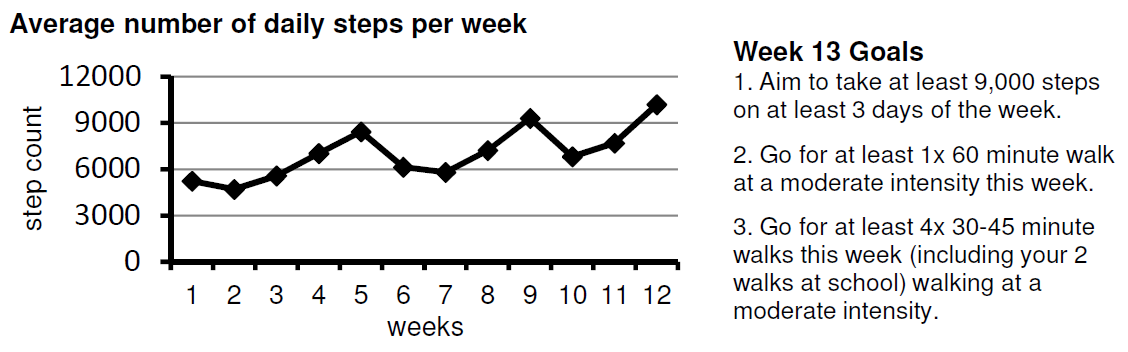
**FROM EXCEL DOCUMENT**

### Example of pedometer log sheet

(*Sheet adapted from: Walk your way to health record sheet; University of Nebraska, Lincoln, USA*)


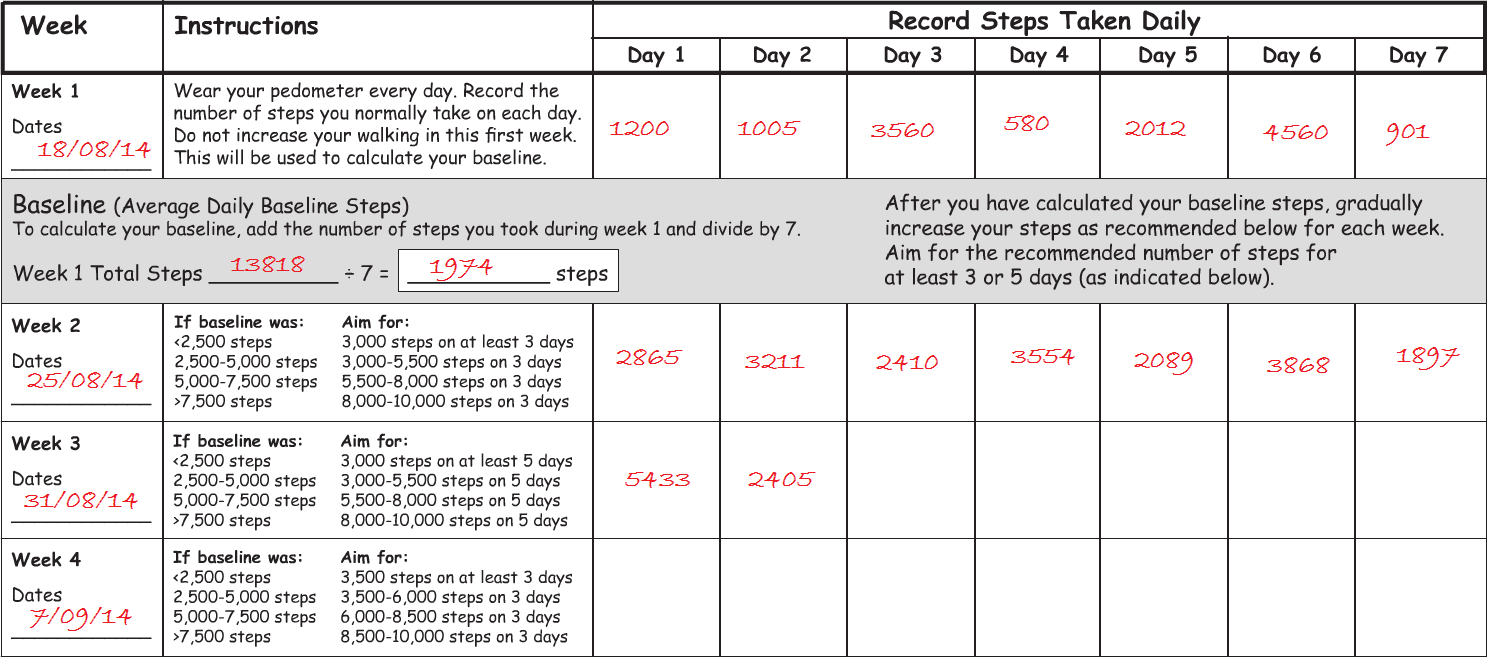


## Garmin Watches (vivofit)

### Installing the Garmin vivofit on your computer or mobile device

1. Go to [www.GarminConnect.com/vivofit](http://www.GarminConnect.com/vivofit).
2. Select an option to either download a computer software or mobile device App.
3. Follow the instruction on your computer or mobile device to pair your Garmin vivofit device and complete the setup process.

If you need some more help, you can download the Owne’r manual (PDF) going to <http://static.garmin.com/pumac/vivofit_OM_EN.pdf>

1. Getting Started
2. Sending data to your Computer or Mobile Device
3. Wearing the Device
4. Icons/Auto goal/Move bar/History/Sleeping tracking
5. Garmin Connect

### Accessing your information

[www.garminconnect.com/start](http://www.garminconnect.com/start) | Sign in on top right of screen

Use your personal email to create an account/log in.

The dashboard has a summary of your daily physical activity.

You can track your progress, store your activities, analyze your data, share your activities and manage your settings.

### Device information

Vivofit specifications:

- Battery life – 1 + year.
- Water rating (5 ATM) – The device withstands pressure equivalent to a depth of 50m.
- Avoid pressing the keys under water.

## PHONE APPS

### Runkeeper

- Download Runkeeper onto the participants’ phone using Google play or iTunes store.
- To use Runkeeper for an outdoor walk:
  - Select “walking” as activity type.
  - Select “start activity”
  - The person starts walking but can pause the activity at any time
  - The App will provide feedback every 5 minutes regarding time and distance walked and average pace.
  - When the participant has finished walking select “stop”
  - The App will move to a summary page which provides a map of the walk, distance walked, duration, average speed, and calories burnt. There is also a notes section that they can add a note if they want e.g. how many times they stopped for a rest, if they walked with someone else, a different walking track
  - Select “save activity”. The App will then give a verbal summary of the results.
  - The research physiotherapist will be able to get the summary results either over the phone with the participant, or during home visit.

###

### Walk Forward

#### Overview of Walk Forward

Walk Forward is an iPhone app that allows a trial group of participants to track their walks, view their past walks, and link up with other trial participants to create a leader board. The app has been developed by The George Institute, and produced by The Project Factory. The purpose of the app is to allow physiotherapists and clinicians to view their participants’ progress, and for participants to be given clear instructions on how much they should be walking each day. The participants will have their walks detailed on maps, each walk they take. They are encouraged to participate with other trial members via a leader board function.

##### Logging In and Administration Overlay

Administrators can log into Walk Forward at the following address:

https://www.walkforward.com.au/login.html?#wf_admin

Trial User Name: **XXXXXXXX** Trial Password: **xxxxxx**

This section allows administration personnel to view the details a participant has entered. All data (each walk and distance by each user) is captured within this portal.

##### Participant information

If you click on the ‘Walk Forward Admin’ tab, you will be taken to a central dashboard.

Administrators can search for participants by user name (in the search window).

All users are listed on the left hand side. There is a summary for each user, which lists their total distance, the number of walks they have completed, the total time they have walked for, and the last time their usage was updated.

##### Viewing Participants Individually

Once you have found the participant you would like to view, you can click on their user name. This brings up a page, which lists their progress with data including walk duration, distance walked, average speed and finish time.

The page is tabbed at the bottom, so administrators can click through each page (displayed at 10 walks per page)

#### App Installation, Versions and Operating Systems

The Walk Forward app can be downloaded from the App store directly onto a participants’ phone. Walk Forward version 1.1 will be developed for use on iPhone 4 devices and upward. It will support OS 7.0 and 8.0. Approximate size of the App is 2.5 MB.

Please note that compatibility with future operating systems will need to be managed on an adhoc basis.

Participants will require an iTunes account in order to be able to download the app directly to their phone.

The Walk Forward app will be published under The Project Factory iTunes account. Users will be required to register in order to use the app and as part of the registration process users will be asked to identify themselves as either trial or non-trial participants. In the first version of Walk Forward, only trial participants will be able to register. They will be required to enter a 4-digit password. This password will be provided to a user by their clinician and will be generic across all trial participants.

**The trial password will be: xxxx**

If a user does not have a trial password code, they will not be able to register.

####

##### User Registration

To register as a user, the participant must enter the following information First Name:

Surname: (Use participant ID)

Email address:

Password:

4-digit Trial Code:

Once they have registered, they can log in to the Walk Forward app at any time by entering their email address and password, and tapping ‘Sign In’.

##### Change Password /Forgot Password Options

If participants forget their password, they can click the ‘forgot password’ button in the sign in screen. They can then enter their email address and click on the link to reset their password.

Once inside the app, participants can change their details, email address or password by going to the ‘Settings’ tab (on far right), and following the prompts to change details as they wish to.

##### Change User Options

If participants want to change users once they’re inside the app, they can go to the ‘Settings’ tab (on far right) and follow the prompts to change user (using the orange button).

##### Participant’s content and admin/physio view

This stage of Walk Forward’s development all data at an administration level can be viewed by everyone. The participant information viewable to an administrator (who has the user name and password to log into the Walk Forward portal) will be the participant’s name (ensure participant is **not** identifiable), number of walks; distance walked, average speed, and last walk undertaken.

#### Using the App

Walk Forward has been designed to be a fun, intuitive and easy to use app for all participants. It aims to capture meaningful data that will help physiotherapists and clinicians improve and enhance their level of participant involvement.

##### Walk Forward Levels (Plans)

There are three ‘walk plan’ levels to choose from. They vary in length of walk, and frequency of walking schedule. Physiotherapists and clinicians will set their recommendation for each participant.

Participants can set their start date when they begin each level. To change this at any time, there is a calendar icon in the top right corner. Participants can click on this icon and enter a different start date for their level. Today’s date is immediately highlighted in yellow in the Plans page, for ease of use.

##### Using App with GPS

Please note that this app only works outdoors. It is enabled with a GPS (global positioning system) which is only activated when the phone is outside. Before commencing a walk, a second counter will count down from 10 to O. A pause button will come up if GPS is not working.

Other than that, it’s very simple. A green arrow will indicate to participants that the GPS signal is working. Participants can walk for the desired length of time. They can place their phone in a pocket or handbag and it will still work. At the end of the walk, participants press the ‘stop’ button. They’re prompted to check if they want to ‘stop’ or ‘pause’, and whether or not they’d like to ‘save’ or ‘delete’ their walk.

Please note that when they initially download the app, participants will be notified by the following message ‘Allow “Walk Forward” to access your location while you use the app? Walk Forward needs to use your location to track your workout’

Participants need to click ‘allow’ in order for the GPS to work and to track the results of their walk. If participants tap ‘don’t allow’ they will receive an explanation on how Walk Forward needs GPS in order to work, and how to change the settings in their phone.

##### History

The History section allows participants to look at their past achievements. They are listed by day and date. Using the far left arrow will allow participants to look at the map of where they walked.

##### Finding Friends

The Friends section allows participants to find others who are also doing the same trial. Using the Head Icon in the top left of the Friends page, participants can find friends via name or email address, then add them to their leader board.

There are three leader boards. They calculate Activities, Distance and Duration respectively.


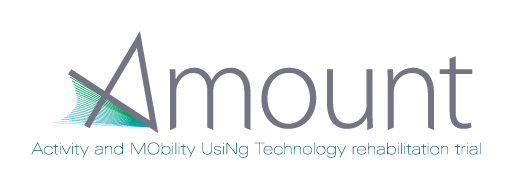


Date: 14/04/14

Assessor ID: __XX________

Participant ID: example ______

# INTERVENTION PLANNING FORM: INPATIENT

Initial physiotherapy assessment reviewed? √

Diagnosis: Traumatic brain injury ______EstimatedLOS:___3months___________

Risk factors (Co-morbidities, non-motor impairments): PTA 57 days, slow processing speed._____________________________________________________________________

Current assistive aides (glasses, hearing/walking aides)_quad stick__________________

Current focus of physiotherapy intervention (motor impairments, mobility tasks being practiced, exercises, therapy goals): __Patient currently practicing functional tasks especially transfers. Main difficulties are standing up from a chair without arm support, reaching in standing, dual tasks_______________________

Current main goals: by end of 6 weeks, 1) stand up from a chair 3 times without arm support in less than 20 seconds; 2) reach 1 object placed forward and on both sides in standing without lifting feet off floor __________________________________

Previous physical activity/hobbies: lots of walking at work (set builder at movie studios) __motor bike riding, snowboarding________________________________

Previous technology use: iPhone_________________________________________

Participant perception of problems and goals: __Left side of body not working properly______________________________________________________________

Initial technology (games/exercises) chosen: _ stepping tiles; Fysiogaming______

Why? ST: will assist to load the leg in sitting, reaching in sitting and sit to stand. FG: sit to stand game engaging, his boys may be interested and help motivate.__

| INPATIENT PRACTICE SHEET |  |  |  |  |  |  |
| --- | --- | --- | --- | --- | --- | --- |
| **Session duration** | **45 min** | **55 min** | **45 min** | **63 min** | **65 min** |  |
| **MOBILITY LIMITATION** | **2** | **2** | **1** | **1** | **1** |  |
| **TECHNOLOGY** | **3** | **3** | **4** | **4** | **4** |  |
| GAME | **Weight shift** | **Weight shift** | **Sit to stand** | **Sit to stand** | **Sit to stand** |  |
| EXERCISE LEVEL | **L2; Boundary 5** | **L2; Boundary 5** | **bed ht:52cm; L1** | **bed ht:50cm; L3** | **Bedht:50cm;L10** |  |
| SETS / REPS / DURATION (min) | **2 \| 10 \| 5** | **2 \| 10 \| 5** | **2 \| 25 \| 6** | **2 \| 24 \| 6** | **2 \| 65 \| 14** |  |
| SCORE / CUES |  |  | **450** | **480** | **1720** |  |
| SET UP \| USE \| RECORD = I/P/A | **A \| P \| A** | **A \| P \| A** | **A \| A \| A** | **A \| P \| A** | **A \| A \| A** |  |
| **MOBILITY LIMITATION** | **2** | **2** | **4** | **4** | **4** |  |
| **TECHNOLOGY** | **2** | **3** | **4** | **4** | **4** |  |
| GAME | **Penguin slide** | **Instability** | **Side walking** | **Side walking** | **Lunges** |  |
| EXERCISE LEVEL | **Beginner** | **Level 2; hold 5s** | **L1** | **L2** | **Level 20** |  |
| SETS / REPS/ DURATION (min) | **3 \| \| 10** | **10 \| \| 12** | **2 \| 20 \| 6** | **2 \| 25 \| 8** | **4 \| 25 \| 10** |  |
| SCORE / CUES | **35,30,40** |  | **400** | **500** | **2860** |  |
| SET UP \| USE \| RECORD = I/P/A | **A \| A \| A** | **A \| P \| A** | **A \| A \| A** | **A \| A \| A** | **A \| A \| A** |  |
| **MOBILITY LIMITATION** |  | **2** | **4** | **4** | **4** |  |
| **TECHNOLOGY** |  | **2** | **4** | **4** | **4** |  |
| GAME |  | **Penguin slide** | **Lunges** | **Lunges** | **Dynamic balance** |  |
| EXERCISE LEVEL |  | **Beginner** | **L20** | **L20** | **L6 random** |  |
| SETS / REPS / DURATION (min) |  | **3 \| \| 10** | **6 \| 15 \| 8** | **4 \| 15 \| 8** | **2 \| \| 8** |  |
| SCORE / CUES |  | **35,30,40** | **2340** | **1890** | **840** |  |
| SET UP \| USE \|RECORD = I/P/A |  | **A \| A \| A** | **A \| P \| A** | **P \| P \| A** | **A \| A \| A** |  |
| **COMMENTS** (e.g. date start teaching; date indep achieved)    **Step count:**  **MOBILITY LIMITATION: [1]** STANDING UP FROM CHAIR, **[2]** MAINTAINING STANDING POSITION, **[3]** REACHING WHILE STANDING, **[4]** STEPPING WHILE STANDING,  **[5]** CHANGING DIRECTIONS WHILE WALKING, **[6]** STAIR CLIMBING, **[7]** PHYSICAL ACTIVITY THROUGHOUT THE DAY  **TECHNOLOGY: [1] XBOX KINECT, [2] NINTENDO WII, [3] HUMAC, [4] FYSIOGAMING, [5] STEPPING TILES, [6] FITBIT, [7] IPAD APP, [8] SMARTPHONE APP**  AMOUNT Intervention Inpatient Practice Sheet Version 5_10/11/2014 | **1^st^ session: Intervention planning sheet; demonstration** | **Set up fitbit + fitbit App on iPAD** | **Review steps on iPAD**  **840** | **Review steps on iPAD**  **658** | **Review steps on iPAD**  **1620** |  |
| **INPATIENT PRACTICE SHEET** | **Monday 27/4** | **Tuesday 28/4** | **Wednesday** | **Thursday** | **Friday** | **Weekend** |
| **Session Duration** |  | **cont.** | **cont.** | **cont.** | **cont.** |  |
| **MOBILITY LIMITATION** |  | **2** | **3** | **3** | **4** |  |
| **TECHNOLOGY** |  | **2** | **1** | **1** | **5** |  |
| GAME |  | **Table tilt** | **Wall breaker** | **Wall breaker** | **Stepping grid** |  |
| EXERCISE LEVEL |  | **Beginner** | **easy** | **easy** | **4 steps** |  |
| SETS / REPS / DURATION (min) |  | **\| 5 \| 11** | **\| 5 \| 10** | **\| 6 \| 12** | **20L\| 20R \|10** |  |
| SCORE / CUES |  | **40** | **543** | **620** | **-** |  |
| SET UP \| USE \| RECORD = I/P/A |  | **P \| P \| A** | **A \| P \| A** | **A \| P \| A** | **A \| P \| A** |  |
| **MOBILITY LIMITATION** |  | **4** | **3** | **3** | **6** |  |
| **TECHNOLOGY** |  | **2** | **1** | **1** | **5** |  |
| GAME |  | **Jogging** | **Fruit Ninja** | **Super saver** | **Step up/ down** |  |
| EXERCISE LEVEL |  | **Beginner** |  | **-** | **10cm block** |  |
| SETS / REPS / DURATION (min) |  | **1 \| \| 5** | **5 \| \| 10** | **\| 5 \| 10** | **50L\| 50R \|12** |  |
| SCORE / CUES |  | **24%** | **120 points** | **-** | **-** |  |
| SET UP \| USE \| RECORD = I/P/A |  | **A \| A \| A** | **A \| P \| A** | **P \| I \| A** | **A \| P \| A** |  |
| **MOBILITY LIMITATION** |  |  |  | **4** | **1** |  |
| **TECHNOLOGY** |  |  |  | **1** | **7** |  |
| GAME |  |  |  | **20000 Leaks** | **Prep for STS** |  |
| EXERCISE LEVEL |  |  |  | **-** | **3/6 ex.’s** |  |
| SETS / REPS / DURATION (min) |  |  |  | **\| 4 \| 10** | **\| \|** |  |
| SCORE / CUES |  |  |  | **90** | **Set-up & demo** |  |
| SET UP \| USE \| RECORD = I/P/A |  |  |  | **A \| P \| A** | **\| \|** |  |
| **COMMENTS** (e.g. date start teaching; date indep achieved  **Step Count:** |  |  |  |  | **To do Prep for STS ex over weekend In room** |  |

**MOBILITY LIMITATION: [1]** STANDING UP FROM CHAIR, **[2]** MAINTAINING STANDING POSITION, **[3]** REACHING WHILE STANDING, **[4]** STEPPING WHILE STANDING,

**[5]** CHANGING DIRECTIONS WHILE WALKING, **[6]** STAIR CLIMBING, **[7]** PHYSICAL ACTIVITY THROUGHOUT THE DAY

**TECHNOLOGY: [1] XBOX KINECT, [2] NINTENDO WII, [3] HUMAC, [4] FYSIOGAMING, [5] STEPPING TILES, [6] FITBIT, [7] IPAD APP, [8] SMARTPHONE APP**

AMOUNT Intervention Inpatient Practice Sheet Version 5_10/11/2014


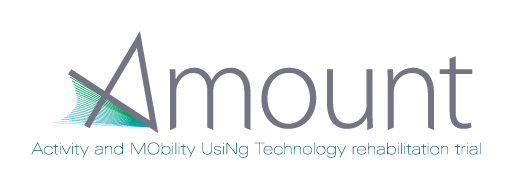


Assessor ID: ______xx________

Participant ID: B0xx ______

# COMMUNITY INTERVENTION COVER SHEET

| **Date contact** | **Scheduled Y/N** | **Format** | **Primary reason for contact** | **Covered during conversation** | **Comments** | **Next review date?** | **Time spent (min)** |
| --- | --- | --- | --- | --- | --- | --- | --- |
| 29/6/15 | Y | 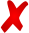🗹 Tel  🞏 email  🞏 VC  🞏 HV  🞏Hosp  🞏SMS | - 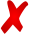Quick contact -nonclinical/tech (e.g. set up app) - Tech support – see tech support sheet :___ - Health coaching –see health coaching sheet no:___   -- see goal review sheet no:___   - Data collection (e.g. SUS, PACES) - Other:____________________________________ | 🞏 Objective data  🞏 Mobility status  🞏 Physical activity status  🞏 Adherence (B & F)  🞏 Tech issues/assistance  🞏 Goal setting/evaluation  🞏 Modifying program  🞏 Falls prevention&education  🞏 PA & health education  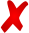🞏 Other: ­­­­­­­­­­__book appt_____ | Called to review technology use + schedule time for H/V | _8_/_7_/_15_ | 5min |
| 8/7/15 | Y | 🞏 Tel  🞏 email  🞏 VC  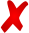🗹 HV  🞏Hosp  🞏SMS | - Quick contact -nonclinical/tech (e.g. set up app)   Tech support – see tech support sheet :___   - 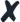Health coaching –see health coaching sheet no:_1_   -- see goal review sheet no:_1_   - Data collection (e.g. SUS, PACES) - Other:____________________________________ | 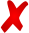🗹 Objective data  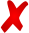🗹 Mobility status  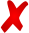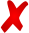🗹 Physical activity status  🗹 Adherence (B & F)  🞏 Tech issues/assistance  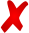🗹 Goal setting/evaluation  🞏 Modifying program  🞏 Falls prevention&education  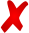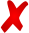🗹 PA & health education  🗹 Other: ­­­­­­­­­­Set up with Xbox__ |  | _15_/_7_/_15 | 60  min |
| 31/7/15 | N | 🞏 Tel  🞏 email  🞏 VC  🞏 HV  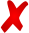🗹Hosp  🞏SMS | - 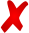Quick contact -nonclinical/tech (e.g. set up app) - Tech support – see tech support sheet :___ - Health coaching –see health coaching sheet no:___   -- see goal review sheet no:___   - Data collection (e.g. SUS, PACES) - Other:____________________________________ | 🞏 Objective data  🞏 Mobility status  🞏 Physical activity status  🞏 Adherence (B & F)  🞏 Tech issues/assistance  🞏 Goal setting/evaluation  🞏 Modifying program  🞏 Falls prevention&education  🞏 PA & health education  🞏 Other: ­­­­­­­­­­________________ | Visit in hospital as admitted for bowel obstruction; F/u on d/c (not using fitbit in hospital) | ___/___/___ | 5min |
| **Date contact** | **Scheduled Y/N** | **Format:** | **Primary reason for contact** | **Covered during conversation** | **Comments** | **Next review date?** | **Time spent (min)** |
|  |  | 🞏 Tel  🞏 email  🞏 VC  🞏 HV  🞏Hosp  🞏SMS | - Quick contact -nonclinical/tech (e.g. set up app) - Tech support – see tech support sheet :___ - Health coaching –see health coaching sheet no:___   -- see goal review sheet no:___   - Data collection (e.g. SUS, PACES) - Other:____________________________________ | 🞏 Objective data  🞏 Mobility status  🞏 Physical activity status  🞏 Adherence (B & F)  🞏 Tech issues/assistance  🞏 Goal setting/evaluation  🞏 Modifying program  🞏 Falls prevention&education  🞏 PA & health education  🞏 Other: ­­­­­­­­­­________________ |  | ___/___/___ |  |
|  |  | 🞏 Tel  🞏 email  🞏 VC  🞏 HV  🞏Hosp  🞏SMS | - Quick contact -nonclinical/tech (e.g. set up app) - Tech support – see tech support sheet :___ - Health coaching –see health coaching sheet no:___   -- see goal review sheet no:___   - Data collection (e.g. SUS, PACES) - Other:____________________________________ | 🞏 Objective data  🞏 Mobility status  🞏 Physical activity status  🞏 Adherence (B & F)  🞏 Tech issues/assistance  🞏 Goal setting/evaluation  🞏 Modifying program  🞏 Falls prevention&education  🞏 PA & health education  🞏 Other: ­­­­­­­­­­________________ |  | ___/___/___ |  |
|  |  | 🞏 Tel  🞏 email  🞏 VC  🞏 HV  🞏Hosp  🞏SMS | - Quick contact -nonclinical/tech (e.g. set up app) - Tech support – see tech support sheet :___ - Health coaching –see health coaching sheet no:___   -- see goal review sheet no:___   - Data collection (e.g. SUS, PACES) - Other:____________________________________ | 🞏 Objective data  🞏 Mobility status  🞏 Physical activity status  🞏 Adherence (B & F)  🞏 Tech issues/assistance  🞏 Goal setting/evaluation  🞏 Modifying program  🞏 Falls prevention&education  🞏 PA & health education  🞏 Other: ­­­­­­­­­­________________ |  | ___/___/___ |  |


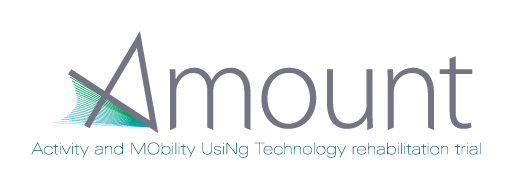


Date: 28/07/15___

Assessor ID: ____xx__________

Participant ID: B0xx ______

# INTERVENTION PLANNING FORM: COMMUNITY

Expected discharge date: _22_ /_07_ /_14_ Actual discharge date: ___/___/___­­

Home environment: __living with wife and 2 boys approximately 8 and 10 years old in a house, 3 steps at front access, no rail___________________________________

Carer involvement: _Carer comes in every morning and evening to help with shower, dressing, and meals ______________________________________________________________________________

Current home technologies (mobile-type, internet, iPad, gaming systems): iPhone ________________

Technology planned for home use: iPad app; fitbit ___________

Date of first home visit: 28/07/14 Actual discharge date: _25/_07/_14

Discharge address: ___smith street, 1 liverpool 2000_________________________________

Phone: __1234567890_________________ Mobile: ___1234567890____________________

NOK: ___Lucy___________________ Relationship to Participant: __wife___________________

NOK phone: _1234567890____________ NOK mobile: ___1234567890________________

GP:__ Dr Paul Robson__________________ GP Phone:___ 1234567890_________________

Other rehabilitation/services referred on discharge (e.g. PT,OT, homecare): _PT, OT, social worker___

Technologies chosen for community program: _ iPad app; fitbit_______________________________

Preferred format for review sessions: phone call email videoconference

Technologies set up in home (date, issues): iPad app on 27/07/14, available table to place iPad is the same as dinner table, wife will need to help set up every time and is concerned because she works full time and leaves right after carer comes in the morning__________________

Community program goals established: √

Equipment loan agreement completed: √

Intervention folder updated and given to participant: √

First review due: 11/08/14 Phone call Email Videoconference Home visit

SMS reminder for review session? Yes/No day__10/08/14 time_10am______


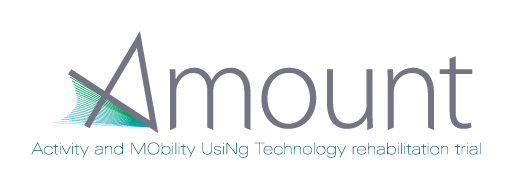


Date: 28/07/14___

Assessor ID: ____xx__________

Participant ID: B0xx ______

# INITIAL HEALTH COACHING SESSION

Review number: ______01_________ Duration of session (mins):____60________

**Format:**  Phone call Email (please attach) Videoconference Home visit Hosp visit

**Subjective report** (How’s it going?):

Pt is progressing well and enjoying using iPad app for exercises. Pt reports he is complying with exercises 5x/wk and is finding them increasingly easy and not challenging, losing motivation.

Now able to stand up from a chair without using the arms for support, changed quad stick to 1 single point stick. Has forgotten to wear fitbit, left on old shoes.

**Objective data** (if applicable e.g. pedometer step count, exercises completed from iPad program, distance walked data from phone APP):

IPad app reps per day: 50x2 stand up from high chair with both feet on floor, 50x2 stand ups from high chair with one foot stool, 100 reaches for object in sitting, 80 stand ups.

**Directed discussion** (e.g.mobility status, physical activity status [active transport, leisure, household, work], adherence to prescribed program [barriers & facilitators], technology use/issues, goal progression)

Pt educated towards ability to change number of reps by himself if not challenging enough, also pt can communicate with us via email or phone number if would like new exercises, email and ph number reviewed and noted down for pt.

New goal: stand up dining room height chair, pick up small object from table, walk a few steps and return to sit down.

**Feedback and advice given** (e.g. modifying program, falls prevention and education, PA & health education, addressing barriers and facilitators, goal review)

Walk close to the table or wall for safety, decrease height of chair for stand ups.

At end of call, go and put fitbit on current shoes.

Any other comments: plan introduce Xbox games to increase motivation and interest

**Next review**: Day _Tuesday_______ Date_30/08/14 Time__10am_________

Format: Phone call Email Videoconference Home visit

SMS reminder for review session? Yes/No Day: ___29/08/14__ Time: __10am__

# INTERVENTION TECHNOLOGY SUPPORT

Date: 16/09/14

Assessor ID: __ XX________

Participant ID: B0XX ______

Review number: ___02_______ Duration of session (mins):_____ 10______

**Format:**

Phone call Email (please attach) Videoconference Home visit Hospital visit SMS

**Type of technology:**

Pedometer ______Fitbit one__________ iPad-Amount app iPad – T-Rex app

Xbox Wii PA App______________ Other: Fitbit app – not updating

data from one

**Technical issue:**

Fitbit app – not synchronizing with fitbit one. Rerported that iPad saying “ blue tooth not working” despite Bluetooth being one in setting on iPad.

**Advice/support given:**

Attempted to troubleshoot over the phone. Checked Bluetooth one in settings internet.

Internet working. Fitbit working.

**Any follow up required?**

Yes – arranged for 2/7 pt to count to log daily steps count on pedometer recording sheet.

**Next review**: Day Monday Date: 19/09/14 Time____1pm______

Format: Phone call Email Videoconference Home visit Hospital visit

SMS reminder for review session? Yes / No Day _____________ Time_____________

| COMMUNITY GOAL SETTING goal sheet number_1___ | | | | |
| --- | --- | --- | --- | --- |
| **Date Goal Set** | **Goal statement:** | **Progress/Comments** | **Review Date** | **Rating** |
| 18/01/16 | **Global goal:** I will walk the kids to school at least 3 times each week in 3 months’ time | 18/02: Has walked to school once so far with husband with her, report fatigue ++. |  |  |
| 18/01/16 | **Sub-goal:** I will go for a 10 minute walk 3 times this week using the Walk Forward App on my phone. | Achieved, viewed walk on walk forward app. | 25/01/16 | 4 |
| 18/01/16 | **Sub-goal:** I will complete my iPad exercises 5 times each week for 4 weeks. | Exercises 3x, 4x, 3x, 4x per week over the 4 weeks- added alarm into iPad to remind to do exercises | 18/02/16 | 3 |
| 25/01/16 | **Sub-goal:** I will take at least 2500 steps on at least 3 days this week as measured using my Fitbit. | >2500 steps on 5 days. | 02/02/16 | 5 |
|  | **Sub-goal:** |  |  |  |
|  | **Sub-goal:** |  |  |  |

**Goal Ratings: 1=Not achieved 2= Partially achieved (1-49%) 3=Mostly achieved (50-94%) 4=Achieved (95-105%) 5=Achieved+ (>105%)**

| **COMMUNITY GOAL SETTING** goal sheet number____ | | | | |
| --- | --- | --- | --- | --- |
| **Date Goal Set** | **Goal statement:** | **Progress/Comments** | **Review Date** | **Rating** |
|  | **Single goal:** |  |  |  |
|  | **Single goal:** |  |  |  |
|  | **Single goal:** |  |  |  |
|  | **Single goal:** |  |  |  |
|  | **Single goal:** |  |  |  |
|  | **Single goal:** |  |  |  |

**Goal Ratings: 1=Not achieved 2=Partially achieved(1-49%) 3=Mostly achieved(50-94%) 4=Achieved(95-105%) 5=Achieved+(>105%)**
