# Supplementary material for: Digitally enabled aged care and neurological rehabilitation to enhance outcomes with Activity and MObility UsiNg Technology (AMOUNT) in Australia: A randomised controlled trial
Source: PLoS Med. 2020 Feb 18;17(2):e1003029. doi: 10.1371/journal.pmed.1003029 (PMC7028259; doi:10.1371/journal.pmed.1003029)
Supplement: S2 Text — (DOC) [file pmed.1003029.s010.doc]

**AMOUNT rehabilitation trial: study protocol and intervention protocol amendments**

**Study protocol versions**

**Protocol versions:**

| Protocol version number | Date approved by Southern Adelaide Clinical Human Research Ethics Committee (lead committee) | Page number |
| --- | --- | --- |
| 1 | 19/12/2013 | 3 |
| 2 | 10/02/2014 | 22 |
| 3 | 29/05/2014 | 41 |
| 4 | 18/08/2014 & 25/11/2014 | 62 |
| **TRIAL RECRUITMENT COMMENCED SEPTEMBER 2014** | | |
| 5 | 14/11/2014 | 83 |
| 6 | 30/06/2015 | 105 |
| Published final protocol | June 2016 | 127 |

**Differences between protocols:**

Differences between protocols 1 & 2:

- Development of Master Participant Information Sheet (PIS) to be used at all three recruitment sites
- Inclusion of an additional period of collecting activity levels for 7 days using the ActivPal (at baseline)
- Provision of a shortened title for the trial.

Differences between protocols 2 & 3:

- Change to inclusion criteria to include participants with cognitive impairment. Those with a score of >21 on the MMSE will be approached directly, and those with a MMSE score of <21 whom the treating clinician feels could benefit from this intervention will have the next of kin advised of the study and provided a PIS. A new proxy PIS and consent form has been developed for this purpose.
- Change to inclusion criteria to have a likely life expectancy greater than 6 months from 12 months
- Change to inclusion criteria to have the clinician assess capacity for improvement in mobility from the clinician considering the intervention to be appropriate for the individual.
- Change to exclusion criteria to remove the ability to balance safely in standing as the intervention provided will be one-to-one.
- Dose of intervention was changed from ‘at least 60 minutes per day’ to a more feasible ‘at least 30-60 minutes per day’ to improve adherence.
- More comprehensive list of equipment to be used in the intervention. The exercise protocol guide is to be reviewed quarterly in order to add new technologies throughout the trial.
- Some outcome measures were changed due to further investigation, discussion and trialling. Additional measures were: De Morton Mobility Index, Single leg stance, maximal balance range test, WHO Disability Assessment Scale 2.0, Telephone-Interview for Cognitive Status, Activities Specific Balance Confidence Scale, Incidental & Planned Exercise Questionnaire, Modified Computer Self-Efficacy Scale and the Functional Comorbidity Index.

Differences between protocols 3 & 4:

- 18/08/2014: Addition of a new measure of cognition, the Trail Making Test, which is a continuous measure and has the potential to show between group differences.
- 25/11/2014: Amendment of proxy PIS and consent form for Sydney sites as per instructions of NSW Guardianship Tribunal to change wording from ‘families/next of kin’ to ‘person responsible’ and to add a paragraph explaining what it means to be the personal responsible in NSW.

Differences between protocols 4 & 5:

- Addition of honours sub-study: How hard do people work in therapy involving technology versus traditional therapy in rehabilitation?

Differences between protocols 5 & 6:

- Addition of mixed methods sub-study: Therapist preferences and experiences of implementing affordable technologies to improve physical activity levels and mobility outcomes in rehabilitation.

Differences between protocol 6 and published protocol:

- Assessment:
  - TICS measure of cognition which was planned for use in the study was removed prior to the study starting as the Trails A & B was a measure of cognition.
  - Baseline activPAL data was collected for 24 hours instead of 7 days to enable it to be measured prior to randomisation. This was implemented prior to the study starting.
- Intervention:
  - Physiotherapy support of participants in the community was initially recommended to be weekly, but was changed to weekly-fortnightly during the first few months of the trial to enable more tailored and flexible approach as supported by staff and participants.

**Intervention protocol versions**

1. Original intervention protocol, dated 14^th^ August 2014.
2. Intervention protocol V2, dated 14^th^ October 2015.
3. Intervention protocol V3, 23^rd^ February 2016.

**Differences between protocols:**

Differences between protocols 1 & 2:

- Additional information was added to version 2 to further describe the post-hospital setting and the provider of the intervention. The frequency of the post-hospital support was initially prescribed weekly but changed within the first 6 months of the trial to “as required” with a recommendation of weekly initially, reducing the frequency over time if the participant was managing well. This was modified due to experience in the trial and matched the tailored intervention. This modification was reflected in version 2 (red writing is additional information in version 2).

## Setting

- **Version 1:** Inpatient for initial intervention, progressing to home-based once discharged. The inpatient intervention will be conducted within the physiotherapy rehabilitation gyms of the site hospitals.
- **Version 2:** The inpatient intervention will be conducted within the physiotherapy rehabilitation gyms of the site hospitals. The community-based program will commence once discharged from a rehabilitation ward and can include home, transitional living ward or residential care settings.

## Provider of intervention

- **Version 1:** The research physiotherapist will determine the most appropriate intervention based on discussion with the clinical physiotherapist, baseline assessment, participant goals, and the technology suitable in accordance with the intervention protocol. The research physiotherapist will then provide the inpatient intervention one-on-one for the length of their inpatient rehabilitation, and then using phone email or Skype contact weekly once they are home (a home visit may be required to set up the technology prior to discharge, and up to 5 home visits may be provided during the trial if there are problems with the technologies or if technologies are upgraded).
- **Version 2:** The research physiotherapist will determine the most appropriate intervention based on discussion with the clinical physiotherapist, baseline assessment, participant goals, and the technology suitable in accordance with the intervention protocol. The research physiotherapist will provide the inpatient intervention one-on-one for the length of their inpatient rehabilitation, and then support the community program by using phone, email, or video conference contact once they are home. A home visit may be required to set up the technology prior to discharge, and up to 5 home visits may be provided during the program if there are problems with the technologies or if technologies/program needs upgrading.

## Frequency

- **Version 1:** ≥5 days per week
- **Version 2:** Participants should participate in technology-based intervention ≥5 days per week for the length of the program. The research physiotherapist will provide one-on-one intervention 5x per week during the inpatient stage. The research physiotherapist will provide support and health coaching as required by each participant during the community stage. It is recommended support is provided weekly to begin with, but the frequency can reduce if the participant is managing their program well.
- No information from version 1 was taken away when the protocol was updated. The following information was added to version 2.
  - Getting started with Wii Family Trainer and Wii Family Trainer games
  - Nintento WiiU instructions for set up and use and Nintendo WiiU games.
  - Information about Fitbit Charge model added to protocol.
  - Information about Garmin Vivofit model added to protocol.
  - Information about Investigator developed Walk Forward phone App
  - Inpatient practice sheet updated
  - Community intervention cover sheet
  - Initial health coaching session
  - Intervention technology support sheet
  - Community goal setting sheet updated

Differences between protocols 2 & 3:

- Software update versions for Humac, Fysiogaming and AMOUNT App
- Additional games added to Fysiogaming table
- Instruction on wearing Fitbit updated for slower walkers.
