# Supplementary material for: Digitally enabled aged care and neurological rehabilitation to enhance outcomes with Activity and MObility UsiNg Technology (AMOUNT) in Australia: A randomised controlled trial
Source: PLoS Med. 2020 Feb 18;17(2):e1003029. doi: 10.1371/journal.pmed.1003029 (PMC7028259; doi:10.1371/journal.pmed.1003029)

**Statistical Analysis Plan**

**Effect of affordable technology on physical activity levels and mobility outcomes in Rehabilitation: the Activity and MObility UsiNg Technology (AMOUNT) Rehabilitation trial.**

**Final version, all outcomes**

| **CIs** | **Research Managers** |
| --- | --- |
| Catherine Sherrington | Leanne Hassett |
| Richard Lindley | Maayken van den Berg |
| Maria Crotty |  |
| Annie McClusky |  |
| Hidde van der Ploeg |  |
| Stuart Smith |  |
| Karl Schurr  **Statistician**  Stephane Heritier |  |

Signed


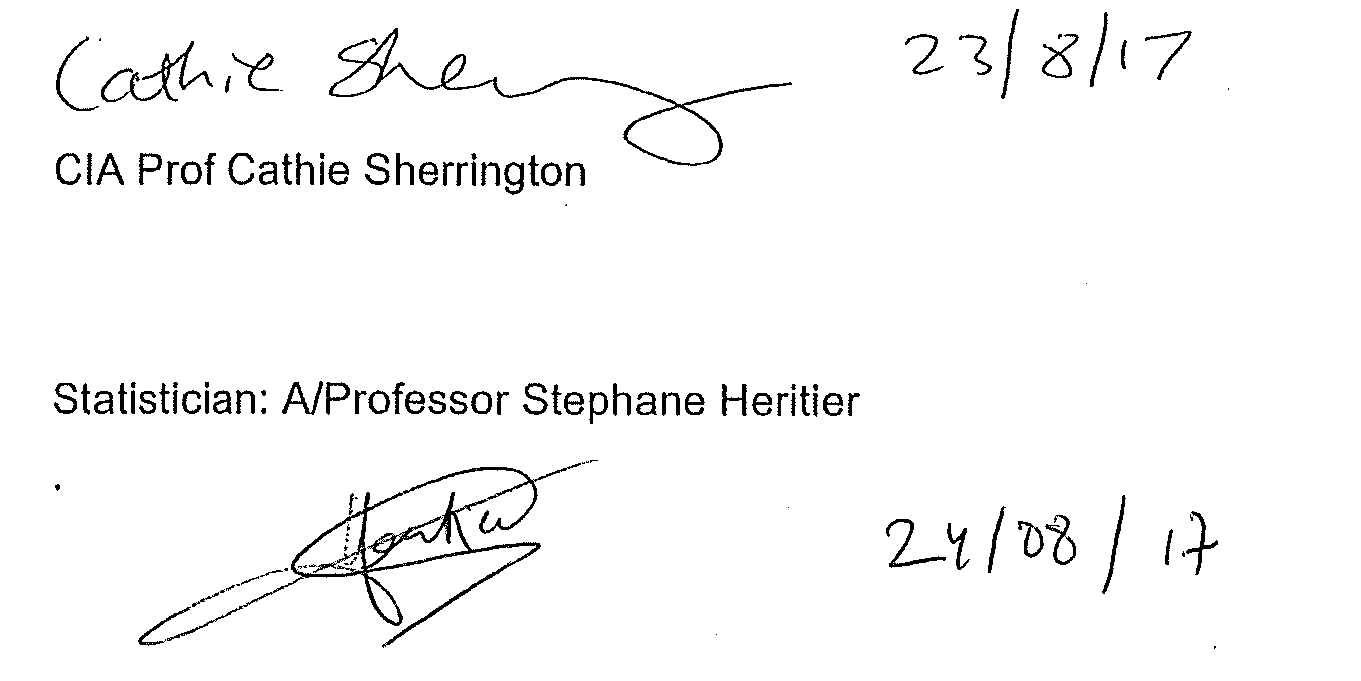


Contents

[1. INTRODUCTION](#_Toc477267551) 2

[2. DATASET ANALYSED](#_Toc477267552) 3

[3. STUDY OUTCOMES 3](#_Toc477267553)

[3.1 Primary outcomes 3](#_Toc477267554)

[3.2 Secondary outcomes 3](#_Toc477267555)

[3.3 Additional measures in the intervention group 6](#_Toc477267557)

[4. STATISTICAL ANALYSIS 6](#_Toc477267558)

[4.1 Analysis principles](#_Toc477267559) 6

[4.2 Data quality and blind review](#_Toc477267560) 7

[4.3 Activity (primary outcome) 7](#_Toc477267561)

[4.4 Mobility (primary outcome) 7](#_Toc477267563)

[4.5 Secondary outcomes 7](#_Toc477267564)

[4.6 Sub-group analyses 8](#_Toc477267565)

[4.7 Economic evaluation 9](#_Toc477267566)

[4.8 Exploratory analyses 9](#_Toc477267567)

[4.8 Intervention group measures 9](#_Toc477267568)

[Appendix 1: Primary and Secondary Outcome Measures 11](#_Toc477267569)

[Appendix 2: ActivPAL data cleaning 1](#_Toc477267569)5

# **1. INTRODUCTION**

People with mobility limitation can benefit from rehabilitation programs, particularly rehabilitation programs that contain intensive repetitive exercises. New technologies potentially provide an affordable way to increase the dose of exercise and overall physical activity for people in rehabilitation. Exercise-based video and computer games/exercises and activity monitors are increasingly being used in rehabilitation settings to provide feedback on motor performance and physical activity. A randomised feasibility study conducted by our team has demonstrated that a tailored approach to delivering task specific repetitive exercise using a suite of technologies appears promising. However, it requires rigorous evaluation with an adequately powered randomised controlled trial. Therefore, this trial aims to evaluate the effect of the addition of affordable technology to usual care on physical activity and mobility.

The primary research question is:

What is the effect of the addition of affordable technology to usual care on physical activity and mobility 6 months after randomisation in people with mobility limitations admitted to inpatient aged and neurological rehabilitation units compared to usual care alone?

Secondary research questions are:

1. What is the effect of the addition of affordable technology to usual care on physical activity levels and mobility 3 weeks after randomisation in people with mobility limitations admitted to inpatient aged and neurological rehabilitation units compared to usual care alone?

2. What is the effect of the addition of affordable technology to usual care on cognition (3 weeks and 6 months after randomisation), self-reported physical activity, activity performance and participation, utility based quality of life, balance confidence, technology self-efficacy (3 and 12 weeks and 6 months after randomisation) and falls (over the 6-month period after randomisation) in people with mobility limitations admitted to inpatient aged and neurological rehabilitation units compared to usual care alone?

3. How do participants randomised to the intervention group report the usability and enjoyment of technologies prescribed in this trial at 3 and 12 weeks and 6 months after randomisation?

Other research questions (not covered as part of the SAP) are:

1. What is the cost-effectiveness of the intervention compared to usual care over the 6-month period after randomisation?

2. What factors predict greater use of technology for participants randomised to the intervention group over the 6-month period after randomisation?

3. What are the experiences of participants and staff using affordable technology for physical rehabilitation?

The study was approved by the Southern Adelaide Clinical Human Research Ethics Committee, Adelaide, Australia (study number 529.13) and the South Western Sydney Local Health District Human Research Ethics Committee, Sydney, Australia (reference number HREC/13/SAC/359). The findings will be disseminated in peer-reviewed journals and through professional and scientific conferences.

The trial was registered with the Australia New Zealand Clinical Trials Register (ACTRN12614000936628).

The protocol paper has been published:

Hassett L, van den Berg M, Lindley R.I, Crotty M, McCluskey M, van der Ploeg H.P, Smith S.T, Schurr K, Killington M, Bongers B, Howard K, Heritier S, Togher L, Hackett M, Treacy D, Dorsch S, Wong S, Scrivener K, Chagpar S, Weber H, Pearson R, Sherrington C (2016). Effect of affordable technology on physical activity levels and mobility outcomes in rehabilitation: protocol for the AMOUNT (Activity and MObility UsiNg Technology) rehabilitation trial. *BMJ Open* 2016; 6:e012074. doi:10.1136/bmjopen-2016-012074.

# **2. DATASET ANALYSED**

The main (intention-to-treat (ITT)) dataset analysed will be constituted of all patients randomised into the study irrespective of adherence to interventions.

#

# **3. STUDY OUTCOMES**

## 3.1 Primary outcomes

The coprimary outcomes will be (1) physical activity (proportion of the day spent upright) and (2) mobility (lower extremity continuous summary performance measure derived from the Short Physical Performance Battery, SPPB) 6 months after randomisation.

1. Physical activity as the proportion of the day spent upright, assessed over a 7-day period using the activPAL activity monitor (PAL Technologies Ltd, Glasgow, UK) at 6 months post randomisation will be the primary outcome. Upright time is calculated as the sum of the time spent stepping and standing. Wear-time authentication was performed on each participant’s data set to determine whether data were to be included in the analysis. Acceptable wear time was set a priori and defined as 4 days, for 24 hours per day. See Appendix 2 for a full description of the validation and data preparation process.
2. Mobility will be lower extremity continuous summary performance score (CSPS)^1^(0–3) of the SPPB.^2^ The SPPB involves measurement of timed ability to stand unsupported (for 10 s) with the feet in three different positions of increasing difficulty (feet together, semitandem and tandem), time taken to walk 4 m and time taken to stand up from a chair and return to a seated position five times. The 6-month CSPS will be the primary outcome measure.

Using the formula to calculate the CSPS^1^, and based on our own baseline data: a score of 0 will be given for participants unable to perform the task for the timed standing balance components (as a low score is a poor score); 11.2 cm/sec for gait (a value corresponding to the 99^th^ percentile [i.e. worst 1% who could perform the task] of baseline performance of individuals completing the task) was assigned to individuals who were physically unable to perform the task or who had a performance below the 99^th^ percentile; 83.0 s (a value corresponding to the 99th percentile of baseline performance of individuals completing the task) was assigned to individuals who were unable to perform the five chair stand or who had a performance above the 99^th^ [i.e. worst 1% who could perform the task] percentile.

In summary, the two primary endpoints will be summarised by:

1. The between-group difference in the proportion of the day spent upright (assessed using the activPAL) at 6 months.
2. The between-group difference in mobility (assessed using the CSPS) at 6 months.

## 3.2 Secondary outcomes

Secondary outcomes will be measures of physical activity and mobility, cognition, self-reported physical activity, activity performance and participation, utility-based quality of life, balance confidence, technology self-efficacy and falls in the 6 months after randomisation. In addition, self-reported measures of technology usability and enjoyment will be obtained from the intervention group.

The endpoints are between-group differences in the following:

**Balance and mobility:**

1. proportion of the day spent upright at 3 weeks (time spent standing and stepping from activPAL)
2. average number of steps per day at 3 weeks and 6 months from activPAL
3. average number of sit to stands per day at 3 weeks and 6 months from activPAL
4. SPPB, at 3 weeks, CSPS (0-3)
5. SPPB, at 3 weeks and 6 months, total score (0-12). The SPPB total score is calculated from three components: the ability to stand for up to 10 seconds with feet positioned in three ways (together side by side, semi-tandem and tandem), time to complete a 3 or 4-metre walk and time to rise from a chair five times.^2^ For the tests of standing balance a score is given depending on the ability to maintain balance in each of these positions. For the other two tests, scores are given firstly on the ability to complete the tasks and secondly the time taken to complete each task. Each task is scored out of 4, with the scores from the 3 tests summed to give a total, with a maximum of 12 and a minimum of 0. A higher score indicates a higher level of function, while lower scores indicate a lower level of function.
6. SPPB, at 3 weeks and 6 months, subscale scores for balance tests, gait speed test and chair stand test (0-4).
7. de Morton Mobility Index (DEMMI) score^3^, at 3 weeks and 6 months. The DEMMI is a 15-item hierarchical measure that covers the mobility spectrum from bed mobility to high-level independent mobility. The DEMMI gives a raw score out of 19 and this is converted into a DEMMI score between 0 to 100 (see appendix 3). A higher score indicates a higher level of mobility.
8. Single leg stance score, at 3 weeks and 6 months. Timed single leg stance (max 10 s).
9. Maximal balance range test at 3 weeks and 6 months, measures the maximum distance participants can lean backward and forward.^4^ The maximum anterior-posterior displacement in mm is the variable used.
10. Step Test at 3 weeks and 6 months, the number of times the participant is able to step one foot on, then off, a 7.5cm block as quickly as possible in 15 seconds.^5^ The average number of steps between the two legs is the variable calculated.

**Activity and participation**, the WHO Disability Assessment Schedule 2.0 (WHODAS 2.0)^6^, a 12-item self-report measure of activity performance and participation covering 6 domains, will be completed at 3 weeks, 12 weeks and 6 months. The scores assigned to each of the 12 items – “none” (1), “mild” (2) “moderate” (3), “severe” (4) and “extreme” (5) – are summed to give a score ranging from 12 to 60, where 12 = no disability and 60 = full disability.

**Physical activity**, the 10-item Incidental and Planned Exercise Questionnaire^7^ assesses the level of physical activity relating to both basic and more demanding activities at 3 weeks, 12 weeks and 6 months. The total time spent doing activities is summed across all components and expressed as hours per week. The score is derived from multiplying frequency score and duration score (see Appendix 4) to create a total duration for the week score: Total activity score = (Q1*Q2) + (Q3*Q4) + (Q5*Q6) + (Q7*Q8) + (Q9*7) + (Q10*7), with a higher score indicating a higher physical activity level. Subscale scores will also be calculated for home exercise (Q2) and walking activity (Q5*Q6)+(Q6*Q7).

**Fall-related confidence** will be assessed with the Activities-Specific Balance Confidence Scale^8^ at 3 weeks, 12weeks and 6 months. This is a 16-item scale with each item scored between 0 to 100 with 0 representing no confidence and 100 representing complete confidence. The 16 items are summed together and then divided by 16 (the number of items) to give a total score between 0 and 100 with a higher score indicating a higher confidence in balance.

**Cognition**, will be assessed using the Trail Making Test parts A and B^9^ at 3 weeks and 6 months. Trails Part A measures processing speed and involves participants connecting consecutive numbers (e.g., 1-2-3). Part B is a measure of executive function of ‘task shifting’ and involves participants connecting alternating letters and numbers (e.g.,1-A-2-B). The difference in time between the two parts (B minus A) will be calculated to isolate the executive component of this test with a lower time reflecting better executive function.^10^ Trails Part A score and Trails Part B scores will also be calculated.

**Health-related quality of life:**

1. European Quality of Life-5 dimensions (EQ-5D-5L)^11^ assessed at 3 weeks 12 weeks and 6 months. The EQ-5D-5L has 5 questions each with 5 levels as well as a VAS score from 0 to 100. A health utility score will be determined from the 5 questions using published scoring algorithms. In addition, the score for each item will be determined and the EQ VAS score (0-100) will be reported.
2. Short Form 6-dimensions questionnaire (SF-6D) calculated from the SF-36^12^ assessed at 3 weeks, 12 weeks and 6 months. The 11 questions are in 6 domains (physical functioning, role participation, social functioning, bodily pain, mental health, and vitality) and using the published Australian scoring algorithm, we will determine the resulting SF-6D utility score.

**Falls** will be assessed over the 6-month period after randomisation. Data will be collected during the inpatient stay using study site incident documentation systems and hospital databases. After discharge, participants will be asked to complete monthly calendars for the remainder of the trial documenting any falls and service usage. Data will be obtained by phone from participants who are unable or unwilling to complete a calendar.

**Technology self-efficacy,** will be measured using the Modified Computer Self-Efficacy Scale^13^ assessed at 3 weeks, 12 weeks and 6 months. This is a 10-item scale, each item scored between 1 (not at all confident) and 10 (completely confident). The total score is the sum of each item score and will be between 10 and 100 (higher score associated with higher levels of confidence). Exposure to common technologies (e.g., smartphone, computer) in the month prior to hospitalisation and during their inpatient and community components of the trial will be measured using a purpose-designed survey.

**Adverse events** (minor and serious) will be monitored throughout the trial. For the purpose of this trial an adverse event is defined as an unwanted and usually harmful outcome (e.g., fall, seizure, cardiac event). The event may or may not be related to the intervention, but it occurs while the person is participating in the intervention, that is, while they are undertaking mobility or physical activities using technology. Adverse events will be categorised as minor AEs (MAEs) or serious AEs (SAEs). A MAE is defined as an incident that occurs while the person is participating in the intervention that results in no injury or minor injury. For example, a fall where the person sustains a small cut or bruise that requires none or minor medical intervention. A SAE is defined as an incident that occurs while the person is participating in the intervention that results in death, serious injury or re-hospitalisation. Examples of SAEs are death, myocardial infarction, serious falls, serious fractures, epilepsy/seizures.

**Deaths** in both groups will be documented.

## 3.3 Additional measures in the intervention group

Adherence to the intervention will be calculated using records kept by staff, usage diaries kept by participants and data available from the devices themselves. In addition, the treating physiotherapist will estimate a global level of adherence (in five categories: 0%, <25%, 25–49%, 50–74% and ≥75%) during the 6-month intervention.

**Usability,** the impression of usability of different technologies will be measured using the System Usability Scale (SUS)^14,15^ assessed at 3 weeks, 12 weeks and 6 months. The SUS is a 10-item scale in which each item is scored between 1 (strongly disagree) and 5 (strongly agree). Items were a mix of positive and negative statements. To adjust for this, items 1,3,5,7 and 9 score contribution is the scale score minus 1 and for items 2,4,6,8 and 10 the score contribution is 5 minus the scale score. The total score is the sum of the adjusted item scores multiplied by 2.5 and ranges from 0 to 100, with a higher score indicating greater usability.

**Enjoyment,** the impression of enjoyment using different technologies will be assessed with the Physical Activity Enjoyment Scale (PACES)^16^ assessed at 3 weeks, 12 weeks and 6 months. The PACES is an 18-item scale in which each item is scored between 1 and 7. Item scores are reversed for questions that give a lower score for a positive comment e.g. 1=I enjoy it and 7=I hate it. The following questions have their scores reversed: questions 1,4, 5,7,9,10,11,13,14,16,17. The total score is the sum of the 18 items and ranges from 18 to 126, with a higher score indicating greater enjoyment.

# **4. STATISTICAL ANALYSIS**

Between-group comparisons for each of the continuously scored outcome measures will be made using linear models with baseline scores entered as a covariate. Fall rates between groups will be compared using negative binomial regression. Interaction terms will be used in the models to assess whether effects of the intervention differed according to age or a neurological versus non-neurological cause of the mobility limitation. Primary analyses will be pre-planned, conducted while masked to group allocation and use an intention-to-treat approach. All analyses will be overseen by the study statistician (SH).

## 4.1 Analysis principles

• Data will be coded to permit blinding to group allocation in the analysis of primary outcomes.

• The primary analyses will be conducted in accordance with the intention-to-treat principle.

• All tests are two-sided and the nominal level of α will be 5%.

• All statistical analyses will be adjusted for baseline scores for that variable (except WHODAS and IPEQ for which baseline scores were not measured) but no other variables except where indicated. Sensitivity analysis will be conducted not adjusting for baseline scores for the coprimary outcomes only.

• Subgroup analyses will be pre-specified and outlined below. These will be carried out irrespective of whether there is a significant treatment effect on the primary outcome.

• Where data are missing due to loss to follow up, we will report the number of observations; we will not impute missing values for the primary analyses. Sensitivity analysis may be conducted for the primary outcomes using multiple imputation of missing data if more than 10% of outcome data are missing.

• P-values will not be adjusted for multiplicity. A significant effect must be observed on both co-primary endpoints to declare the intervention effective. However, the outcomes and time points are clearly categorised by degree of importance (primary and secondary) and a limited number of subgroup analyses will be pre-specified.

• Descriptive statistics will include numbers and percentages of participants with specific scores or means and standard deviations in each group at baseline and follow-up.

##

## 4.2 Data quality and blind review

Data quality checks will initially be conducted in REDCap. Range checks will be performed for each variable at each time point. The following tasks will be conducted blinded to group allocation: 1) Missing and out of range data in REDCap will be checked against paper data collection forms to confirm values and corrected in REDCap; 2) activPAL data will be cleaned and corrected in REDCap as outlined in appendix 2; 3) replacement of missing data for participants unable to perform the test task as outlined in appendix 1.

Once this process is complete and continuing using a blinded review range checks will be performed for each variable at each time point and histograms will be visually inspected for each variable at each time point using stata. The distribution of continuous variables will be evaluated to inform whether change scores will be used for analysis.

## 4.3 Activity (primary outcome)

## The time spent upright (minutes per day averaged over days worn), assessed using the activPAL activity monitor will be treated as a continuous variable. The effect of group allocation on the outcomes at 6-month follow-up will be analysed using linear regression models with baseline scores entered into the linear regression models as covariates. Or if distributions are overly skewed (on visual inspection of histograms) the change scores (post-pre) will be analysed.

## 4.4 Mobility (primary outcome)

The primary analyses will be conducted using linear models with baseline scores entered as a covariate. The groups will be compared on 6-month assessment scores. The primary analysis will use the CSPS^1^ This is the continuously scored version of the Short Physical Performance Battery^2^ and gives a composite score between 0 and 3 based on timed performance of three mobility tasks: the ability to stand for up to 10 sec with feet in different positions (together, side by side, semi-tandem and tandem), 4-metre walk and time to rise from a chair five times.

The primary analysis will be adjusted only for baseline scores. A secondary adjusted analysis will be conducted adjusting for the stratification factors of study site and health condition (whether or not the person has a neurological condition that affects daily activities). Further adjusted analyses will be conducted if major imbalances between the groups at baseline are observed.

If outliers are present in the data or the model assumptions are grossly violated after treatment is included in the model, some sensitivity analyses based on robust regression will be conducted.

## 4.5 Secondary outcomes

Physical Activity:

- The effect of group allocation on the continuous variable time spent upright (minutes per day averaged over days worn), assessed using the activPAL activity monitor at 3 week follow-up will be analysed using linear regression models with baseline scores entered into the linear regression models as covariates.
- The effect of group allocation on the continuous variable ‘average number of steps per day’ assessed using the activPAL activity monitor at 3 weeks and 6 months follow-up will be analysed using linear regression models with baseline scores entered into the linear regression models as covariates.
- average number of transitions per day’ at 3 weeks and 6 months follow-up will be analysed using linear regression models with baseline scores entered into the linear regression models as covariates.

Mobility:

- For this continuous secondary outcome, the groups will be compared on 3 week assessment CSPS, adjusted for baseline scores.
- A secondary analyses will be conducted using the 12-point scored version of the Short Physical Performance Battery^2^ and the sub-scores for the three test items: standing time with the feet in different positions (together, side by side, semi-tandem, tandem and single leg stance) (0-4), 4-metre walk (0-4), and repeated chair stand test (0-4). For these outcomes, the groups will be compared at 3 weeks and 6 months, adjusted for baseline scores.

For the other continuous secondary outcomes (see Appendix 1), the effect of group allocation at the specified time points cognition (3 weeks and 6months after randomisation), self-reported physical activity, activity performance and participation; quality of life, balance confidence and technology self-efficacy (3 and 12 weeks and 6 months after randomisation) will be analysed using linear regression models with baseline scores entered into the linear regression models as covariates. Or if distributions are overly skewed (on visual inspection of histograms) the change scores (post-pre) will be analysed. Mean between-group differences and their 95% CIs will be reported. In addition, self-reported measures of technology usability and enjoyment will be obtained from the intervention group (3 and 12 weeks and 6 months after randomisation).

Fall rates between groups will be compared using negative binomial regression. Interaction terms will be used in the models to assess whether effects of the intervention differed according to age or a neurological versus non-neurological health condition that affects daily activities.

## 4.6 Sub-group analyses

Subgroup analyses will be undertaken on the primary outcomes i.e., physical activity and mobility. All subgroups will be defined by data collected prior to randomisation. Subgroup analysis will be conducted on each of the primary outcomes for:

- Neurological versus non-neurological condition
- Sex (male versus female)
- Age (age entered as continuous covariate and as categorical (above and below median age); both analyses will be considered)
- Baseline mobility level (analysis with 12-point SPPB as a continuous interaction term with data also presented as a dichotomous term above and below median)
- Technology use prior to hospital admission (used vs. not used a computer, tablet, smartphone, pedometer or gaming console in the month prior to hospitalisation)
- State (NSW versus SA)

Unadjusted *p*-values will be reported and the number of declared subgroup analyses will be specified in all publications. The main analysis for each subgroup will be an interaction test in the linear regression model to determine whether the effect of treatment differs significantly across categories for that particular subgroup. The between-group differences within each of the subgroups will be reported, as well as the *p*-value for the interaction test.

## 4.7 Economic evaluation (this will not be reported as part of the main paper)

Health and community service usage data collected from participant-completed calendars, hospital databases and medical records (rehabilitation length of stay, readmissions including type, other settings length of stay e.g. transitional care, number and type of community services at home) will be used to calculate health service utilisation and costs to inform the conduct of an economic evaluation. The cost of the intervention (staff and equipment) will also be calculated from trial records and used in the cost effectiveness analysis. The Incremental Cost-Effectiveness Ratio will be calculated to assess the relative costs and benefits in terms of gain in mobility (the proportion of participants achieving a clinically meaningful improvement on the 12-point SPPB scale) and QALYs.

## 4.8 Exploratory analyses

A sensitivity analysis may be undertaken for the primary outcome ‘Upright time’ including all data regardless whether it meets the *apriori* valid weartime.

Further analyses may be undertaken to explore differential effects by the subgroups outlined above for the secondary outcomes.

Further exploration based on intervention adherence may also be conducted.

## 4.8 Intervention group measures

Adverse events reported in the intervention group, adherence, impression of ‘usability’ and ‘enjoyment’ will be summarised.

**References**

1. Onder G, Penninx BW, Lapuerta P, et al. Change in physical performance over time in older women: the Women's Health and Aging Study. *J Gerontol A Biol Sci Med Sci.* 2002;57(5):M289-293.

2. Guralnik JM, Simonsick EM, Ferrucci L, et al. A short physical performance battery assessing lower extremity function: association with self-reported disability and prediction of mortality and nursing home admission. *J Gerontol.* 1994;49(2):M85-94.

3. de Morton NA, Davidson M, Keating JL. The de Morton Mobility Index (DEMMI): an essential health index for an ageing world. *Health Qual Life Outcomes.* 2008;6:63.

4. Lord SR, Ward JA, Williams P. Exercise effect on dynamic stability in older women: a randomized controlled trial. *Arch Phys Med Rehabil.* 1996;77(3):232-236.

5. Hill K. A New Test of Dynamic Standing Balance for Stroke Patients. *Physiotherapy Canada.* 1996;Fall:257-262.

6. Rehm J, Ustun TB, Saxena S, et al. On the development and psychometric testing of the WHO screening instrument to assess disablement in the general population. *International Journal of Methods in Psychiatric Research.* 1999;8(2):110-122.

7. Delbaere K, Hauer K, Lord SR. Evaluation of the incidental and planned activity questionnaire (IPEQ) for older people. *Br J Sports Med.* 2010;44(14):1029-1034.

8. Powell LE, Myers AM. The Activities-specific Balance Confidence (ABC) Scale. *J Gerontol A Biol Sci Med Sci.* 1995;50A(1):M28-34.

9. Tombaugh TN. Trail Making Test A and B: normative data stratified by age and education. *Arch Clin Neuropsychol.* 2004;19(2):203-214.

10. Merom D, Cumming R, Mathieu E, et al. Can social dancing prevent falls in older adults? a protocol of the Dance, Aging, Cognition, Economics (DAnCE) fall prevention randomised controlled trial. *BMC public health.* 2013;13(1):477.

11. Herdman M, Gudex C, Lloyd A, et al. Development and preliminary testing of the new five-level version of EQ-5D (EQ-5D-5L). *Qual Life Res.* 2011;20(10):1727-1736.

12. Brazier J, Roberts J, Deverill M. The estimation of a preference-based measure of health from the SF-36. *J Health Econ.* 2002;21(2):271-292.

13. Laver K, George S, Ratcliffe J, Crotty M. Measuring technology self efficacy: reliability and construct validity of a modified computer self efficacy scale in a clinical rehabilitation setting. *Disabil Rehabil.* 2012;34(3):220-227.

14. Brooke J. SUS: A “quick and dirty” usability scale. In: P.W. Jordan, B. Thomas, B. A.Weerdmeester, McClelland IL, eds. *Usability evaluation in industry*. London: Taylor & Francis; 1996:189–194.

15. Bangor A, Kortum PT, Miller JT. An empirical evaluation of the system usability scale. *International Journal of Human-Computer Interaction.* 2008;24(6):574-594.

16. Kendzierski D, K. D. Physical activity enjoyment scale: Two validation studies. *Journal of Sport and Exercise Psychology.* 1991;13:50-64.

## Appendix 1: Primary and Secondary Outcome Measures

| *Outcome measures*  *PO: Primary Outcome* | *Time points* | | | *Variable Type* | *Scoring* | *Best outcome* | | | | *Value assignment for participants physically unable to perform tests* |
| --- | --- | --- | --- | --- | --- | --- | --- | --- | --- | --- |
|  | *3*  *wks* | *12 wks* | *6 mths* |  |  | *High* | *Low* | | |  |
| **Balance and mobility** | | | | | | | | | | |
| activPAL | ✓ |  | ✓ | Continuous | Upright time: time spent stepping and standing. | ✓ | |  | Assign 0 | |
| Short Physical Performance Battery (SPPB) - Lower extremity continuous summary performance score (CSPS)^1^ | ✓ |  | ✓ | Continuous | Score 0–3 | ✓ | |  | A value corresponding to the 99th percentile of baseline performance of participants completing the task was assigned to participants who were unable to perform the task or who had a performance below the 99^th^ percentile (walking speed: 11.2 cm/sec). A value corresponding to the 99th percentile of baseline performance of participants completing the task was assigned to participants who were unable to perform the task or who had a performance above the 99th percentile (chair stands: 83 s)  Balance tests: assign 0 | |
| Short Physical Performance Battery (SPPB) + individual components^2^ | ✓ |  | ✓ |  | Total score 0-12  Subscale scores 0-4 | ✓ | |  | Assign 0 for gait velocity standing balance and chair stand if physically unable to do it. | |
| De Morton Mobility Index^3^ | ✓ |  | ✓ | Continuous | Raw score out of 19  DEMMI score between 0-100 | ✓ | |  | Assign 0 | |
| Maximal balance range test^4^ | ✓ |  | ✓ | Continuous | Measured in mm | ✓ | |  | Assign 0 | |
| Step Test^5^ | ✓ |  | ✓ | Continuous | Step count, average of both legs | ✓ | |  | Assign 0  Average of 2 legs. | |
| Single Leg Stance | ✓ |  | ✓ | Continuous | Measured in seconds, up to 10 seconds | ✓ | |  | Assign 0 | |
| **Activity and participation** | | | | | | | | | | |
| WHO Disability Assessment Schedule 2.0^6^ | ✓ | ✓ | ✓ | Continuous | Raw score 12-60, |  | | ✓ | Missing data if not completed | |
| **Physical activity** | | | | | | | | | | |
| Incidental and Planned Exercise Questionnaire^7^ | ✓ | ✓ | ✓ | Continuous | Total score 0-182 | ✓ | |  | Missing data if not completed | |
| **Falls related confidence** | | | | | | | | | | |
| Activities-Specific Balance Confidence Scale^8^ | ✓ | ✓ | ✓ | Continuous | Total score 0-100 | ✓ | |  | Missing data if not completed | |
| **Cognition** | | | | | | | | | | |
| Trails A & B^9^ | ✓ |  | ✓ | Continuous | Difference in time between the two parts. | ✓ | |  | A: unable or score>2 min will be adjusted to 120 s  B: unable or score>5 min will be adjusted to 300 s | |
| **Health-related quality of life** | | | | | | | | | | |
| European Quality of Life-5 dimensions  (EQ-5D-5L)^11^ | ✓ | ✓ | ✓ | Continuous | Each of the 5 dimensions are scored 1-5, each of these scores are assigned a value from the scoring algorithm; and the VAS is scored 0-100 | ✓ | |  | Missing data if not completed (For economic analysis: missing data will be imputed by using the midpoint of previous and following values and carrying forward last observation) | |
| Short Form 6-dimensions questionnaire (SF-6D) calculated from the SF-36^12^ | ✓ | ✓ | ✓ | Continuous | Responses are tallied and converted into the SF-6D utility score determined from a published algorithm | ✓ | |  |  |  |
| **Technology self-efficacy** | | | | | | | | | | |
| Modified Computer Self-Efficacy Scale (MCSE)^13^ | ✓ | ✓ | ✓ |  | Scored 10-100 | ✓ | |  | Missing data if not completed | |
| **Falls** | | | | | | | | | | |
| Number of falls, adverse events, deaths | Monthly | | | Continuous |  |  | | ✓ | Missing data if not available | |
| **Health and community service usage** | | | | | | | | | | |
| Number of contacts with health and community services | Monthly | | | Continuous |  |  | | ✓ | Missing data if not available | |
| **Additional intervention group only measures** | | | | | | | | | | |
| System Usability Scale (SUS)^14,15^ | ✓ | ✓ | ✓ | Continuous | Scored 0-100 | ✓ | |  | Missing data if not completed | |
| Physical Activity Enjoyment Scale (PACES)^16^ | ✓ | ✓ | ✓ | Continuous | Scored 18-126 | ✓ | |  | Missing data if not completed | |

## Appendix 2: ActivPAL data cleaning

As this is our primary outcome and as we are aware that there were differences in how data was entered into REDCap (e.g. whether adjustments were done at the time of data entry), it was decided that all activPAL data should be checked and cleaned as part of the blind review process. Valid weartime was defined apriori as 4 days (24 hour periods).

Data to extract:

- Sit/Lie time (hours)
- Stand time (hours)
- Stepping time (hours)
- Number of steps
- Transitions (use 1^st^ value, number of ‘up’ transitions)
- Energy expenditure (MET.h)

The process for data cleaning was:

1. Open activPAL 3 software. Open the ‘View’ tab at the top and ensure that it is selected ‘View by week’ and “Analysis by 24 hour period. Using the file tab, select each participants activPAL file at 3weeks and 6 months. A one page file should open with graphs on it.
2. Check ‘Elapsed time’ at top of screen to confirm how many days the device collected data (should be 7 days).
3. Visually inspect the Summary by week bar graph (1^st^ graph) to determine if it looks as if device has been worn for total time data was collected. If it appears that the device was not worn of 1 or more 24 hour period (i.e. no steps or uptime recorded) view the detailed graph for that day to determine if the device was worn. If when looking at the summary graph it looks as if 1 or more 24 hour periods is considerably lower than the other days, again inspect the detailed graph of that day. If there is activity at different times through the day then it is likely they were wearing it but were inactive. If there was some activity for the first few hours and then nothing, then it is likely it fell off or was removed. In these scenarios only include the 24 hour periods when there was full data.
4. Below are some scenarios to describe what to do for each:
   1. Scenario 1: device data collection period 7x24 hours but device weartime 3x 24 hours (Figure). This data cannot be included in the analysis and should be removed with note in comments section in REDCap.
   2. Scenario 2: device data collection period 7x24 hours but device weartime just over 4 days (Figure b). Do not use summary values from Summary by week graph. Use sum of daily values for 4 valid 24 hour periods. Make note in comments section in REDCap.
   3. Scenario 3: device data collection period 4 days, 19 hours, 2 mins, 41 secs; weartime the same (Figure c). Do not use summary values from Summary by week graph. Use sum of daily values for 4 valid 24 hour periods. Make note in comments section in REDCap.
   4. Scenario 4: device data collection period 7x24 hours and weartime the same (Figure d). Use summary values from Summary by week graph.

**Figure a: 7 day data collection, 3 day valid weartime**


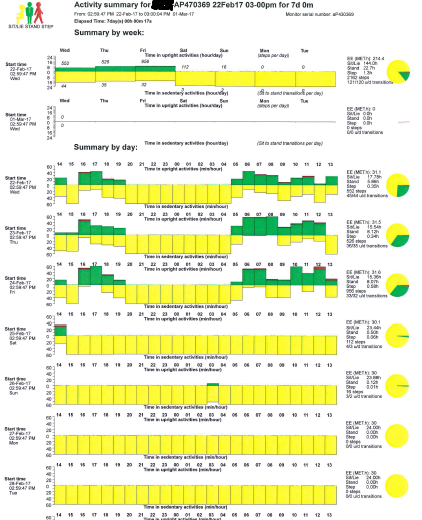


**Figure b: 7 day data collection, 4 day + 2hr valid weartime**

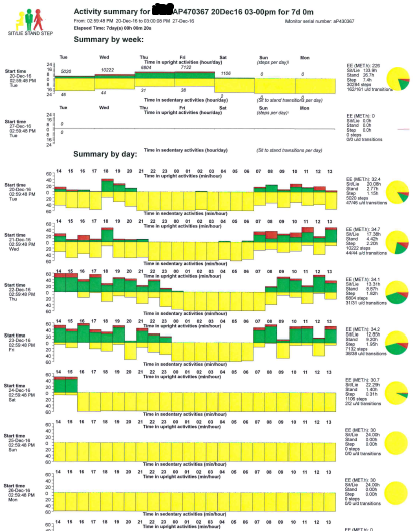


**Figure c: Data collection and weartime = 4 days 19 hours 2 minutes**


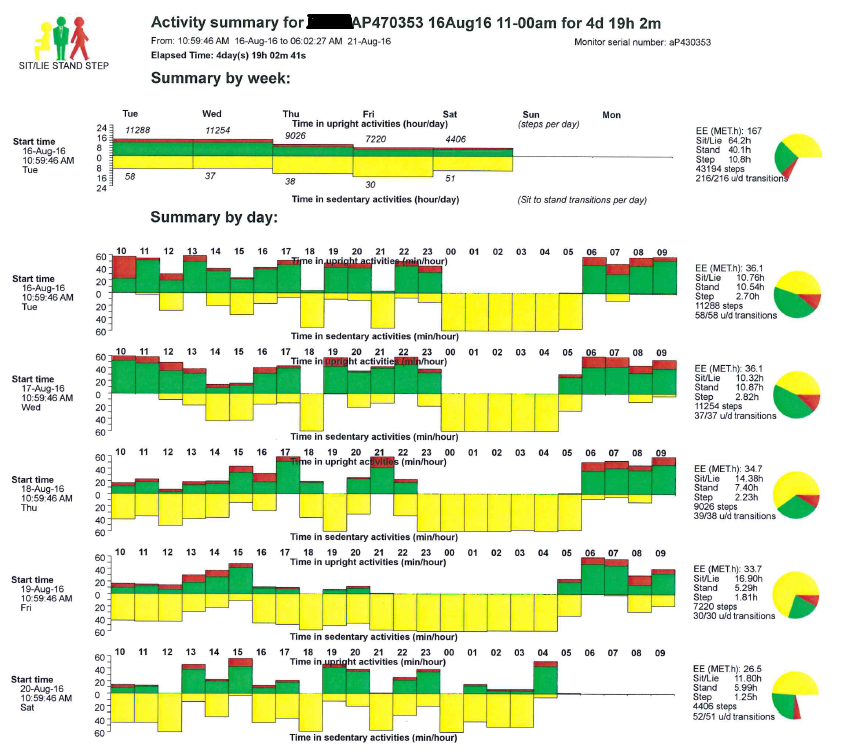


**Figure d: 7 day data collection, 7 day valid weartime**


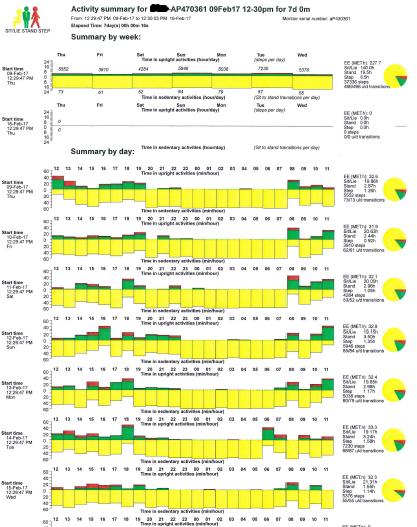

Supplement: S3 Text — (DOCX) [file pmed.1003029.s011.docx]
