# Supplementary material for: Digitally enabled aged care and neurological rehabilitation to enhance outcomes with Activity and MObility UsiNg Technology (AMOUNT) in Australia: A randomised controlled trial
Source: PLoS Med. 2020 Feb 18;17(2):e1003029. doi: 10.1371/journal.pmed.1003029 (PMC7028259; doi:10.1371/journal.pmed.1003029)
Supplement: Attachment — Submitted filename: Response to Reviewers.doc [file pmed.1003029.s012.doc]

**Manuscript: Digitally-enabled rehabilitation to enhance outcomes: The Activity and MObility UsiNg Technology (AMOUNT) randomised controlled trial."**

**(PMEDICINE-D-19-02056)**

**Response to editor and reviewer comments**

**Editor comments to Authors:**

1. PLOS Medicine requires that the de-identified data underlying the specific results in a published article be made available, without restrictions on access, in a public repository or as Supporting Information at the time of article publication, provided it is legal and ethical to do so. PLOS Medicine does not allow authors to be the primary contact for data access.

**Author Response:** We have been advised by our institution repository to contact the lead ethics committee to clarify if we have clearance to do this. If they approve this, we will make our de-identified data available in our institution repository at the University of Sydney at the time of publication.

2. The title could be a bit more descriptive, specifying the type of rehabilitation.

**Author Response:** The type of rehabilitation has been further specified in the title.

**Alterations to Manuscript: Line 1:** “Digitally-enabled aged care and neurological rehabilitation to enhance outcomes: The Activity and MObility UsiNg Technology (AMOUNT) randomised controlled trial.”

3. Please combine Methods and Findings sections of Abstract into one section, “Methods and Findings”.

**Author Response:** The methods and findings sections of the abstract have been combined.

4. Around line 105, please include a new sentence summarizing the secondary outcome findings (from lines 315-328); and a second new sentence summarizing the situation with adverse events, falls and deaths.

**Author Response:** The secondary outcome findings, adverse events, falls and death data have been summarised and included in the abstract findings.

**Alterations to Manuscript: Line 114: “….**Scores were higher in the intervention group compared to the control group across most secondary mobility outcomes but there was no evidence of a difference between groups for most other secondary outcomes. No adverse events were reported in the intervention group. Thirteen participants died while in the trial (intervention group: 9; control group: 4) due to unrelated causes and there was no evidence of a difference between groups in fall rates (unadjusted IRR 1.19 (95% CI 0.78 to 1.83), p=0.43). Study limitations include…”

5. Please edit your language to be a bit more measured, e.g. at line 108 "In this study, we observed improved mobility in people with a wide range of health conditions making use of digitally-enabled rehabilitation, whereas time spent upright was unchanged."

**Author Response:** This sentence has been edited.

**Alterations to Manuscript: Line 121: “Conclusions:** In this study, we observed improved mobility in people with a wide range of health conditions making use of digitally-enabled rehabilitation, whereas time spent upright was not impacted.”

6. In the Methods section, please specify if written or verbal consent was obtained from the study participants, or if the ethics committee waived this requirement.

**Author Response:** Written consent was obtained from all study participants. Please see line 215. “Following written informed consent and baseline assessment...”

**Alterations to Manuscript:** No alteration to manuscript.

7. Lines 259-261(“Role of the Funding source”) can be removed.

**Author Response:** This section has been removed from the manuscript.

8. Please briefly describe intervention and trial at beginning of the Discussion.

**Author Response:** A brief description of the trial and intervention has been added to the beginning of the discussion.

**Alterations to Manuscript: Line 430: “**We conducted a pragmatic, assessor-blinded, parallel-group randomised trial in people with mobility limitations undertaking aged care and neurological rehabilitation recruited from three Australian hospitals to investigate whether tailored prescription of affordable digital devices (including virtual reality videogames, activity monitors and handheld computer devices) in addition to usual care could improve mobility and physical activity when compared with people undertaking usual care alone. ….”

9. Line 355, please rephrase as "In this study, significant and clinically relevant improvements in mobility were observed in participants receiving the AMOUNT intervention ..." or similar.

**Author Response:** We have edited our sentence to include the suggested rephrasing.

**Alterations to Manuscript: Line 437: “**However, significant and clinically relevant improvements in mobility were observed in participants receiving the AMOUNT intervention.”

10. At line 391, "we improved" seems too strong, please temper.

**Author Response:** This sentence has been edited.

**Alterations to Manuscript: Line473:** “Although the physical capacity of participants in the intervention group to move around improved, this did not translate to increased time spent upright.”

11. At line 424, please rephrase to "... we observed improved mobility in participants in a digitally-enabled rehabilitation environment ...".

**Author Response:** This sentence has been edited.

**Alterations to Manuscript: Line 507:** In summary, we observed improved mobility in participants with a wide range of health conditions in a digitally-enabled rehabilitation environment, but no between-group differences in upright time.

**Reviewer 1 Comments to Authors:**

Reviewer #1: Thank you for the opportunity to review this very interesting RCT evaluating a complex intervention. The authors have conducted a pragmatic trial to evaluate whether digital-enabled rehabilitation can improve mobility and upright time in patients admitted to age and neurological care in hospital settings. Over a six month period, the intervention improved mobility but not upright time. Overall, the analyses were appropriate and followed the pre-specified statistical analyses plan. My comments are specified below:

1. Lines 190-191: Primary outcomes on SPPB - it's not clear why the commonly used 12-point score was not utilised as the primary outcome compared to the 0-3 score. I would think that a 12-point scale would have more variation to allow for movement in the outcome and hence may be able to detect larger changes. The analyses shows this as both the 0-3 score and 12-point score do show significant differences between groups with the different larger in the 12-point score.

**Author Response:** The *a priori* decision to use the continuous 3-point version rather than the categorical 12-point version of the SPPB was due to our hypothesis that the responsiveness of the continuous version may be greater due to the avoidance of categorisation. For example, a person would score 2/4 for the walking sub-scale of the SPPB if they scored between 8.70 and 6.21 seconds. So, any change in walking speed between 8.70 and 6.21 seconds would not be picked up on the 12-point scale. The 3-point continuous version uses the actual walking speed and so will detect any change, even if small, in the walking speed. The same is true for the standing balance and sit to stand components. Our decision was based on investigator experience of using this instrument in previous work (van den Berg M. et al 2015, *JOP*), clinical experience in rehabilitation, and the paper published by Onder G. et al, 2002 in the *J. Gerontol*.

**Alterations to Manuscript:** No alterations to manuscript.

2. Lines 195-198: Primary outcome on activPal - This outcome has been averaged across a 7-day period. I would expect that the daily monitoring would have some large variations relatively which doesn't not necessarily follow a normal distribution. It's also likely reason why it's an average across seven days is not going to capture the variation in the differences between groups.

**Author Response:** We see the reviewer’s point, but it is generally considered best practice to objectively measure physical activity for a period of 7-days, define valid wear time (for AMOUNT acceptable wear time was set *a priori* and defined as 4 days, for 24 hours per day) and to report the outputs as average per day (Matthews C.E. 2012; *MSSE)*. This is because, as the reviewer notes, there is likely to be intra-participant variability from day-to-day as to how much physical activity is conducted and so the average over a few days to a week is likely to be a better reflection of current physical activity levels.

**Alterations to Manuscript:** No alterations to manuscript.

3. Outcomes section in methods: The co-primary outcomes although stated in the abstract and findings are described as a mean difference between groups, there is some nuance between the mobility outcome and upright time outcome which could help with better clarity in the methods. The mobility outcome (SPPB) is a mean difference of the relative change from baseline (i.e. difference in difference) between groups whereas the activPAL was only the mean difference between groups at a point prevalence at six months.

**Author Response:** This is correct. As per our Statistical Analysis Plan we stated, “The distribution of continuous variables will be evaluated to inform whether change scores will be used for analysis.” Most continuous variables were skewed and therefore change scores were used for most analyses. For the co-primary outcomes, uptime was not skewed and therefore the analysis was the between group difference at 6-months. The SPPB continuous score was skewed and therefore the analysis was the mean difference of the change from baseline to 6-months between groups. In both instances, the analysis was adjusted for baseline values. The key under Table 5 states this (“Due to skewed distributions, the change score between timepoints was used for all outcomes except proportion of the day spent upright), however we have added a sentence to the methods section to further clarify this point.

**Alterations to Manuscript: Line 317: Methods, data analysis:** “Between-group comparisons for continuously scored outcomes were made using linear models with baseline scores entered as covariates. The distribution of continuous variables was evaluated to inform whether change scores were used for analysis……..”

4. Related to point 3) as the tables clearly show that activPAL was also assessed at baseline, did the authors look at the different in the mean difference between groups relative to baseline similar to the method used in analysing SPPB.

**Author Response:** We viewed the distribution of the data prior to commencing the analysis to determine if change scores were required. As the activPAL data distribution was not skewed, we did not use change scores. The mean difference between groups was adjusted for baseline values for all measures that were measured at baseline. The key underneath Table 5 states this. “This analysis was conducted using linear regression models with baseline scores entered as covariates.”

**Alterations to Manuscript:** No alterations to manuscript.

5. The outcome of the upright time has been averaged across a 7-day period for daily time. Was the daily activity time relatively stable as within each individual - as I would expect there is probably some variation which doesn't not necessarily follow a normal distribution? It may be also the reason that the average doesn't capture the nature of the change in this outcome. One suggestion could be look at this as a repeated measure analyses of average per-day between intervention and control groups.

**Author Response:** Participants were asked to wear the activPAL activity monitor for 24 hours a day for 7 days. The monitor was attached to the thigh with a thin transparent wound dressing material and was not removed until the end of the 7-day period. We aimed to improve total upright time, so we needed to look at a representative estimate of the usual upright time. As per our response to comment 2 above, it is generally considered best practice to report the outputs as average per day. We applied the same approach to all activPAL outcomes.

**Alterations to Manuscript:** No alterations to manuscript.

6. Lines 226-227: It's not clear if falls and health community service usage were also part of adverse events monitored.

**Author Response:** Adverse events were only monitored in the intervention group as they were defined as an unwanted and usually harmful outcome (e.g., fall, seizure, cardiac event) that may or may not have been related to the intervention, but occurred while the participant was undertaking mobility or physical activities using intervention digital devices. Falls and service utilisation were measured for both groups with falls rates compared between groups (Supplementary Table 5) and service utilisation will be compared between groups as part of the trial economic analysis (reported separately). We have added a sentence to the Methods section to define adverse events.

**Alterations to Manuscript: Methods: Line 296: Outcome measures**: *Secondary outcomes:* “ Adverse events in the intervention group and deaths in both groups were monitored and documented throughout the trial. Adverse events were defined as an unwanted and usually harmful outcome (e.g., fall, seizure, cardiac event) that may or may not be related to the intervention, but occurred while the participant was undertaking mobility or physical activities using intervention digital devices………”

7. Lines 246-247: this needs justification using a NBR rather than a Poisson regression. I can venture a guess that a negative binomial model is expected to provide an improved fit to the data and accounted better for over-dispersion (variance is high compared to mean rates) than the Poisson regression model, but authors to clarify.

**Author Response:** The reviewer is correct that NBR is commonly used for analysis of falls data due to over-dispersions. This was also the case in our analysis as tested as part of the NBR command in Stata.

**Alterations to Manuscript:** No alterations to manuscript.

8. Consort diagram: I'm going to assume that the n = 129 included in the analysis on the consort was the number analysed for intention to treat - would be helpful to indicate this in the consort diagram with an * footnote.

**Author Response:** We have made this change as suggested.

**Alterations to Manuscript: Line 349: Figure 1:** * Number included in intention-to-treat analysis

9. Table 2: It would be helpful to see in this baseline characteristics table whether any of the baseline factors were significantly different. There may be some slight variation between intervention and control just on quick view of the table (i.e. proportions for prior living arrangements, primary diagnosis grouping proportions. also intervention group looks like they are experience using devices in month prior to hospital). If some of these factors may be different - it could justify baseline covariate adjustment in the analysis of between group differences as if randomisation didn't completely control for confounding then these factors still may influence the results.

**Author Response:** We understand your suggestion to ensure we have accounted for influencing factors in our analysis. However, it is our understanding that current reporting guidelines do not recommend statistical testing of baseline values between-groups due to the risk of spurious findings (Moher D, et al. CONSORT 2010 Explanation and Elaboration: updated guidelines for reporting parallel group randomised trials, *Journal of Clinical Epidemiology* 63 (2010) e1-e37).

**Alterations to Manuscript:** No alterations to manuscript.

My overall impression is that this is a well-conducted, interesting, and important piece of work that warrant publication. The authors have stated that the economic analyses will be presented as a separate piece, but I would encourage the authors to consider including the cost-effectiveness alongside this RCT as it would help with understanding the value for money of this intervention which would greatly enhance this piece. I see that as a key strength but ultimately up the authors. I hope the authors find the comments useful to help improve their hard work prior to publication.

**Author Response:** We thank the reviewer for their carefully considered and positive review of our manuscript. We appreciate the suggestion of presenting the economic analysis alongside the trial results. However, due to limited space in a journal paper and the details we have in our economic analysis, we would like to stay with our plan of presenting it separately.

**Reviewer 2 Comments to author:**

Reviewer #2: It was a pleasure to review this well conducted trial evaluating an important clinical intervention, namely the use of digital technology to enhance rehabilitation across the ward and post-discharge settings. The inclusion of the intervention protocol and quality of intervention reporting in the manuscript is commendable. Overall the manuscript is well-written, but I have outlined a number of areas that could be addressed to improve precision in the trial reporting. I would encourage revision of the manuscript and I hope my comments are useful to the authors in their endeavours to report this clinically relevant trial.

**Author Response:** We thank the reviewer for their detailed and helpful comments in revising our manuscript to enhance the reporting of our trial.

ABSTRACT

1. It would be useful to briefly explain what usual care is to provide context.

**Author Response:** A sentence has been added to the abstract to briefly explain what usual care is. Further detail is provided in Table 1 (Participants were: (1) provided with a fall prevention brochure on discharge from hospital; (2) provided with inpatient usual care at the three study sites involving assessment and prescription of a series of repetitive exercises (e.g. practice of standing up or stepping). Usual care also included assessment and tailored management by medical specialists, nurses, occupational therapists, speech pathologists, social workers, nutritionists, orthoptists and other health professionals as required; (3) referred to usual outpatient therapy as clinically required).

**Alterations to Manuscript: Line 92: “Methods and findings:** We conducted a pragmatic, outcome assessor-blinded, parallel-group randomised trial in three Australian hospitals recruiting adults with mobility limitations undertaking aged care and neurological inpatient rehabilitation. Both groups received usual multidisciplinary inpatient and post-hospital rehabilitation care as determined by the treating rehabilitation clinicians. In addition to usual care, the intervention group…”

2. The intervention would benefit from some more specific information as it was more involved than just prescribing devices.

**Author Response:** Further detail describing the intervention has been added to the abstract.

**Alterations to Manuscript: Line 96: “….**In addition to usual care, the intervention group used devices to target mobility and physical activity problems, individually-prescribed by a physiotherapist according to an intervention protocol, including virtual reality videogames, activity monitors and handheld computer devices for 6 months in hospital and at home.

3. CONSORT guidance for trial abstracts recommends specifying how participants were allocated to the interventions, further detail would be beneficial.

**Author Response:** The randomisation schedule was prepared by a researcher not involved in the conduct of the study and was generated on the computer and was embedded into the trial REDCap database to enable allocation concealment. Further detail describing that randomisation used allocation concealment has been added to the abstract.

**Alterations to Manuscript: Line 102: “**Between September 22, 2014 and November 10, 2016, 300 patients (mean age 74, SD 14; 50% female) were randomly assigned with allocation concealment to intervention (n=149) or control (n=151).”

4. Suggest adding 'outcome' before 'assessor-blinded' to aid clarity.

**Author Response:** We have made this change as suggested.

**Alterations to Manuscript: Line 92:** “….We conducted a pragmatic, outcome assessor-blinded, parallel-group randomised trial in three Australian hospitals.”

5. More information on the eligibility criteria is needed to understand which patient groups this trial would have implications for.

**Author Response:** The main trial eligibility criteria has been added to the abstract.

**Alterations to Manuscript: Line 92: “Methods and findings:** We conducted a pragmatic, outcome assessor-blinded, parallel-group randomised trial in three Australian hospitals recruiting adults with mobility limitations undertaking aged care and neurological inpatient rehabilitation.”

6. Results for each group are needed with the mean-difference and Cis.

**Author Response:** Values for primary co-primary outcomes have been added to the abstract.

**Alterations to Manuscript: Line 108:** Change in mobility scores were higher in the intervention group compared to the control group from baseline (SPPB (continuous, 0-3) mean (SD) intervention group: 1.5 (0.7); control group: 1.5 (0.8)) to 6 months (SPPB (continuous, 0-3) mean (SD) intervention group: 2.1 (0.6); control group: 1.8 (0.8); mean between-group difference 0·2 points; 95%CI 0·1 to 0·3; p=0·006), however there was no evidence of a difference between groups for upright time at 6 months (mean (SD) min/day at 6 months intervention group: 262 (142); control group: 265 (147); mean between-group difference -0·2, 95%CI -2·7 to 2·3; p=0·87).

7. Suggest alternative wording is used for the results, 'significantly' can be ambiguous - statistically or clinically significant, or both? Suggest 'SPPB scores were higher in the intervention group compared to the control group' (or other wording to that effect).

**Author Response:** The wording has been modified as suggested.

**Alterations to Manuscript: Line 108: “**Change in mobility scores were higher in the intervention group compared to the control group…”

8. Rather than 'similar between groups', suggest more precise statistical wording such as 'no evidence of a difference between groups' (or other wording to that effect)

**Author Response:** The wording has been modified as suggested.

**Alterations to Manuscript: Line 111:** “….however there was no evidence of a difference between groups for upright time at 6 months…”

9. Harms need to be reported, in line with CONSORT.

**Author Response:** The following information has been added to the abstract.

**Alterations to Manuscript: Line 116: “….**No adverse events were reported in the intervention group. Thirteen participants died while in the trial (intervention group: 9; control group: 4) due to unrelated causes and there was no evidence of a difference between groups in fall rates (unadjusted IRR 1.19 (95% CI 0.78 to 1.83), p=0.43).”

10. Suggest removing 'not unexpected in this group of hospitalised patients' as this is more a discussion point.

**Author Response:** This has been removed.

**Alterations to Manuscript: Line 119:** “Study limitations include an anticipated 15-19% loss to follow-up at 6 months on co-primary outcomes.”

11. Conclusion - Were the improvements in SPPB clinically important?

**Author Response:** We suggest that the improvements in SPPB (continuous) represent a clinically important difference. The between group difference of change scores from baseline to 3 weeks and baseline to 6 months in favour of the intervention group represent a clinically important difference based on previous literature. This has been discussed in the Discussion section of the manuscript.

**Alterations to Manuscript:** No alterations to manuscript.

12. Trial registration number is accurate and consistent with the submitted report

**Author Response:** No response required.

MAIN PAPER: Introduction:

13. 114-15: References to support this more general statement relate to stroke, whereas the preceding sentence suggests this statement is about 'neurological and musculoskeletal health conditions'. References need revisiting or the statement made more precisely.

**Author Response:** Two additional references have been added to support the statement for people with non-neurological health conditions:

Reference 6: Sherrington, C., Michaleff, Z. A., Fairhall, N., Paul, S. S., Tiedemann, A., Whitney, J., Cumming, R. G., Herbert, R. D., Close, J. C. & Lord, S. R. 2017. Exercise to prevent falls in older adults: An updated systematic review and meta-analysis. *Br J Sports Med*, 51, 1750–8.

Reference 7: Olivetti L, Schurr K, Sherrington C, et al. A novel weight-bearing strengthening program during rehabilitation of older people is feasible and improves standing up more than a nonweight-bearing strengthening program: a randomised trial. *Aust J Physiother* 2007;53:147–53.

**Alterations to Manuscript: Line 163:** “Many will need accessible and affordable rehabilitation to reduce costly limitations in function from neurological and musculoskeletal health conditions [2] as well as decline from aging and inactivity [3]. Physical rehabilitation should contain intensive repetitive task-specific exercises to improve outcomes [] [4-7].

14. 117-8: what is the hypothesised mechanism by which a digitally enhanced environment enables more practice? Behaviour change? What is the evidence for this mechanism?

**Author Response:** Digitally-enabled rehabilitation is defined as rehabilitation using digital devices (e.g. virtual reality, wearables, and tablet and smartphone applications) with remote monitoring and communication. The dose of practice can be increased by using devices that are enjoyable, engaging and motivating and can be used in the hospital and home setting, and for some patients, unsupervised. The quality of the practice can be enhanced through the devices providing feedback to facilitate motor learning, enable self-monitoring of performance and provide challenge to the cognitive as well as physical system. Rehabilitation dose can be continued in the home setting with many devices enabling remote monitoring of exercise adherence and ongoing communication for longer-term goal achievement and behaviour change. The manuscript has been modified to provide further detail and references.

**Alterations to Manuscript: Line 167:** “Virtual reality video games, activity monitors and handheld computer devices are accessible, affordable and enjoyable, [8] and together can provide a digitally-enabled rehabilitation environment by providing more opportunity and greater motivation to increase task-specific practice in hospital [9] and in the home setting [10].

Reference 9: Betker AL, Desai A, Nett C, Kapadia N, Szturm T. Game-based exercises for dynamic short-sitting balance rehabilitation of people with chronic spinal cord and traumatic brain injuries. Physical Therapy. 2007;87(10):1389-98.

Reference 10: Allen NE, Song J, Paul SS, Smith S, O'Duffy J, Schmidt M, et al. An interactive videogame for arm and hand exercise in people with Parkinson's disease: A randomized controlled trial. Parkinsonism Relat Disord. 2017;41:66-72.

15. 126 - sentence tense correction needed 'requires' to 'required'

**Author Response:** The sentence tense has been corrected.

**Alterations to Manuscript: Line 179:** This promising intervention, after refinement, now required rigorous evaluation.

16. 123-5 - which population was the feasibility assessed in and why this group(s)?

**Author Response:** The feasibility trial was conducted in the same population as the present trial, people undertaking aged care and neurological rehabilitation. As for the current study, this population was targeted due to the growing need for accessible and affordable rehabilitation to maximise function in people with neurological and musculoskeletal health conditions and people with deconditioning from the ageing process. Information on which population group was included in the feasibility study has been added to the manuscript.

**Alterations to Manuscript: Line 175: “** A feasibility trial conducted by our team (n=58) in people undertaking inpatient aged care and neurological rehabilitation provided an additional dose of rehabilitation for 2-weeks using a range of low-cost videogames and activity monitors [9].

17. 137 - it can be appreciated that the full economic evaluation may be reported later but the cost of delivery would be useful information to present with the main trial results to provide greater context. Not essential but would enhance the report.

**Author Response:** This information is being prepared for the economic analysis paper and we would prefer to report these data in the economic analysis paper.

**Alterations to Manuscript:** No alterations to manuscript.

18. 146 - what was the trial training? What steps were taken to ensure selection bias was managed (any external audit/screening)?

**Author Response:** All staff received training prior to commencing on the trial. This included training in trial processes (screening, recruitment, conducting baseline assessments, data entry etc) as well as delivery of the intervention (including using the intervention protocol). The Research Managers monitored trial processes throughout the trial including recruitment (e.g. quarterly reviews of screening summaries), data entry and provision of intervention (e.g. clinical reasoning sessions). Trial budget was not sufficient for any external audits to be conducted. The manuscript has been modified to provide further detail.

**Alterations to Manuscript: Line 197:** “Research physiotherapists recruited participants, conducted baseline assessments, randomised participants and delivered the intervention; all were experienced physiotherapists and received training in trial processes, as well as intervention training addressing elements summarised in Table 1.”

19. 149-50 - 'clinician assessed capacity for improvement' - how was this assessed? It appears possible that more complex patients could have been systematically excluded? It would be good to report all of the categories for eligibility in the CONSORT rather that the few selected to assess which of these criteria dominated (if any)

**Author Response:** The treating physiotherapist was consulted as to the patient’s capacity for improving their mobility. It was a clinical decision based on the physiotherapist’s clinical experience and their assessment and treatment experience with the patient. As part of the inclusion/exclusion criteria patients were only eligible if they did have some limitation in their mobility (assessed as <12/12 on SPPB scale) but were able to maintain a standing position (with assistance of one person if necessary). The mean (SD) mobility at baseline assessed on the SPPB was 4.3 (2.6) for the intervention group and 4.2 (2.6) for the control group indicating participants were recruited into the trial with substantial mobility limitation.

The current CONSORT diagram presented as Figure 1 includes the top three reasons for exclusion: Not meeting inclusion criteria: n=4324: 1) anticipated LOS < 10 days n=1580; 2) cognition n=701; 3) mobility n=404; 4) other n=1639. Other included: not community dwelling (n=375); outside geographical area (n=306); insufficient English (n=250); Not medically fit to exercise (n=179); 1+assist in standing (n=139); not interested in technology (n=122); palliative (n=118); visual impairment (n=85); SPPB=12 (n=32); participant in another trial (n=27); rehabilitation re-admission already in AMOUNT trial (n=4); <18 years old (n=2).

We have edited the exclusion box in the CONSORT diagram (Figure 1) to state the Top 5 reasons for exclusion.

**Alterations to Manuscript:** Fig 1 (excluded box in Figure 1)

Excluded (n=4739)

- Not meeting inclusion criteria

(n=4324: Top 5 reasons: anticipated LOS<10 days n=1580; cognition n=701; mobility n=404; not community dwelling n=375; outside geographical area n=306)

- Declined to participate (n=415)

20. 155 - what is the definition of a 'high care residential facility' (this is country specific so would be useful context)

**Author Response:** A high care residential facility is equivalent to a nursing home or aged care facility. We have added “nursing home” to the manuscript to assist with broader understanding.

**Alterations to Manuscript: Line 209:** “..anticipated discharge to high care residential facility (nursing home)…”

21. 165 - The Authors are commended for the reporting of the intervention. Suggest the description of usual care would be useful in the main text in a summary sentence, and a signpost to Table 1, before going onto the intervention.

**Author Response:** Further detail has been added to the text and readers signposted to Table 1.

**Alterations to Manuscript: Line 220:** “**Intervention** Both groups received usual rehabilitation care which was determined by the treating clinicians and included assessment and prescription of a series of repetitive exercises by the physiotherapist, tailored management by the multidisciplinary team, plus a falls prevention brochure [12] (see Table 1).

22. 172 - expand on 'contextual factors'

**Author Response:** Personal and environmental factors were taking into account when prescribing devices. Further detail has been included in the manuscript.

**Alterations to Manuscript: Line 227:** “The exercises and devices were individually-prescribed by a trial physiotherapist according to an intervention protocol which matched different task-specific exercises on different devices to common mobility limitations. The physiotherapist also considered participant impairments (e.g. upper limb weakness, hemianopia) and contextual factors such as participant goals, device preferences and the home environment.”

23. How did the interventions and protocol ensure sufficient dose and task-specificity? Some information is in the Table 1 and other files but this is important in the main text as it is difficult to interpret the main text independently.

**Author Response:** The protocol was developed by the Investigator team with consumer input after trialling different devices and playing the different games or doing the different exercises to determine if they provided (or if they could be modified) to enable task-specific practice of mobility tasks and/or increase physical activity. The intervention protocol specified participants should aim for 30-60 minutes of additional practice using digital devices on top of their usual rehabilitation. Trial physiotherapists recorded total session durations and where possible repetitions and game time as a way of monitoring the dose. We have added further detail to the manuscript.

**Alterations to Manuscript: Line 223:** **“**In addition, the intervention group received 30 to 60 minutes of digitally-enabled rehabilitation five days per week ………..The exercises and devices were individually-prescribed by a trial physiotherapist according to an intervention protocol which matched different task-specific exercises on different devices to common mobility limitations. The physiotherapist also considered participant impairments (e.g. upper limb weakness, hemianopia) and contextual factors such as participant goals, device preferences and the home environment.”

24. 181 - what was the justification for these co-primary outcomes?

**Author Response:** The intervention was developed to improve mobility broadly and to improve overall physical activity. Upright time was chosen as our primary measure of physical activity rather than step count due to the fact that our inclusion criteria included some people unable to walk but all who were able to stand at study entry. The SPPB was chosen as it measures standing balance, sit to stand and walking and could be measured in hospital and at home.

**Alterations to Manuscript:** No alterations to manuscript.

25. 192-93 - it is not clear why a different version of the SPPB was selected. The reference cited (Onder) does not seem to explicitly provide strong evidence that supports using this alternative calculation to the otherwise established version - please can the authors expand on this?

**Author Response:** The *a priori* decision to use the continuous 3-point version rather than the categorical 12-point version of the SPPB was due to our hypothesis that the responsiveness of the continuous version may be greater due to the avoidance of categorisation. For example, a person would score 2/4 for the walking sub-scale of the SPPB if they scored between 8.70 and 6.21 seconds. So, any change in walking speed between 8.70 and 6.21 seconds would not be picked up on the 12-point scale. The 3-point continuous version uses the actual walking speed and so will detect any change, even if small, in the walking speed. The same is true for the standing balance and sit to stand components. Our decision was based on investigator experience of using this instrument in previous work (van den Berg M. et al 2015, *JOP*), clinical experience in rehabilitation, and the paper published by Onder G. et al, 2002 in the *J. Gerontol*.

**Alterations to Manuscript:** No alterations to manuscript.

26. 227-8 - how were adverse events reported? There is some information in the protocol on definitions but are these self-reported or clinician reported, or both? Differences in levels in clinical contact can influence this event reporting.

**Author Response:** Adverse events were only monitored in the intervention group as they were defined as an unwanted and usually harmful outcome (e.g., fall, seizure, cardiac event) that may or may not have been related to the intervention, but occurred while the participant was undertaking mobility or physical activities using intervention digital devices. Falls were measured for both groups with falls rates compared between groups (Supplementary Table 5). We have added a sentence to the Methods section to define adverse events.

**Alterations to Manuscript: Methods: Outcome measures**: **Line 296:** “ Adverse events in the intervention group and deaths in both groups were monitored and documented throughout the trial. Adverse events were defined as an unwanted and usually harmful outcome (e.g., fall, seizure, cardiac event) that may or may not be related to the intervention, but occurred while the participant was undertaking mobility or physical activities using intervention digital devices………”

27. 236 - why was a 15% between group difference in both measures specified? What were the underlying assumptions on clinically important differences in effects?

**Author Response:** This was the authors’ estimate of meaningful differences on the basis of our collective clinical experience with the measures**.**

**Alterations to Manuscript:** No alterations to manuscript.

28. Sample size: It is not clear how the presence of co-primary outcomes was accounted for in the sample size calculation. With co-primary outcomes it is usually assumed that both endpoints need to demonstrate meaningful differences, which reduces power and increases type 2 error. If the sample sizes were calculated separately at 90% power, this would in effectively be 81% power over the two outcomes (0.9 x 0.9 = 0.81). This is likely to be less of an issue as the two endpoints are likely correlated but this section would benefit from further detail, for example, was there any sample size inflation to manage this? I could not find this detail in the protocol, registration, SAP or published protocol.

**Author Response:** The sample size was not inflated for the co-primary outcomes as our analysis plan stated: “P-values were not adjusted for multiplicity as we pre-specified that a significant effect must be observed on both primary outcomes to declare the intervention effective.”

**Alterations to Manuscript:** No alterations to manuscript.

29. 244 - 'intention to treat' - has this trial used a modified ITT? It looks like it was not a strict ITT as not all participants were included in the final analysis regardless withdrawals or lost to follow-up, please confirm.

**Author Response:** Follow-up was attempted on all participants (apart from those who were deceased) regardless of whether they wished to go ahead with the intervention. We have undertaken analyses on available data and baseline group allocation regardless of intervention uptake. We consider this to be an ITT analysis, but we accept that others use different definitions.

**Alterations to Manuscript:** No alterations to manuscript.

30. 247 - please specify if the sensitivity analyses were pre-specified

**Author Response:** This change has been made.

**Alterations to Manuscript: Line 320:** “Two pre-specified sensitivity analyses were conducted…”

31. Were there any protocol amendments? If so, these should be included in the report in line with CONSORT.

**Author Response:** There were six protocol versions prior to the final published version. Four of the six versions were prior to recruitment commencing. A summary of the protocol amendments has been added to the Supplementary Appendix (and included at the end of this document) and a sentence added to the manuscript. There were a few minor modifications to the intervention protocol and these have been reported in Table 1 as part of the TIDieR checklist.

**Alterations to Manuscript: Line 332: “IRB Approval:** Two human research ethics committees (HREC) approved the trial (Southern Adelaide Clinical HREC and South Western Sydney Local Health District HREC). Six minor protocol amendments were approved by the ethics committees, four prior to the trial commencing (see the supplementary material).”

32. Much greater detail is needed on the strategies to blind assessors, how were the outcome measures assessed, what safeguards were there?

**Author Response:** This was reported in the protocol paper and was left out of the manuscript due to space limitations. An additional paragraph has been added to the Outcome Assessment section to describe this process including blinding.

**Alterations to Manuscript: Line 239: “Outcome measures: “**Face-to-face outcome assessments were conducted at 3 weeks and 6-months after randomisation and by mail or telephone at 12 weeks after randomisation. Outcome assessors were registered health professionals, trained in conducting the outcome assessments, external to the clinical sites, and blinded to group allocation. The face-to-face assessments were conducted in the hospital if the participant was still an inpatient, or at the post-hospital discharge destination (e.g. home, transitional living unit). The intervention devices were removed from participant homes and participants were reminded not to discuss their trial involvement with the assessor prior to the outcome assessor completing the 6-month assessment.**”**

33. 271/Fig 1 - see earlier comments regarding adding detail to CONSORT on reasons; does the number analysed reflect both primary analyses? 'n=' missing on deceased

**Author Response:** Please see previous response to comment 19 regarding CONSORT reasons. The number analysed (n=258) reflects the total number of participants who had the final 6-month assessments completed. The percentages for the co-primary outcomes are in the results text and the numbers are included in the data tables. Numbers have been added alongside the percentages in the text.

**Alterations to Manuscript: Line 341:** “Six-month assessments were completed by 258 participants (control group: 129/151, 85%; intervention group: 129/149, 87%). For co-primary outcomes, there was an 85% (254/300) follow-up rate for mobility (data unavailable for four additional participants who refused to complete one or more test components) and 80% (239/300) follow-up rate for upright time……”.

34. Very low loss to follow-up, excellent - were there any reasons for the withdrawals?

**Author Response:** Withdrawal reasons were varied but matched the complex patient group we were working with. Reasons for withdrawal included feeling too overwhelmed when discharged home, changing their mind when discharged home, becoming unwell, change in living circumstance and not wanting to fill in more questionnaires.

**Alterations to Manuscript:** No alteration to manuscript.

35. Results - for all percentages, it is informative to provide numerators and denominators

**Author Response:** This has been added to the text.

**Alterations to Manuscript: Line 341: “**Six-month assessments were completed by 258 participants (control group: 129/151, 85%; intervention group: 129/149, 87%). For co-primary outcomes, there was an 85% (254/300) follow-up rate for mobility (data unavailable for four additional participants who refused to complete one or more test components) and 80% (239/300) follow-up rate for upright time….”

36. Table 2 and S1 - balanced baseline characteristics on a whole but note considerable variation in primary condition and age in the trial cohort is evident

**Author Response:** Pre-specified sub-group analyses were included to explore the effect of age and whether or not the person had a neurological health condition.

**Alterations to Manuscript:** No alterations to manuscript.

37. 302-3 - was there any skew in the number of sessions? Often the case in rehab trials where median, IQR, range is needed - would be reassuring to confirm during review

**Author Response:** “We have replaced the mean (SD) with the median (IQR) in Table 4 for number of inpatient sessions offered and number of inpatient sessions delivered due to skewness of this data. The median (IQR) has been indicated in Table 4 with a “^#^” and explanation added to the Table key.

Median (IQR) number of inpatient sessions offered: 11 (16)

Median (IQR) number of inpatient sessions delivered: 7 (10)

**Alterations to Manuscript:** See Table 4, page 26.

38. 309 -14 - as per abstract - please see comments on reporting 'significant'

**Author Response:** “significant” has been removed from results section and just reported whether or not there is was a difference between groups for the outcomes.

**Alterations to Manuscript: Line: 380: “***Co-primary outcomes*

Change in mobility scores were higher in the intervention group compared to the control group from baseline (SPPB (continuous, 0-3) mean (SD) intervention group: 1.5 (0.7); control group: 1.5 (0.8)) to 6 months (mean between-group difference 0·2 points; 95%CI 0·1 to 0·3; p=0·006), however there was no evidence of a difference between groups for upright time at 6 months (mean (SD) min/day at 6 months intervention group: 262 (142); control group: 265 (147); mean between-group difference -0·2, 95%CI -2·7 to 2·3; p=0·87).”

*Secondary outcomes*

**Line: 400:** “There were between-group differences in favour of the intervention group across most secondary mobility outcomes …”

**Line: 404:** “There was no evidence of a difference between groups in the number of steps taken per day from baseline to 6 months in the intervention group..”

**Line: 408:** “The same number of participants reported falling one or more times in both groups (n=53) and there was no difference between groups in fall rates…”

39. Table 5 - footnote states skew was managed - please add to methods section

**Author Response:** This has been added to the Methods section.

**Alterations to Manuscript: Line 317: “**Between-group comparisons for continuously scored outcomes were made using linear models with baseline scores entered as covariates. The distribution of continuous variables was evaluated to inform whether change scores were used for analysis….”

40. 325 - secondary outcomes section - with the risk of multiplicity, suggest the focus on evidence and avoiding interpretations, such as 'substantial but not statistically significant'. A focus in the results (effect estimate and measure of uncertainty) would strengthen this section.

**Author Response:** The secondary outcome section has been modified removing result interpretation.

**Alterations to Manuscript: Line: 404:** “…There was no evidence of a difference between groups in the number of steps taken per day from baseline to 6 months in the intervention group (mean between-group difference 646 steps per day, 95%CI -109 to 1402, n=239, p= 0.09) or on any other secondary outcomes (Tables 5, 6 and S3 Table).”

41. 349 - an interaction analysis is reported - was this the planned subgroup analyses.

**Author Response:** All analysis presented in this paper were pre-planned and specified in our Statistical Analysis Plan which was signed off prior to commencing data analysis. The interaction analysis is reported in our methods section: “We undertook six pre-specified subgroup analyses…”

**Alterations to Manuscript:** No alterations to manuscript.

42. 350-2 - there are exploratory analyses presented - the details need to be added to the methods - if post hoc, these need to be justified - could these be reported in another report so that this report focuses on the main trial analyses and outcomes?

**Author Response:** All analysis presented in this paper were pre-planned and specified and included in our Statistical Analysis Plan which was signed off on prior to commencing data analysis. We did discuss presenting the interaction analysis as a separate paper but felt the paper was stronger to include all our pre-specified analysis.

**Alterations to Manuscript:** No alterations to manuscript.

43. Discussion: Lines 355 to 358 - as a co-primary outcome was used, should the interpretation start with confirmation that there was no evidence of effectiveness of the intervention? The separate outcomes could then be discussed.

**Author Response:** This has been added to the beginning of the Discussion section.

**Alterations to Manuscript: Line 435: “**There was no evidence of effectiveness of the intervention in accordance with our pre-specified definition that both primary outcomes needed to show a statistically significant between group difference. However,….”

44. There is a tendency to focus on the significant results/timepoints. Suggest that the statistically significant results should be interpreted with caution as the results section highlights that there were limited differences across most outcomes and timepoints (and the issues with multiplicity - see below).

**Author Response:** As per your previous suggestion, we have added in the interpretation of a non-effective intervention based on our pre-specified criteria. We have also removed significance wording within the results section.

**Alterations to Manuscript: Line 435: “**There was no evidence of effectiveness of the intervention in accordance with our pre-specified definition that both primary outcomes needed to show a statistically significant between group difference. However,….”

45. Limitations: -multiplicity is a consideration

-the great deal of heterogeneity in the devices being tested and in the baseline characteristics of participants included is mentioned but I think the discussion would be enhanced with more discussion around these issues and their implications.

**Author Response:** The limitation of multiplicity has been added to the limitations paragraph.

**Alterations to Manuscript: Line 490: “**Study limitations include an anticipated 15-19% loss to follow-up at 6 months on co-primary outcomes, not unexpected in this age group of hospitalised patients. Multiplicity is also a consideration due to the number of outcomes measured. Additionally...”

**Reviewer 3 Comments to author:**

Reviewer #3: Thanks for the opportunity to read your paper. The study is relevant and interesting; the text is concise, methods and results clearly described. I look forward to reading the economic analysis also.

**Author Response:** We thank the reviewer for their considered comments in revising our manuscript.

I have only two items for clarification:

1. Line 441 noted that greater time was spent with therapists in the intervention group than the control group. Where are the data are that shows how much additional physiotherapy contact time the intervention group received, in comparison to the control group?

**Author Response:** Usual care was not routinely recorded at all sites, so we are unfortunately not able to provide an exact amount of extra therapy that was received by the intervention group. At the site that did routinely record usual care, we were able to show that there was no difference in the amount of usual care in both groups, therefore the additional dose as reported in Table 2 is the additional dose received by the intervention group.

**Alterations to Manuscript:** No alterations to manuscript.

2. Re: Line 355 - the first discussion point regarding …clinically important improvements in …self-reported time spent walking…not accompanied by changes in upright time. Can you comment on the apparent discrepancy between self-reported time spent walking and instrumented (ActivPAL) time spent upright? Also, the comparison between instrumented time stepping and self-reported walking times? e.g. Week 3: Instrumented time stepping = 44 min/day; instrumented time upright 208 min/day; IPEQ walking = 23 min/day (2.7 hrs/week) I would expect self-reported walking and instrumented stepping times to be similar, and upright time (assuming this is standing and walking) to be a lot less.

**Author Response:** All physical activity outcomes measure different aspects of physical activity, which is why they do not match. The activPAL activity monitor measures bodily movement whereas the IPEQ measures subjective recall of a range of different physical activities. Walking is not the same as stepping (which would include stepping of lower intensity than walking) and upright time is a mix of light (including standing) activities and MVPA. Even if the device measures and questionnaire were to measure the same physical activity aspects, a questionnaire would still give a different estimate than a device, mostly because of recall and social desirability bias in the questionnaire. Both device and questionnaire measures offer valuable and complementary insights into change in physical activity over the trial with device-measured uptime chosen as our primary outcome. A nice paper describing the issues of physical activity measurement is: Troiana R, et al. Reported physical activity and sedentary behavior: why do you ask? *J Phys ActHealth.* 2012;9:S68–S75.

Given self-reported walking is one of our secondary findings, we have moved the discussion about this finding further down in the Discussion section. There are some indications around improvements in activPAL steps and IPEQ walking, which do suggest that there are some improvements (especially in the younger participants), thus warrant discussion.

**Alterations to Manuscript:**

**Line 437:** Discussion: “However, significant and clinically relevant improvements in mobility were observed in participants receiving the AMOUNT intervention…”

**Line 473:** “Although the physical capacity of participants in the intervention group to move around improved, this did not translate to increased time spent upright. Yet there was an indication of more steps taken by intervention participants (p = 0.09), particularly younger participants (<76 years, p = 0.05), greater self-reported walking and more time spent stepping and less time spent standing compared to control participants.”

**AMOUNT rehabilitation trial: Study protocol versions**

Protocol versions:

| Protocol version number | Date approved by Southern Adelaide Clinical Human Research Ethics Committee (lead committee) |
| --- | --- |
| 1 | 19/12/2013 |
| 2 | 10/02/2014 |
| 3 | 29/05/2014 |
| 4 | 18/08/2014 & 25/11/2014 |
| **TRIAL RECRUITMENT COMMENCED SEPTEMBER 2014** | |
| 5 | 14/11/2014 |
| 6 | 30/06/2015 |
| Published final protocol | June 2016 |

**Differences between protocols:**

Differences between protocols 1 & 2:

- Development of Master Participant Information Sheet (PIS) to be used at all three recruitment sites
- Inclusion of an additional period of collecting activity levels for 7 days using the ActivPal (at baseline)
- Provision of a shortened title for the trial.

Differences between protocols 2 & 3:

- Change to inclusion criteria to include participants with cognitive impairment. Those with a score of >21 on the MMSE will be approached directly, and those with a MMSE score of <21 whom the treating clinician feels could benefit from this intervention will have the next of kin advised of the study and provided a PIS. A new proxy PIS and consent form has been developed for this purpose.
- Change to inclusion criteria to have a likely life expectancy greater than 6 months from 12 months
- Change to inclusion criteria to have the clinician assess capacity for improvement in mobility from the clinician considering the intervention to be appropriate for the individual.
- Change to exclusion criteria to remove the ability to balance safely in standing as the intervention provided will be one-to-one.
- Dose of intervention was changed from ‘at least 60 minutes per day’ to a more feasible ‘at least 30-60 minutes per day’ to improve adherence.
- More comprehensive list of equipment to be used in the intervention. The exercise protocol guide is to be reviewed quarterly in order to add new technologies throughout the trial.
- Some outcome measures were changed due to further investigation, discussion and trialling. Additional measures were: De Morton Mobility Index, Single leg stance, maximal balance range test, WHO Disability Assessment Scale 2.0, Telephone-Interview for Cognitive Status, Activities Specific Balance Confidence Scale, Incidental & Planned Exercise Questionnaire, Modified Computer Self-Efficacy Scale and the Functional Comorbidity Index.

Differences between protocols 3 & 4:

- 18/08/2014: Addition of a new measure of cognition, the Trail Making Test, which is a continuous measure and has the potential to show between group differences.
- 25/11/2014: Amendment of proxy PIS and consent form for Sydney sites as per instructions of NSW Guardianship Tribunal to change wording from ‘families/next of kin’ to ‘person responsible’ and to add a paragraph explaining what it means to be the person responsible in NSW.

Differences between protocols 4 & 5:

- Addition of honours sub-study: How hard do people work in therapy involving technology versus traditional therapy in rehabilitation?

Differences between protocols 5 & 6:

- Addition of mixed methods sub-study: Therapist preferences and experiences of implementing affordable technologies to improve physical activity levels and mobility outcomes in rehabilitation.

Differences between protocol 6 and published protocol:

- Assessment:
  - TICS measure of cognition which was planned for use in the study was removed prior to the study starting as the Trails A & B was a measure of cognition.
  - Baseline activPAL data was collected for 24 hours instead of 7 days to enable it to be measured prior to randomisation. This was implemented prior to the study starting.
- Intervention:
  - Physiotherapy support of participants in the community was initially recommended to be weekly, but was changed to weekly-fortnightly during the first few months of the trial to enable more tailored and flexible approach as supported by staff and participants.
