# Supplementary material for: Digitally enabled aged care and neurological rehabilitation to enhance outcomes with Activity and MObility UsiNg Technology (AMOUNT) in Australia: A randomised controlled trial
Source: PLoS Med. 2020 Feb 18;17(2):e1003029. doi: 10.1371/journal.pmed.1003029 (PMC7028259; doi:10.1371/journal.pmed.1003029)
Supplement: Attachment — Submitted filename: Response to Reviewer.docx [file pmed.1003029.s013.docx]

**Manuscript: Digitally-enabled rehabilitation to enhance outcomes: The Activity and MObility UsiNg Technology (AMOUNT) randomised controlled trial."**

**(PMEDICINE-D-19-02056)**

**Resubmission: Response to editor and reviewer comments, December 2019**

**Editor comments to Authors:**

1. We note the referee’s comments on inclusion of a cost effectiveness analysis. Having considered and discussed with my colleagues, we feel that inclusion of information on cost evaluation would strengthen the case for publication in PLOS Medicine and we request its addition to the manuscript.

**Author Response:** Information about the cost of the intervention has been added to the manuscript in the main text and as an additional table in the appendix (see at the end of this document). We are in the process of undertaking a full cost-effectiveness analysis but this is not yet complete. With the addition of the costing data, we would like to add Dr Marina Pinheiro (Institute for Musculoskeletal Health, Faculty of Medicine and Health; Discipline of Physiotherapy, Faculty of Health Sciences, The University of Sydney, Sydney, Australia) as author 20 to this paper. Dr Pinheiro is leading the cost effectiveness analysis under the mentorship of Professor Kirsten Howard (investigator and author on the main paper). Considerable work by Dr Pinheiro has gone into determining the costs of this intervention.

**Alterations to Manuscript: Line 349: “**Over the 6-month trial period, participants spent on average 19 days (SD 20; median 12) in an inpatient setting and 161 days (SD 18) in a post-hospital setting, typically at home. The total cost of the intervention (staff training, equipment, intervention preparation and delivery) per participant was AUD 1,892 (S2 Table).”

2. Data Availability: We will require clarification on data availability before publication. If the lead ethics committee do not permit distribution of de-identified data, we will require details for a point of contact (not an author) or a URL where researchers who meet requirements can apply to access the data. We will be unable to proceed with publication until our data requirements are met.

**Author Response:** The lead ethics committee is currently considering our request for distribution of de-identified data. If the ethics committee denies our request, Professor Chris Maher (Director of Institute for Musculoskeletal Health, Faculty of Medicine and Health, University of Sydney) has agreed to be the point of contact for the data.

3. Title: Please revise your title according to PLOS Medicine's style. It should begin with main concept if possible, followed by the study design in the subtitle (ie, after a colon). We suggest ‘Digitally-enabled aged care and neurological rehabilitation to enhance outcomes with Activity and MObility UsiNg Technology (AMOUNT) in Australia: A randomised controlled trial’

**Author Response:** We have revised our title in accordance with PLOS Medicine style.

**Alterations to Manuscript: Line 1: “**Digitally-enabled aged care and neurological rehabilitation to enhance outcomes with Activity and MObility UsiNg Technology (AMOUNT) in Australia: a randomised controlled trial”

4. Please remove all information within the footer of each page

**Author Response:** All information in the footer has been removed.

5. Lines 59-86 - Please remove all information presented here

**Author Response:** This information has been removed.

6. Please be explicit within your Abstract Methods and Findings when recruitment to the RCT took place.

**Author Response:** The abstract currently includes the dates when recruitment took place: **Line 81:** **“**Between September 22, 2014 and November 10, 2016, 300 patients (mean age 74, SD 14; 50% female) were randomly assigned …”

**Alterations to Manuscript:** No alteration to manuscript.

7. Please add to the Abstract Methods and Findings that the RCT was prospectively registered, providing the trial ID (rather than at the end of Abstract)

**Author Response:** The trial registration information has been moved to the Methods and Findings section of the abstract.

**Alterations to Manuscript: Line 80: “**The dataset was analysed using intention-to-treat principles. “The trial was prospectively registered with the Australian New Zealand Clinical Trials Register; ACTRN12614000936628. Between September...”

8. Please add summary demographic information for the participants of the study within your Abstract Methods and Findings

**Author Response:** Additional demographic information has been added to the Abstract.

**Alterations to Manuscript: Line 81: “**Between September 22, 2014 and November 10, 2016, 300 patients (mean age 74, SD 14; 50% female; 54% neurological condition causing activity limitation) were randomly assigned ….”

9. Line 93 - Please provide details on which cities the hospitals were in

**Author Response:** This information has been added to the abstract.

**Alterations to Manuscript: Line 70: “**We conducted a pragmatic, outcome assessor-blinded, parallel-group randomised trial in three Australian hospitals in Sydney and Adelaide recruiting adults with mobility limitations undertaking aged care and neurological inpatient rehabilitation.

10. Line 93 - Please provide details of the age range of adults recruited

**Author Response:** This information has been added to the abstract.

**Alterations to Manuscript: Line 69: “**We conducted a pragmatic, outcome assessor-blinded, parallel-group randomised trial in three Australian hospitals in Sydney and Adelaide recruiting adults 18 to 101 years old with mobility limitations undertaking aged care and neurological inpatient rehabilitation.”

11. Line 100 - Please add (SBBP) after the first mention of Short Physical Performance Battery

**Author Response:** This abbreviation has been added.

**Alterations to Manuscript: Line 76:** “Co-primary outcomes were mobility (performance-based Short Physical Performance Battery (SPPB); continuous version; ”

12. Line 111 - Please add a full stop after ‘...p=0·006)’ to break this up into 2 separate sentences

**Author Response:** This sentence has been broken into two sentences.

**Alterations to Manuscript: Line 91: “…**mean between-group difference 0·2 points; 95%CI 0·1 to 0·3; p=0·006). However, there was no evidence…”

13. Line 116 - please give an example or two of what ‘other secondary outcomes’ were

**Author Response:** Two examples of secondary outcomes have been added to the abstract.

**Alterations to Manuscript: Line 94: “**Scores were higher in the intervention group compared to the control group across most secondary mobility outcomes but there was no evidence of a difference between groups for most other secondary outcomes including self-reported balance confidence and quality of life.”

14. Line 119 - Please add a sentence to your study limitations that ‘The large number of outcome assessments in our trial increases the risk of a type I error’ or similar. Please also comment on whether provision of the devices could be described as a limitation, relating to possible real-world applicability of the intervention.

**Author Response:** This risk of type 1 error has been added to the abstract. We do not see the real-world applicability of the provision of devices to be a limitation as the majority are low cost and readily available for purchase. The intervention protocol (provided as a supplementary document) provides guidance for clinicians for using the range of devices. Recommendations for the next steps for research have been added to the discussion section as per editor comment 32, and this includes further work evaluating how clinicians can incorporate this into practice.

**Alterations to Manuscript: Line 100:** **“**Study limitations include an anticipated 15-19% loss to follow-up at 6 months on co-primary outcomes and the number of secondary outcome measures in our trial which may increase the risk of a type I error.”

15. Lines 144 & 178 - is there evidence to support that the intervention was enjoyable? If not, please remove.

**Author Response:** Line 144 relates to the results of the current study. Line 178 relates to the results of the feasibility trial conducted by our team (van den Berg, 2016). For both studies the Physical Activity Enjoyment Scale (PACES; 18 item scale, score 18-126) was used to assess enjoyment. In the feasibility study, “The scores on the Physical Activity Enjoyment Scale had a mean of 62 (SD 8), demonstrating that participants generally enjoyed the intervention.” In the current study, the PACES data is reported in the results section, with the data in Table 3. The mean (SD) score was 96(23) at 3 weeks, 96(22) at 12 weeks and 98(21) at 26 weeks, indicating enjoyment participating in the intervention. **Line 353**: “Intervention participants rated usability of prescribed devices above average, and enjoyment as high at all timepoints (Table 3).”

**Alterations to Manuscript:** No alteration to manuscript.

16. Line 148 - please omit ‘(0.9 point between group difference on the Short Physical Performance Battery 0 to 12 scale)’ from your Author Summary

**Author Response:** This part of the author summary has been deleted.

- **Alterations to Manuscript: Line 128: “**Clinically important improvements were seen in mobility at 3 weeks and 6 months after baseline, but this was not accompanied by greater time spent upright.”

17. Line 175 - please revise to ‘A feasibility trial conducted by our team in people undertaking inpatient aged care and neurological rehabilitation (n=58)’

**Author Response:** This sentence has been edited.

**Alterations to Manuscript: Line 155:** **“**A feasibility trial conducted by our team in people undertaking inpatient aged care and neurological rehabilitation (n=58) provided an additional dose of rehabilitation for 2-weeks using a range of low-cost videogames and activity monitors [13].”

18. Line 179 - please omit ‘now’

**Author Response:** This sentence has been edited.

**Alterations to Manuscript: Line 159:** **“**This promising intervention, after refinement, required rigorous evaluation.”

19. Please provide dates for recruitment (section beginning line 19) and randomisation (section beginning line 212)

**Author Response:** Dates for recruitment and randomisation are the same and are reported in the abstract (line 81) and in the results section of the main paper (line 322 “Between September 2014 and November 2016, 5,039 patients were screened, 715 patients were assessed as eligible and 300 patients provided informed consent and were randomised…”).

**Alterations to Manuscript:** No alteration to manuscript.

20. Line 228 – Is the intervention protocol part of the prespecified protocol?

**Author Response:** The intervention protocol was developed prior to the study commencing but, as planned, was refined as the study progressed. All research staff delivering the intervention were trained using the intervention protocol. As described in table 1: “As planned, the intervention protocol was modified during the trial; version 2 published 14.10.15 and version 3 published 23.02.16…”

**Alterations to Manuscript:** No alteration to manuscript.

21. Line 232 – Who paid for the devices that cost $3700? In Table 1 it appears that devices were ‘loaned’; this should also be mentioned in the main text on first mention.

**Author Response:** The devices were purchased by the research team. This has been added to the manuscript. The loaning of devices to participants has been added to the main text of the manuscript.

**Alterations to Manuscript: Line 212: “**Included devices were purchased by the research team or constructed for <USD$3700 each. Participants could use any number of devices as guided by the physiotherapist. Devices were loaned to participants to use at home and were progressed or changed as required.

22. Table 1 & line 286 – For face-to-face sessions and the IPEQ, please provide the questionnaires / interview material (and any others used) as supplementary files.

**Author Response:** The IPEQ has been included within the Statistical Analysis Plan as a Supplementary file. All other questionnaires used are published questionnaires and may be subject to copyright to reproduce without permission. Study developed practice sheets are included within the Intervention protocol which has been included as a Supplementary file.

**Alterations to Manuscript:** No alteration to manuscript.

23. Table 1 – Why were the modifications planned?

**Author Response:** Modifications were planned as it was anticipated that devices would change over the course of the trial. The intervention protocol was developed as a way to use digital devices to provide additional training of task specific practice and more broadly physical activity, rather than to test a specific device. We see this as a strength of our study.

**Alterations to Manuscript:** No alteration to manuscript.

24. Line 312 – Please state modifications to the analysis plan (and why) and refer to the supplementary file in your methods section.

**Author Response:** The supplementary file has been referred to in the methods section. No changes were made to the statistical analysis plan once it was signed off. This has been more clearly stated in the manuscript.

**Alterations to Manuscript:** Line 294: “A statistical analysis plan was approved by the study statistician (SH) and Chief Investigator (CS) before data analysis and no changes were made after this time (see S2 SAP).”

25. Line 340 - please clarify whether this was written informed consent.

**Author Response:** This information was provided in the methods section but has also now been added to the results section.

**Alterations to Manuscript: Line 322: “**Between September 2014 and November 2016, 5,039 patients were screened, 715 patients were assessed as eligible and 300 patients provided written informed consent”.

26. Table 3 - please provide definitions for all abbreviations used

**Author Response:** Additional definition of an abbreviation has been added.

**Alterations to Manuscript: Line 346:** EuroQOL-5L: European Quality of Life-5.

27. Tables 5, 6, S2, S3, S5, S6 - When a p value is given, please specify the statistical test used to determine it.

**Author Response:** Our analysis used linear regression models. This information is included underneath the tables: “This analysis was conducted using linear regression models with baseline scores entered as covariates. Due to skewed distributions, the change score between timepoints was used for all outcomes except proportion of the day spent upright. Confidence intervals have not been adjusted for multiplicity, so inferences drawn from the intervals may not be reproducible. ~between group difference presented as odds ratios; significant interactions (p≤ 0.05) for pre-specified variables at these timepoints.”

**Alterations to Manuscript:** No alteration to manuscript.

28. Line 435 - Please revise this paragraph as ‘no evidence of effectiveness’ followed by ‘significant and clinically relevant improvements’ appears contradictory on first reading.

**Author Response:** This section was modified in line with previous reviewer comments: **“**Discussion: Lines 355 to 358 - as a co-primary outcome was used, should the interpretation start with confirmation that there was no evidence of effectiveness of the intervention? The separate outcomes could then be discussed.”

We would be happy to remove the first sentence if you feel it would be clearer to the reader: **Line 421**: “Significant and clinically relevant improvements in mobility were observed in participants receiving the AMOUNT intervention.

**Alterations to Manuscript:** No alteration to manuscript.

29. At line 443, you mention that “six” of the devices were used across care settings, but it appears from table 4 that a mean of 2 devices were used across the two settings. Please revise the wording as appropriate.

**Author Response:** This is correct. From Table 4, there was a mean (SD) of 4(1) different devices used per participant in the inpatient setting and 2(1) in the post-hospital setting. Six of the eight available devices were used by participants in both the inpatient and post-hospital setting. The Humac and the Stepping Tiles were only available for use in the inpatient setting.

**Alterations to Manuscript:** No alteration to manuscript.

30. Line 448 - Please provide justification for how results can be considered of clinical importance if ‘clinically important difference has not been established for this version of the SPPB’

**Author Response:** This statement is justified in the paragraph following this paragraph. “The mean between-group difference on our primary mobility measure at 3 weeks (0.3 points) and 6 months (0.2 points) can be considered of clinical importance. Although a clinically important difference has not been established for this version of the SPPB, at both timepoints the difference was greater than what has been reported in the literature as the amount of deterioration over a 1-year period in older women living in the community with disability (0.15 points) [18]. In addition, on the secondary mobility measure of the SPPB total score (0-12), the between-group difference was greater at both timepoints (0.9 points) than the clinically important difference of 0.5 points [20].”

**Alterations to Manuscript:** No alteration to manuscript.

31. Line 493 - Please revise the sentence ‘there was no statistically significant difference in important participant-reported outcomes such as health related quality of life; however, all these outcomes were favourable’ to clarify your findings / make explicit whether significant differences were seen or not.

**Author Response:** This sentence has been revised.

**Alterations to Manuscript: Line 478:** Additionally, there was no statistically significant difference in important participant-reported outcomes such as health-related quality of life; however, all these outcomes were in the direction favouring the intervention group, which may reflect low statistical power to demonstrate significance for these outcomes.

32. Please add a short section on the implications and next steps for research, clinical practice, and/or public policy between your limitations and conclusion summary.

**Author Response:** An additional paragraph has been added on the implications and next steps for research.

**Alterations to Manuscript: Line 494:** Further research should investigate whether future models of rehabilitation care can incorporate digital devices to enhance inpatient and post-hospital rehabilitation with a higher dose of practice whilst conserving quality. Hybrid type II implementation-effectiveness study designs [43] could be used to simultaneously test the effectiveness of the clinical intervention (digitally-enabled rehabilitation) on patient outcomes and the effectiveness of implementation strategies (e.g. education and training) to support clinicians to include digital devices into practice.

33. Please include paragraph numbers (instead of page numbers) in your CONSORT statement

**Author Response:** A new version of the CONSORT checklist has been uploaded with page and paragraph numbers included.

34. Please remove files Manuscript under review_1 and Manuscript under review_2

**Author Response:** These two files have been removed.

**Reviewer 1 Comments to Authors:**

Reviewer #1: The authors have appropriately responded the all the reviewers comments. The re-review of the methods and reporting of the trial methods and results are much improved and clearer. This is a high quality trial, with well-reported findings, and clinically relevant findings to the rehabilitation field. I have recommended acceptance for publication on that basis. Interestingly, the reviewers have all highlighted that a much more detailed economic analysis will be presented separately which I will very much look forward to seeing.

1. The only comment I have remaining is regards to a comment regarding balance between arms after randomisation. I take the authors point that baseline statistical testing is not warranted and opposed by CONSORT (and randomisation has been reported and conducted appropriately). The question I had remaining was whether, from a clinical perspective whether the authors felt there could be potentially any influencing participant factors (if any), that would they would view as clinically relevant. Given that some imbalance occurs by chance, whether any these factors should or could be explored further perhaps in a post-hoc analyses as covariate adjustments to explore the influence on the results. Altman's 1985 paper has a few examples of exploring covariate adjustment principles by strength of prognostic factors but these are mostly decisions guided by clinical expertise and experience: "Comparability of Randomised Groups." Journal of the Royal Statistical Society. Series D (The Statistician), vol. 34, no. 1, 1985, pp. 125-136. JSTOR, [www.jstor.org/stable/2987510](http://www.jstor.org/stable/2987510). It would be useful to get the authors view on this.

**Author Response:** When planning for the statistical analysis, we included sub-group analyses so that we could explore the influence of demographic and baseline variables that we felt may influence our results. The variables we included were neurological versus non-neurological health conditions, sex, age, baseline mobility, device use prior to hospital admission and Australian state. As reported in our results section: “Interaction analysis for primary outcomes indicated a greater effect of the intervention on mobility among those with poorer mobility at baseline (Table 5 and S5 Table). Exploratory analyses for secondary outcomes revealed consistently greater intervention impact in younger participants (Tables 5, 6 and S6 Table).”

**Alterations to Manuscript:** No alterations to manuscript.

**Reviewer 2 Comments to author:**

Reviewer #2: The authors have done an excellent job responding to the comments. The manuscript is substantially improved and a stronger communication of the methods and results of this important trial. I only have a few mainly minor suggestions for the authors to consider:

**Author Response:**

1. Point 3 response: Instead of 'with allocation concealment' it would be more informative to state the method, such as 'assigned using a remotely prepared web-based randomisation system to…'

**Author Response:** We have added this additional information to the abstract.

**Alterations to Manuscript: Line 81: “**Between September 22, 2014 and November 10, 2016, 300 patients (mean age 74, SD 14; 50% female; 54% neurological condition causing activity limitation) were randomly assigned using a secure online database (REDCap) to achieve allocation concealment to intervention (n=149) or control (n=151). …”

2. Point 19 response: The extra detail in the response about clinician assessed capacity for improvement is informative. It means that a pragmatic assessment was used, incorporating clinical judgement. I think clarification of this detail would be a useful addition to the paper and would aid generalisability.

**Author Response:** Additional information has been added to the inclusion criteria to better describe this criteria.

**Alterations to Manuscript: Line 181: “**Consecutive patients admitted to the units who met the following criteria were invited to participate: ≥18 years; reduced mobility (Short Physical Performance Battery score <12) [15] with clinician assessed capacity for improvement (based on the usual care physiotherapist’s clinical experience and their assessment and treatment experience with the patient); life expectancy >12 months;**….”**

3. Point 27 response: As the selected 15% difference was selected based on experience of what would be clinically important, it would be useful background to explain this in the sample size section. The relationship between the 15% clinically meaningful difference used in the sample size calculation and the effect sizes observed in the SPPB would be a useful point for the discussion to aid interpretation of the clinical importance.

**Author Response:** We have added this additional information to the sample size section.

**Alterations to Manuscript: Line 290: “**This sample size was also estimated to be sufficient to detect between-group differences of 10-15% in most secondary outcomes and considered by the authors’ to be of meaningful size on the basis of our collective clinical experience with the measures**.**”

4. Author summary: are the +/- SDs?; the 12 point SPPB results are in the what this study adds section, rather than the primary outcome (0-3 continuous SPPB)- would suggest the primary outcome data would be more appropriate.

**Author Response:** As per editor comments, we have moved the data from this part of the author summary.

- **Alterations to Manuscript: Line 128:** “Clinically important improvements were seen in mobility at 3 weeks and 6 months after baseline, but this was not accompanied by greater time spent upright.”

| **S2 Table. Costs for digitally-enabled rehabilitation intervention** | | | |
| --- | --- | --- | --- |
| **Items** | **Description** | **Total cost** | **Cost / participant^1^** |
| **Training** | Costs with registration for staff to attend training course, staff time to attend training course, workshops and meetings | $32,894 | $230 |
| **Equipment** | Costs with equipment**^2^** used in the trial (eg activity monitors, Nintendo Wii, iPads, sim cards, consumables, maintenance and repairs) | $50,616 | $354 |
| **Intervention delivery - Inpatient** | Staff time**^3^** to deliver the intervention at the hospital (time taken on session delivery and preparation for session) | N/A | $621 (SD = 688, range 0 to 3,348) |
| **Intervention delivery - Outpatient** | Staff time**^3^** to deliver the intervention after hospital discharge (session delivery, phone call, email, in travel, home visit, video conference as well as preparation for treatment session) and costs with SMS**^4^**, phone call**^5^**, and travel.**^6^** | N/A | $ 687 (SD 302, range 0 to 2,240) |
| **Total cost** |  | N/A | $1,892 (SD 774, range 908 to 4399) |

^1^Considering 143 participants in the intervention group (excluded participants who died or dropped-out before the 3-week follow-up). All costs were valued in 2017 Australian dollars (AUD).

^2^The annual equivalent cost was calculated for each equipment and the value was divided by 2 (to reflect the 6-month trial duration). We assumed that all equipment used had no re-sale value.

^3^Staff costs were calculated according to staff level using actual data. The public hospital award wages for physiotherapists relevant for each hospital were used. Based on trial records and physiotherapists feedback, we considered the preparation time to be: 20 minutes for inpatient sessions, 30 minutes for home visits, 10 minutes for SMS, email, phone and video conference.

^4^Cost of SMS extracted from SMS Global (example of provider, [www.smsglobal.com](https://www.smsglobal.com/)): $0.10 / SMS.

^5^Costs estimated from OPTUS (example of provider, [www.optus.com.au](http://www.optus.com.au)) assuming that all calls were made to mobile numbers: $0.28 per minute plus $ 0.52 connection fee per call).

^6^Cost of travel extracted from Australian Tax Office and estimated as $0.66 per Km.
