# Supplementary material for: Digitally enabled aged care and neurological rehabilitation to enhance outcomes with Activity and MObility UsiNg Technology (AMOUNT) in Australia: A randomised controlled trial
Source: PLoS Med. 2020 Feb 18;17(2):e1003029. doi: 10.1371/journal.pmed.1003029 (PMC7028259; doi:10.1371/journal.pmed.1003029)
Supplement: Attachment — Submitted filename: Response to Reviewer_20Dec2019.doc [file pmed.1003029.s014.doc]

**Manuscript: Digitally-enabled aged care and neurological rehabilitation to enhance outcomes with Activity and MObility UsiNg Technology (AMOUNT) in Australia: a randomised controlled trial**

(PMEDICINE-D-19-02056R2)

**Resubmission: Response to editor comments, December 20^th^ 2019**

**Editor comments to Authors:**

1. Thank you for adding to your revised manuscript information regarding the cost of the intervention, which we feel has improved the manuscript.
2. We note the addition of a new author. PLOS acts according to COPE guidelines, and so requires that any changes to the author listing on a manuscript are confirmed and agreed by all authors. All authors must fulfill the ICMJE criteria for authorship, and meet the CRediT guidelines for contributions, as described here: <https://protect-au.mimecast.com/s/5I9MCOMxNyt75jwpHkXulP?domain=journals.plos.org>.

Please could you, as corresponding author, contact all authors - including the authors being added - to obtain their emailed confirmation that they agree to this change and the contributions made by the proposed new author? You should then forward all the confirmations to us in one file.

**Author Response:** I have received emails from 20 of the 21 co-authors to confirm that they approve the addition of Dr Marina Pinheiro as an author on the AMOUNT trial. A combined document of the co-author email approval has been uploaded to the system. I will forward an updated version to your editorial office as soon as I hear from the final co-author (A/Prof Heritier) who is currently on leave until 7^th^ January.

3. Thank you for confirming that access to de-identified data is still being considered by the lead ethics committee, and for suggesting an alternative contact (not an author) in the event that permission is not granted by the ethics committee. We will not be able to publish this work until this issue resolved.

**Author Response:** We have received approval from the lead ethics committee to provide the de-identified data in an open access repository. The data will be stored in the Sydney eScholarship repository, which is the University of Sydney's open access institutional repository. We have submitted a request to have our data added to the repository and for it to be under embargo until the date the paper is published in PLOS Medicine. This request has been accepted and will be actioned when the University of Sydney reopens next year from the 7^th^ January.

4. We note that some of the modifications to the intervention protocol have been summarised in Table 1. Please provide the three versions published during the trial in the supplementary files.

**Author Response:** No information was removed from the intervention protocol; rather additional information was added with new games/exercises or devices available for use. We therefore feel it will be replication and potentially confusing for people accessing the Supplementary materials to have three versions. We therefore prepared similar information to what we did for the change in study protocols to indicate the exact changes from version 1 to version 2 to version 3. This has been added as part of the Supplementary Material and referred to in Table 1. A copy of this information is below.

5. You mention at line 479-481 that not finding “statistically significant difference in important participant-reported outcomes … may reflect low statistical power to demonstrate significance”. Please add this is as a limitation to the final sentence of your Abstract: Methods and Findings.

**Author Response:** This information has been added to the abstract.

**Alterations to Manuscript: Line 96: “**Study limitations include an anticipated 15-19% loss to follow-up at 6 months on co-primary outcomes, the number of secondary outcome measures in our trial which may increase the risk of a type I error and potential low statistical power to demonstrate significant between group differences on important secondary patient-reported outcomes.”

6. Line 174 - please specify the cities in which the hospitals were located (as you have in your Abstract)

**Author Response:** This information has been added.

**Alterations to Manuscript: Line 169: “Sites, staff and participants**

There were three trial sites across Australia. Two were in metropolitan hospitals in Sydney in New South Wales (Site 1. 20 bed stroke and 20 bed aged care rehabilitation wards; Site 2. 16 bed brain injury rehabilitation unit) and one in Adelaide in South Australia (Site 3. 30 bed Geriatric Evaluation and Management (GEM) ward, and 40 and 20 bed general rehabilitation wards).”

7. Line 231 – please clarify whether a specific amount of time spent upright was required in order to record ‘upright time’ as an outcome.

**Author Response:** the activPAL default minimum posture allocation period (upright or non-upright) is 10 seconds. This information has been added to the manuscript.

**Alterations to Manuscript: Line 244: “**The measure of physical activity was ‘upright time’, defined as the average proportion of the day spent standing and stepping, measured in 10 second minimum periods.”

8. Please provide references to published questionnaires used in the study.

**Author Response:** All published questionnaires used in the study have a reference included, see line 169-287, references 29 to 39.

9. Line 429 - please revise to "Six of the devices were used across both inpatient and post-hospital care settings"

**Author Response:** This change has been made.

**Alterations to Manuscript: Line 428: “**Six of the devices were used across both inpatient and post-hospital care settings and usability and enjoyment were rated highly.”

10. Line 448 – please replace ‘favourable’ with another term that clarifies the meaning of the sentence ‘Our results are more favourable than those of the Cochrane systematic review of virtual reality interventions in people after stroke’.

**Author Response:** This sentence has been edited to provide greater clarification.

**Alterations to Manuscript: Line 444:** Our findings of improved mobility are consistent with previous systematic reviews demonstrating improved activity when a greater amount of task-specific practice is provided [4, 5]. In contrast, our findings of improved mobility are different to those of the Cochrane systematic review of virtual reality interventions in people after stroke for the comparison of additional virtual reality intervention on global motor function [11].”

11. We note your response to our previous request to ‘Please provide justification for how results can be considered of clinical importance if ‘clinically important difference has not been established for this version of the SPPB’ Your explanation is still not clear; please clarify what exactly is meant by the comparison of your results with “the amount of deterioration over a 1- year period in older women living in the community with disability”, when this compares improvements in your primary mobility measure with worsening of mobility through deterioration. 0.2 points at 6 months compared to 0.15 points at 1 year does not appear convincingly clinically important to a non-expert reader.

**Author Response:** The editor makes a valid point and we have changed this text.

**Alterations to Manuscript: Line 425: “**Between-group differences were still evident at 6 months despite the lower intensity physiotherapy support in the post-hospital period…”

**Line 434: “**The mean between-group difference on our primary mobility measure at 6 months (0.2 points) may be considered of clinical importance. A value of 0.54 on the 12-point version of the SPPB scale has been suggested to be a small meaningful change i.e., 4.5% of the maximum value [20]. The between-group difference at 6-months in the present study represents 6.7% of the maximum value for the 3-point version, therefore it may represent meaningful change.”

12. Please indicate the relevant sections (e.g. Methods, etc) and paragraph numbers (instead of page numbers) in your CONSORT statement. As an online journal, papers published in PLOS Medicine do not have page numbers.

**Author Response:** This change has been made.

**AMOUNT Intervention Protocols**

1. Original intervention protocol, dated 14^th^ August 2014.
2. Intervention protocol V2, dated 14^th^ October 2015.
3. Intervention protocol V3, 23^rd^ February 2016.

**Differences between protocols:**

Differences between protocols 1 & 2:

- Additional information was added to version 2 to further describe the post-hospital setting and the provider of the intervention. The frequency of the post-hospital support was initially prescribed weekly but changed within the first 6 months of the trial to “as required” with a recommendation of weekly initially, reducing the frequency over time if the participant was managing well. This was modified due to experience in the trial and matched the tailored intervention. This modification was reflected in version 2 (red writing is additional information in version 2).

## Setting

- **Version 1:** Inpatient for initial intervention, progressing to home-based once discharged. The inpatient intervention will be conducted within the physiotherapy rehabilitation gyms of the site hospitals.
- **Version 2:** The inpatient intervention will be conducted within the physiotherapy rehabilitation gyms of the site hospitals. The community-based program will commence once discharged from a rehabilitation ward and can include home, transitional living ward or residential care settings.

## Provider of intervention

- **Version 1:** The research physiotherapist will determine the most appropriate intervention based on discussion with the clinical physiotherapist, baseline assessment, participant goals, and the technology suitable in accordance with the intervention protocol. The research physiotherapist will then provide the inpatient intervention one-on-one for the length of their inpatient rehabilitation, and then using phone email or Skype contact weekly once they are home (a home visit may be required to set up the technology prior to discharge, and up to 5 home visits may be provided during the trial if there are problems with the technologies or if technologies are upgraded).
- **Version 2:** The research physiotherapist will determine the most appropriate intervention based on discussion with the clinical physiotherapist, baseline assessment, participant goals, and the technology suitable in accordance with the intervention protocol. The research physiotherapist will provide the inpatient intervention one-on-one for the length of their inpatient rehabilitation, and then support the community program by using phone, email, or video conference contact once they are home. A home visit may be required to set up the technology prior to discharge, and up to 5 home visits may be provided during the program if there are problems with the technologies or if technologies/program needs upgrading.

## Frequency

- **Version 1:** ≥5 days per week
- **Version 2:** Participants should participate in technology-based intervention ≥5 days per week for the length of the program. The research physiotherapist will provide one-on-one intervention 5x per week during the inpatient stage. The research physiotherapist will provide support and health coaching as required by each participant during the community stage. It is recommended support is provided weekly to begin with, but the frequency can reduce if the participant is managing their program well.
- No information from version 1 was taken away when the protocol was updated. The following information was added to version 2.
  - Getting started with Wii Family Trainer and Wii Family Trainer games
  - Nintento WiiU instructions for set up and use and Nintendo WiiU games.
  - Information about Fitbit Charge model added to protocol.
  - Information about Garmin Vivofit model added to protocol.
  - Information about Investigator developed Walk Forward phone App
  - Inpatient practice sheet updated
  - Community intervention cover sheet
  - Initial health coaching session
  - Intervention technology support sheet
  - Community goal setting sheet updated

Differences between protocols 2 & 3:

- Software update versions for Humac, Fysiogaming and AMOUNT App
- Additional games added to Fysiogaming table
- Instruction on wearing Fitbit updated for slower walkers.
